# Exosome-coated oxygen nanobubble-laden hydrogel augments intracellular delivery of exosomes for enhanced wound healing

**DOI:** 10.1038/s41467-024-47696-5

**Published:** 2024-04-23

**Authors:** Xiaoxue Han, Chaimongkol Saengow, Leah Ju, Wen Ren, Randy H. Ewoldt, Joseph Irudayaraj

**Affiliations:** 1 Department of Bioengineering, 1102 Everitt Lab, 1406 W. Green St., University of Illinois at Urbana-Champaign, Urbana, IL 61801 USA; 2 Biomedical Research Center, Mills Breast Cancer Institute, Carle Foundation Hospital, Urbana, IL 61801 USA; 3 Cancer Center at Illinois, Beckman Institute, Urbana, IL 61801 USA; 4 Holonyak Micro and Nanotechnology Laboratory, Carle R. Woese Institute for Genomic Biology, Urbana, IL 61801 USA; 5 Department of Mechanical Science and Engineering, University of Illinois at Urbana-Champaign, Urbana, IL USA; 6 Carle Illinois College of Medicine, University of Illinois at Urbana-Champaign, Urbana, IL 61801 USA

**Keywords:** Drug delivery, Biomedical engineering

## Abstract

Wound healing is an obvious clinical concern that can be hindered by inadequate angiogenesis, inflammation, and chronic hypoxia. While exosomes derived from adipose tissue-derived stem cells have shown promise in accelerating healing by carrying therapeutic growth factors and microRNAs, intracellular cargo delivery is compromised in hypoxic tissues due to activated hypoxia-induced endocytic recycling. To address this challenge, we have developed a strategy to coat oxygen nanobubbles with exosomes and incorporate them into a polyvinyl alcohol/gelatin hybrid hydrogel. This approach not only alleviates wound hypoxia but also offers an efficient means of delivering exosome-coated nanoparticles in hypoxic conditions. The self-healing properties of the hydrogel, along with its component, gelatin, aids in hemostasis, while its crosslinking bonds facilitate hydrogen peroxide decomposition, to ameliorate wound inflammation. Here, we show the potential of this multifunctional hydrogel for enhanced healing, promoting angiogenesis, facilitating exosome delivery, mitigating hypoxia, and inhibiting inflammation in a male rat full-thickness wound model.

## Abstract (web summary)

Challenges including hypoxia, inflammation, and inadequate exosome intracellular delivery hinder wound healing. Here, the authors show that a multifunctional exosome-coated oxygen nanobubble-laden hydrogel enhances exosome delivery and mitigates hypoxia for enhanced wound healing.

## Introduction

Poor wound healing following trauma and surgical procedures constitutes a pressing global medical issue, impacting millions of individuals annually and posing substantial challenges to healthcare professionals^1,2^. This concern arises due to the adverse effects on patients’ quality of life, heightened psychosocial stress, and the considerable financial burden associated with prolonged clinical wound management. The statistics reveal that inadequate wound healing affects 6.5 million patients in the US, and among all wound types, surgical wounds, being the costliest, significantly contribute to total Medicare spending^3^. The main goals in managing wounds encompass expeditious wound closure and the attainment of a scar that is both functional and aesthetically pleasing^4–6^. However, anomalous wound healing can be triggered by the deviance of inflammation and hypoxia from their typical patterns during the normal healing process, thereby inducing delayed wound closure, keloid formation, and the development of hypertrophic scars^7,8^. Therefore, effective management of hypoxia and inflammation is of paramount importance in the development of next generation advanced wound dressings.

Adipose-derived stem cell (ADSC)-derived exosomes have emerged as promising therapeutic agents in tissue regeneration. This is primarily attributed to their capability to function as intercellular communicators and harbor a rich cargo of bioactive molecules, including proteins, nucleic acids, and lipids. These molecules contribute to wound healing by exerting anti-inflammatory effects, inhibiting apoptosis, promoting angiogenesis, and facilitating enhanced cell migration and proliferation^9,10^. Recent studies emphasize the potential of exosome-loaded hydrogels as multifunctional dressings for both acute and chronic wound healing^11,12^. The data showcases their capability to mitigate oxidative stress, stimulate angiogenesis, and enhance fibroblast migration, contributing positively to all phases of the wound healing process^13–15^. Notably, exosomes offer several advantages over stem cells, as they are considered safer, lack tumorigenic potential, and pose a minimal risk of embolism^16,17^. Nevertheless, recent investigations have revealed that the intracellular cargo delivery efficiency of exosomes is compromised under wound hypoxic conditions due to activated hypoxia-induced endocytic recycling^18,19^. This limitation significantly impedes their therapeutic efficacy and necessitates approaches to enhance exosome delivery in hypoxic wound tissues.

To address this challenge as well as wound inflammation and hypoxia, we propose a strategy utilizing ADSC-derived exosome coated BSA-based oxygen nanobubbles (EBO) embedded within a self-healing hydrogel matrix as a multifunctional wound dressing. The oxygen supply component, oxygen nanobubbles (ONB), is formed by encapsulating nanoscale oxygen bubbles within a glycosylated protein conjugate composed of dextran-conjugated bovine serum albumin (BSA). The utilization of glycosylated proteins offers enhanced protein characteristics, including improved thermal stability and self-assembly properties^20^. Additionally, the glycosylated protein conjugates have been established to have free radical scavenging features and the capacity to impede oxidative deterioration, rendering them extensively useful in biomedical applications such as tumor therapy and wound healing^21^. Simultaneously, the glycosylated protein conjugate encapsulates the bulk nanoscale oxygen bubbles produced through ultrasonic cavitation, resulting in the formation of ONB. Exosomes derived from human adipose tissue stem cells are further coated onto the surface of the ONB, resulting in the final oxygen-carrying nanosystem, which exhibits oxygen release properties and facilitates the intracellular delivery of exosomes.

**Fig. 1:**
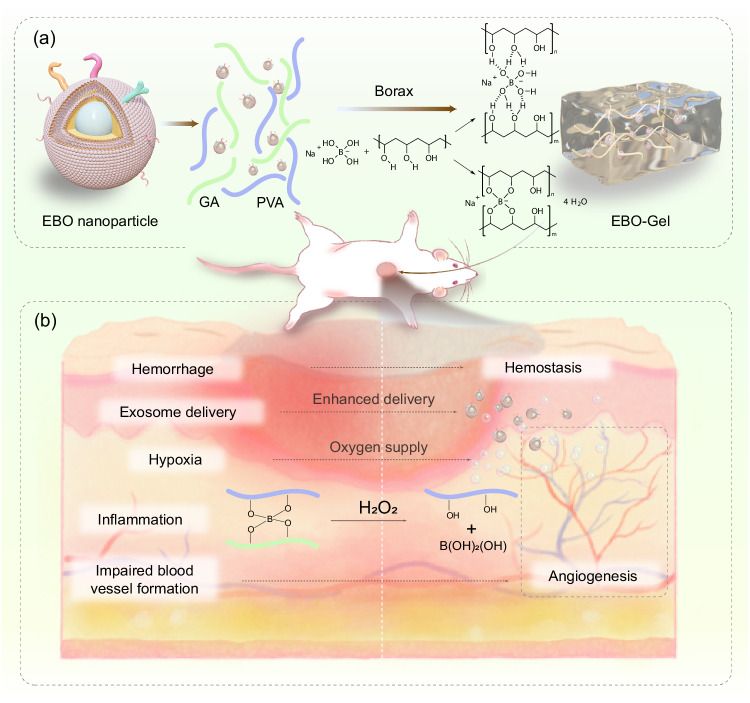
Schematic illustration of EBO-Gel-mediated wound healing. **a** Crosslinking mechanisms and structure of EBO-Gel. **b** Enhanced wound healing programmed by hemostasis, promoted exosome delivery, oxygen supply, angiogenesis, and antioxidant properties offered by EBO-Gel. PVA polyvinyl alcohol, GA gelatin, EBO ADSC-derived exosome coated BSA-based oxygen nanobubbles, EBO-Gel EBO nanoparticles-embedded hydrogel.

To further optimize the hemostatic and antioxidant properties of wound dressings, a hybrid hydrogel is developed by combining polyvinyl alcohol (PVA), gelatin (GA), and borax. The hydrogel possesses dynamic crosslinking through chemical borate ester bonds, imparting them with exceptional tissue adhesion, self-healing capabilities, and shape adaptability^22^. The mechanism that is responsible for the self-healing ability of the PVA/GA hydrogel is the boronic crosslinks. Two possible routes were presumed that depicts intraspecies (PVA-PVA) and interspecies (PVA-GA) crosslink: (i) boronic-esters^23^; and (ii) ionic crosslinks^24^ (Fig. 1a). Consequently, it is well-suited for wounds that require frequent stretching and for use in parts of the body that are in constant motion and can provide protection to wounds by effectively sealing the wound site, reducing the risk of further injury or contamination^25^. Moreover, the borate bonds present in the hydrogel exhibit the unique ability to react with hydrogen peroxide (H_2_O_2_), thereby mitigating excessive inflammation^26^. Additionally, the incorporation of gelatin, known for its hemostatic properties, further enhances the dressings’ ability to promote blood clotting (Fig. 1b), facilitating the accelerated healing of traumatic and surgical wounds^27^.

In this work, we show the feasibility of integrating an oxygen-carrying nanobubble, EBO, into a wound dressing to facilitate therapeutic exosome delivery by incorporating an antioxidative and hemostatic self-healing PVA/GA gel as a scaffold. In the rat full-thickness wound model, the hydrogel demonstrates accelerated wound healing and reduced inflammation. This advanced dressing synergistically combines multiple functionalities, including hemostasis, antioxidant activity, oxygen delivery, and enhanced exosome transport.

## Results

### Synthesis and characterization of EBO

**Fig. 2:**
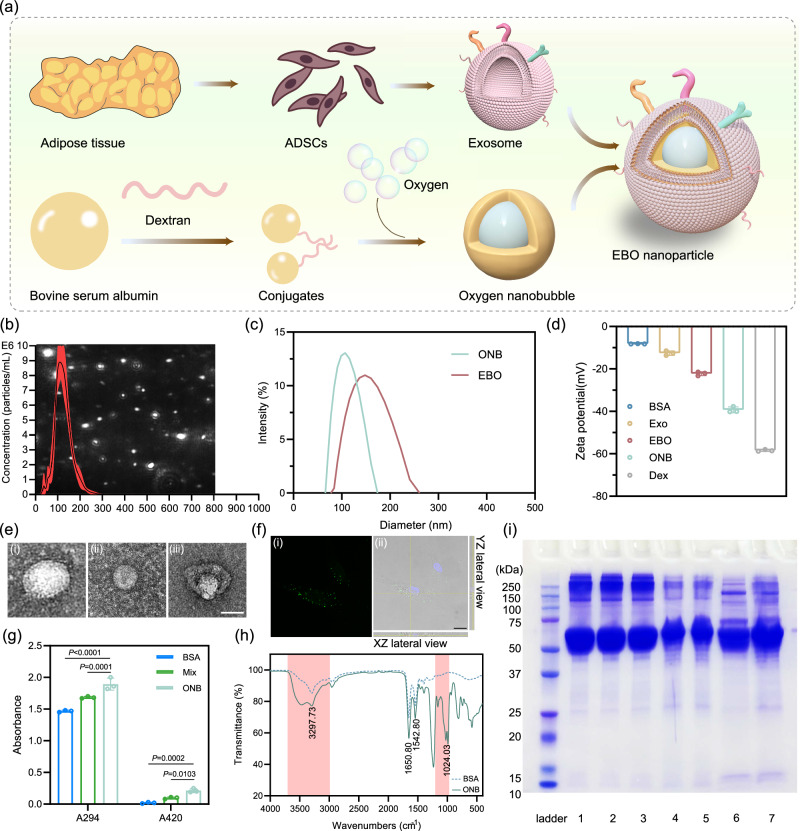
Synthesis and characterization of EBO. **a** Schematic of the preparation of ONB and EBO. **b** Concentration distribution and scattered images (background) of exosomes. Diameter distribution (**c**) and zeta potential values (**d**) of ONB and EBO ($n$  =  3 independent samples). **e** TEM images of (i) exosomes; (ii) ONB; (iii) EBO. Scale bar: 50 nm. **f** (i) EBO uptake and (ii) Z-stack slice with orthogonal views of EBO (Green) internalization in HDF-a cells after 6 h of incubation. Scale bar: 10 μm. **g** The browning intensity of early (A_294_) and late (A_420_) MRP ($n$ =  3 independent samples). **h** Infrared spectra of BSA and ONB. Pink areas indicate the spectral regions of group vibrations (Left) and dextran (Right). (i) SDS-PAGE of different formulations. Lanes: 1. Natural BSA; 2. Mixture of BSA and dextran sulfate; 3. Ultrasonicated BSA; 4. Shell; 5. ONB; 6. Exosomes; 7. EBO. Data are presented as mean ± SD (**b**, **d**, **g**). Statistical analysis was performed by two-way ANOVA with Dunnett’s multiple comparisons (**g**). Representative images are shown from two independent experiments with similar results (**e**, **f**, **i**). Source data are provided as a Source Data file. BSA bovine serum albumin, ONB oxygen nanobubble, Exo adipose-derived stem cell (ADSC)-derived exosomes, EBO ADSC-derived exosome coated BSA-based oxygen nanobubbles, Dex dextran sulfate, Mix mixture of BSA and dextran sulfate.

Adipose tissue serves as an indispensable reservoir of mesenchymal stem cells for applications in tissue engineering and stem cell therapy due to its convenient accessibility through minimally invasive procedures in human subjects^28,29^. In this study, we isolated ADSCs from human adipose tissue and proceeded to extract and characterize exosomes derived from these ADSCs. The isolated ADSCs exhibited the characteristic spindle shape and fibroblast-like growth pattern commonly associated with mesenchymal stem cells (Supplementary Figs. 1 and 2). Furthermore, these cells demonstrated positive expression of a panel of mesenchymal stem cell marker (CD90, CD105, and CD44), while displaying negative expression of CD106, CD45, and CD19 (Supplementary Fig. 3). The isolated exosomes displayed a uniform distribution of particle sizes, with an average diameter of 125.2 nm and a concentration of 7.22 × 10^8^ particles/mL measured by nanoparticle tracking analysis (NTA) (Fig. 2b). The protein concentration of exosome was quantified by bicinchoninic acid (BCA) protein assay. Transmission electron microscopy (TEM) imaging depicted exosomes with a typical circular or cup-shaped morphology, with a diameter of approximately 100 nm (Fig. 2e and Supplementary Fig. 4).

To produce EBO, the initial step involved the preparation of ONB, which was subsequently coated with the exosome membrane. The synthesis of ONB commenced with the conjugation of BSA to dextran sulfate through a mixture followed by ultrasonication. Meanwhile, oxygen was introduced into the system throughout the ultrasonication process. The synthesis of EBO involved subjecting the exosome and ONB to ultrasonication (Fig. 2a). The mechanical forces induced by ultrasonication resulted in the transient disruption of the exosome structure, allowing for the reassembly of exosomes around the ONB, forming a core-shell structure. TEM (Fig. 2e) and scanning electron microscopy (SEM) (Supplementary Fig. 5) imaging confirmed the presence of this core-shell structure, with EBO exhibiting a bilayer core-shell morphology and possessing a diameter ranging from approximately 150 to 200 nm. The concentration of EBO was tested by NTA as shown in Supplementary Fig. 6. Dynamic light scattering (DLS) analysis revealed that ONB possessed an average hydrodynamic diameter of 122.50 nm with a mean zeta potential of −40.73 mV, while EBO exhibited an average hydrodynamic diameter of 192.02 nm with a mean zeta potential of −23.30 mV (Fig. 2c, d). The negative charges had been established to stabilize the nanobubbles generated via ultrasonic cavitation^30^. Consequently, the negatively charged EBO can effectively shield the oxygen nanobubbles from Ostwald ripening and coalescence, thereby enhancing their stability during the long-term storage, particularly in clinical applications.

The internalization of Dio-labeled EBO into HDF-a (human dermal fibroblasts, adult) cells was demonstrated through co-incubation for 6 h, as depicted in Fig. 2f. The orthogonal XZ and YZ sections of a Z-stack confirm the presence of EBOs in the cytoplasm (Fig. 2f (ii) and Supplementary Video 1). The effectiveness of the conjugation between dextran sulfate and BSA was evaluated by measuring the UV-vis absorbance at 294 nm and 420 nm, which correspond to the intensity of browning, indicative of early and late Maillard reaction products (MRP), respectively^31^. The results indicated a significant increase in absorbance at both 294 nm and 420 nm after overnight mixing, compared to non-ultrasonicated BSA (Fig. 2g). Furthermore, ultrasonication leads to a further increase in absorbance compared to the mixture alone, confirming the formation of glycosylated protein conjugates. To further validate the conjugation, sodium dodecyl sulfate–polyacrylamide gel electrophoresis (SDS-PAGE) was conducted (Fig. 2i). The monomer of BSA exhibited a molecular weight of approximately 66 kDa. After ultrasonication, a similar protein band at approximately 66 kDa was observed in the shell, ONB, and EBO samples, indicating the presence of BSA in these formulations. However, the protein band appeared to be slightly shifted compared to native BSA. This shift can be attributed to the increased molecular mass resulting from the conjugation between protein and polysaccharides induced by ultrasonication, indicating the successful conjugation. FTIR analysis was performed to identify functional groups of ONB (Fig. 2h). The absorption bands at 1650 cm^−1^ and 1542 cm^−1^ were attributed to distinctive amide І and amide II bands from proteins^32^. The spectral features of dextran appeared at approximately 1200–1000 cm^−1^ region. The intensity change observed in the spectral regions around 1650 cm^−1^ and 1542 cm^−1^ were ascribed to the modifications of C = O and C–N stretching vibrations originating from amide I and II, respectively, due to the Maillard reaction. An increase in band intensity was observed in the region of 3700–3000 cm^−1^, corresponding to the vibrations associated with N-H and O-H groups^33^. This enhancement can be attributed to the higher absorbance of MRP that was generated. The extended duration of oxygen nanobubble presence during storage is crucial for efficient oxygen release. To evaluate the stability, particularly the longevity of the core-shell structure of EBO, TEM images were obtained under different storage conditions (2 days at 37 °C and 1 month at 4 °C). As illustrated in Supplementary Fig. 7, despite the dissociated protein observed in the background after 1 month at 4 °C (possibly exosome fragments with negative staining), EBO successfully maintains its core-shell structure in both conditions. This resilience is advantageous for ensuring sustained oxygen supply upon subsequent use.

### Characteristics of EBO-Gel

**Fig. 3:**
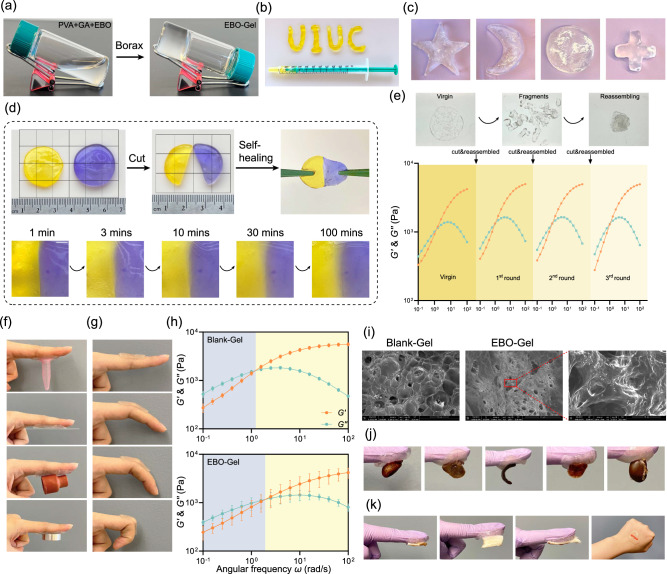
Characterizations of EBO-Gel. **a** Formation of EBO-Gel. **b** Injectability of EBO-Gel through the syringe. **c** Shape remodeling and adaptability of EBO-Gel. **d** Macroscopic self-healing property of EBO-Gel over time. Scale bar (in the 2^nd^ row): 2 mm. **e** Storage modulus (${G{{\hbox{'}}}}$) and loss modulus (${G{{\hbox{'}}}{{\hbox{'}}}}$) of EBO-Gel after cyclic cutting and reassembling process. **f** Adhesive capacity on different substrates. **g** Adhesion to finger with different bending angles. **h** Linear viscoelastic rheological characterization of Blank-Gel and EBO-Gel. The transition from the blue region to the yellow region signifies the shift from liquid-like to solid-like behavior during the gelation process. **i** SEM images of Blank-Gel and EBO-Gel. The red area indicates the magnified region. Scale bars are shown on each image respectively. **j** Adhesion to rat organs (from left to right: heart, liver, spleen, lung, and kidney). **k** Adhesion to skin tissue from different species (from left to right: rat, porcine, mouse, and human). Data are presented as mean ± SD (**h**). Representative images are shown from two independent experiments with similar results (**i**). Source data are provided as a Source Data file. PVA polyvinyl alcohol, GA gelatin, Blank-Gel hydrogel scaffold without nanoparticles, EBO-Gel EBO nanoparticles-embedded hydrogel.

The aqueous mixture of PVA, gelatin and EBO is a low viscosity liquid (Supplementary Video 2), upon the addition of 2 wt% of borax, transient cross-links rapidly form boronic ester bonds, creating a viscoelastic hydrogel within seconds. Figure 3a, b, and Supplementary Fig. 10 show evidence of these key rheological features. Known as protorheology, these images can be used for both qualitative and quantitative inference (details in Methods). For example, the tilted vial of EBO-Gel in Fig. 3a is evidence of a sufficiently high viscosity to inhibit flow on the observation timescale of 5 min, with an implied viscosity on the order of *η* ~ 10^4 ^Pa·s at this gravitational stress of ~100 Pa. At higher applied stress, the EBO-Gel readily flows, as evidenced in Fig. 3b with the material extruded by hand from a syringe with characteristic flow stress ~ 100,000 Pa. This is evidence for dramatic shear thinning, since the material readily flows at high stress, but retains its shape at lower stress (in the form of UIUC in Fig. 3b). This injectable design further facilitates conformity with the shape of a defect, enabling precise and tailored application.

SEM images reveal the porous structure and the presence of nanoscale particle in EBO-Gel (Fig. 3i). In contrast to the smooth surface observed in Blank-Gel (Supplementary Fig. 8), EBO-Gel exhibits a rougher surface with nanoscale particles adhering to the gel skeleton, indicating the existence of EBO. The EBO release profile was shown in Supplementary Fig. 9, indicating that over 80% of EBO is released from EBO-Gel within 48 h. This corresponds with the intervals for refreshing wound dressings, ensuring the efficient release of nanoparticles to the wound bed before the next dressing. To ascertain the shape adaptability, the hydrogel underwent iterative remodeling, wherein it was repeatedly reconfigured into various morphologies including star shape, crescent moon shape, round, and cross shape, which demonstrates the favorable capacity of EBO-Gel to conform effectively to irregular wound shapes, thus highlighting its adaptability in a clinical setting (Fig. 3c).

Self-healing ability is an additional crucial factor that influences the wound adaptability of hydrogel dressings^34–36^. The mechanism that is responsible for the self-healing ability of our PVA-GA hydrogels is the transient boronic crosslinks. Two possible routes were presumed, intra- (PVA-PVA) and interspecies (PVA-GA) crosslinks: (i) boronic-esters; and (ii) ionic crosslinks. We examined self-healing properties at both macro and micro levels. At the macroscopic level, two EBO-Gel specimens, each stained with a distinct color (purple and yellow), were brought together. In this protorheology demonstration, the reassembled gel was subjected to stretching after a minute to evaluate its healing capacity. Notably, the dyes exhibited rapid migration along the damaged interface, which could be visually observed from 3 to 100 min (Fig. 3d). To explore the self-healing characteristic, an evaluation of the gel linear viscoelasticity was assessed further carefully following a cyclic process of cutting and reassembling (Fig. 3e). The findings revealed that even after undergoing three cycles of cutting and reassembling, the hydrogels could consistently maintain their linear viscoelastic properties, i.e., ${G{{\hbox{'}}}}$ and ${G}{{{{\hbox{'}}}{{\hbox{'}}}}}$. We further confirm the recoverability with the alternating strain amplitude test, i.e., application of small-large-small strain amplitude during oscillatory shear. The results show almost full recovery, where the unrecoverable part is attributable to gelatin bundle at $T \, < \, {T}_{g}\, \approx \, 60^\circ {{{{{\rm{C}}}}}}$ (Supplementary Fig. 13). The results from these rheological experiments suggest that both blank- and EBO-Gels can self-heal and retain their rheological properties even after being strained, which is favorable for clinical wound care.

Adhesion properties were investigated on materials and tissues. The adhesive properties of the hydrogel on diverse substrates are shown in Fig. 3f, including plastic, glass, rubber, and steel. Linear viscoelastic characterization with oscillatory shear frequency sweep experiments, Fig. 3h, show that our hydrogels are viscoelastic fluids with a relaxation time based on crossover frequency $\lambda=1/{\omega }_{c}\approx 1.5{{{{{\rm{s}}}}}}$, a large zero shear viscosity ~2 kPa·s, and a plateau elastic modulus ~5 kPa. This linear viscoelastic behavior rationalizes why the materials appear solid-like with minimal flow, but the gels can also remodel, making them suitable for irregularly shaped and bleeding wounds and suitable for gluing. We also show that the hydrogel exhibited remarkable fixation on the finger, retaining its adhesion when the fingers were flexed within a range of 0° to 90°, providing further evidence of exceptional adaptability of EBO-Gel, particularly on body parts that exhibit a wide range of motion (Fig. 3g). Additionally, the EBO-Gel was able to tightly adhere to the surfaces of rat organs and effectively supported the self-weight of these organs (Fig. 3j). It also showed strong adhesion on skin surfaces from various species (Fig. 3k). The adhesion can be attributed to the formation of strong hydrogen bonds between the skin tissue and EBO-Gel. This robust adhesive feature establishes the EBO-Gel as a reliable physical sealing agent for hemostasis purposes. Effective degradation is crucial to prevent the retention of residual hydrogel in deep wounds, that could impede the healing due to its strong adhesion and tissue adaptability. In vitro degradation analysis revealed that EBO-Gel can degrade by 80% within 3 days under simulated physiological conditions (Supplementary Fig. 14), ensuring compatibility with the subsequent healing process and alignment with the dressing change intervals in in vivo studies.

### Oxygen supply and ROS elimination assessment

**Fig. 4:**
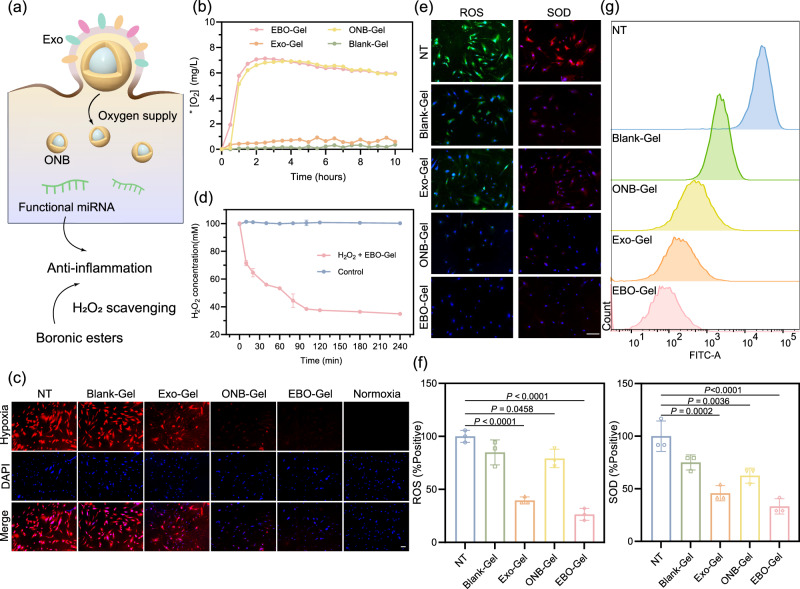
Oxygen supply and antioxidant properties of EBO-Gel. **a** Illustration of oxygen supply and anti-inflammation mechanisms offered by EBO-Gel. **b** Oxygen release curve monitored within 10 h. **c** Intracellular hypoxia conditions of different treatments. Scale bar: 50 μm. **d** H_2_O_2_ consumption capacity of EBO-Gel ($n$  =  3 independent samples). **e** ROS/SOD detection in HDF-a cells. Scale bar: 100 μm. **f** Quantification of ROS/SOD fluorescence ($n$  =  3 biologically independent samples). **g** Evaluation of H_2_DCFDA signal after treated with Blank-Gel, ONB-Gel, Exo-Gel, and EBO-Gel. Data are presented as mean ± SD (**d**, **f**). Statistical analysis was performed by one-way ANOVA with Tukey’s multiple comparisons (**f**). Representative images are shown from three independent experiments with similar results (**c**, **e**). Source data are provided as a Source Data file. Exo adipose-derived stem cell (ADSC)-derived exosomes, ONB oxygen nanobubble, NT no treatments, Blank-Gel hydrogel scaffold without nanoparticles, Exo-Gel adipose-derived stem cell (ADSC)-derived exosomes-embedded hydrogel, ONB-Gel oxygen nanobubble-embedded hydrogel, EBO-Gel EBO nanoparticles-embedded hydrogel.

To verify the oxygen release properties, oxygen concentration was monitored within 10 h. Figure 4b demonstrates the oxygen-supplying capabilities of both EBO-Gel and ONB-Gel in hypoxic conditions, in comparison to Exo-Gel and Blank-Gel. Extended oxygen release profiles were compared with a Control-Gel formed without ultrasonication. Despite a decline after 12 h, the dissolved oxygen in the hypoxic environment exceeded that in Control-Gel by 40 h (Supplementary Fig. 15). This supports our treatment of changing dressings every 2 days in subsequent in vivo experiments to sustain optimal oxygen levels in the wound bed. Subsequently, hypoxia in human dermal fibroblast (HDF-a) cells was investigated by employing [Ru(dpp)_3_]Cl_2_ (RDPP) as a cellular hypoxia indicator (Fig. 4c). Remarkably, cells incubated with EBO-Gel and ONB-Gel exhibited the low RDPP fluorescence intensity, signifying effective hypoxia mitigation, which serves to validate the exceptional cellular oxygen supply capability of EBO-Gel.

ROS scavenging properties can be ascribed to two primary factors: (1) the presence of boronic ester bonds in the gel enables it to respond to H_2_O_2_ and facilitate its decomposition; (2) the exosomes derived from ADSCs have been demonstrated to possess antioxidant properties^37^ (Fig. 4a). Figure 4d showed a rapid reduction in H_2_O_2_ concentration within the first 30 min of incubation with the gel, with a continued decrease observed over the course of 240 min, indicating the ability of the gel to effectively scavenge H_2_O_2_. ROS/SOD fluorescence detection on HDF-a cells further demonstrated the ROS elimination capacities of EBO-Gel (Fig. 4e, f). Furthermore, flow cytometry analysis showed EBO-Gel-treated cells exhibited weak H_2_DCFDA fluorescence (Fig. 4g).

### Exosome delivery enhancement study

**Fig. 5:**
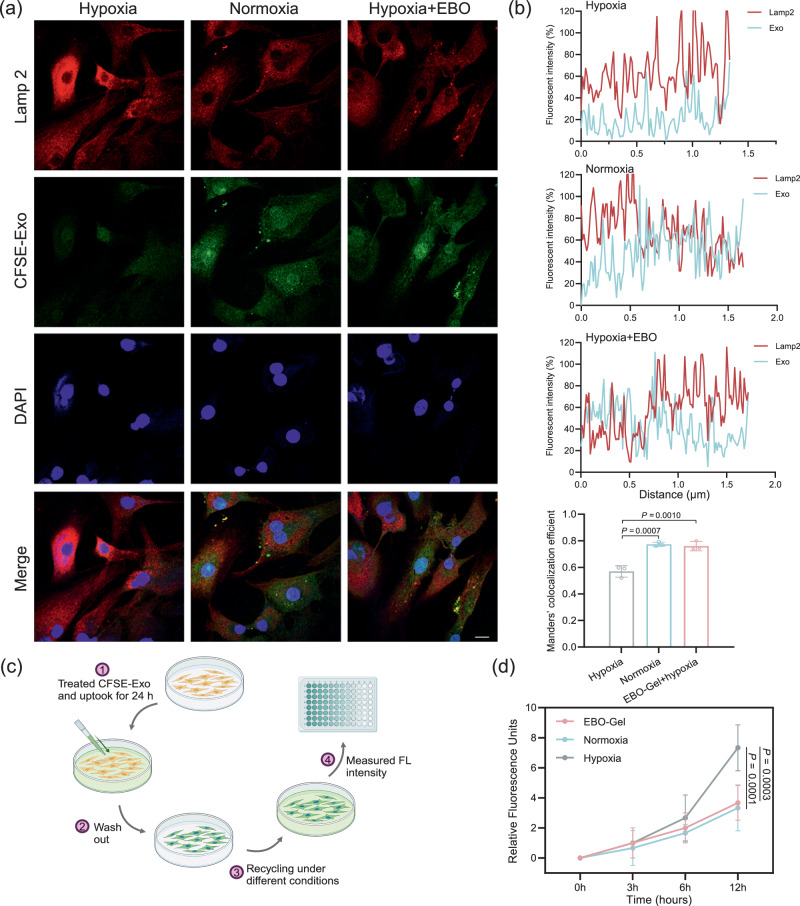
Enhanced intracellular exosome delivery. **a** Representative immunofluorescent images of CFSE-Exo and Lamp2 under different treatments. Scale bar: 10 μm. **b** Fluorescence intensity and colocalization efficiency of different treatments ($n$  =  3 biologically independent samples). **c** Illustrations of the procedures for the evaluation of exosome recycling. **d** Quantification of exosome recycling in the culture medium ($n$  =  3 biologically independent samples). Data are presented as mean ± SD (**b**, **d**). Statistical analysis was performed by one-way ANOVA with Tukey’s multiple comparisons (**b**) or two-way ANOVA with Dunnett’s multiple comparisons (**d**). Representative images are shown from two independent experiments with similar results (**a**). Source data are provided as a Source Data file. EBO-Gel EBO nanoparticles-embedded hydrogel.

Oxygen sensing pathway was found to regulate endocytosis^38^. Previous studies have also demonstrated that intracellular cargo delivery of exosomes is undermined in hypoxic tissues^18^. Therefore, EBO is expected to restore exosome delivery efficiency in hypoxic microenvironments by supplying oxygen. To explore the exosome delivery efficiency, HDF-a cells were co-cultured with CFSE-labeled exosomes for a duration of 8 h. Immunofluorescence was performed to assess the colocalization between exosomes and Lamp2, a marker of endolysosomes (Fig. 5a). Remarkably, under hypoxic conditions, a notable decrease in the colocalization of exosomes and Lamp2 was observed, indicating a diminished transfer of exosomes to the endolysosomes, which typically serve as sites for the release of protein cargo into the cytoplasm (Fig. 5b).

Hypoxia has been known to impact endocytic recycling via the modulation of Rab11^19,39,40^. Consequently, the recycling of exosomes through exocytosis may hinder the efficient release of their cargo into the cellular cytoplasm, thus compromising the delivery of therapeutic cargo. To prove this hypothesis, a period of 24-h of internalization of CFSE-labeled ADSC-derived exosome was allowed, followed by the assessment of CFSE signals that were subsequently re-secreted into the culture medium over a 12-h interval under distinct experimental conditions, including hypoxia, normoxia, and treatment with an oxygen-enhancing agent (EBO) (Fig. 5c). The obtained results revealed a heightened presence of exosome proteins recycled to the culture medium during hypoxic incubation in contrast with both EBO-treated and normoxic groups (Fig. 5d). This observation signifies the potential of EBO treatment to augment the evasion of exosomes from enhanced endocytic recycling caused by hypoxia, thereby facilitating the therapeutic cargo release within the cellular cytoplasm.

### Evaluation of biocompatibility and hemostatic efficacy

**Fig. 6:**
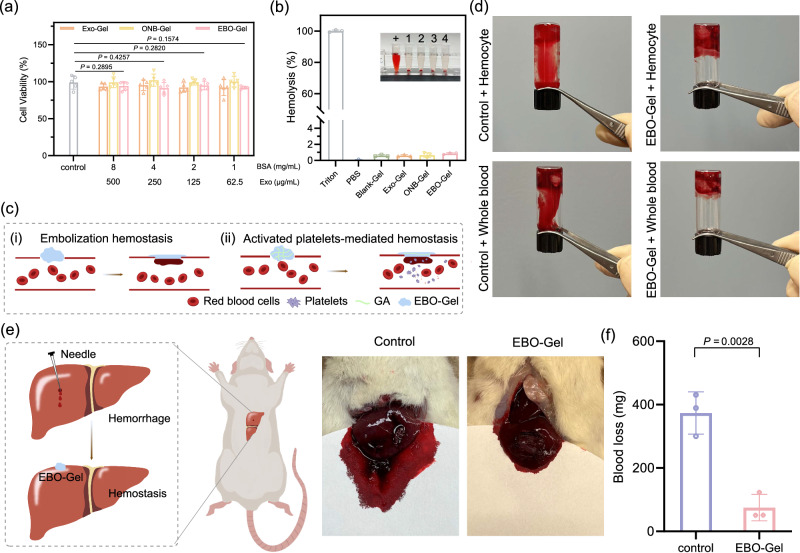
Biocompatibility and hemostatic properties assessment. **a** Cell viability of HDF-a cells incubated with different concentrations of Exo-Gel, ONB-Gel, and EBO-Gel ($n$  =  5 biologically independent samples). **b** Blood compatibility evaluated by hemolysis assay ($n$  =  3 biologically independent samples). Inserted image: +: Positive control (Triton); 1: Blank-Gel; 2: Exo-Gel; 3: ONB-Gel; 4: EBO-Gel. **c** Mechanisms of hemostasis capacity: (i) Embolization hemostasis offered by remodeling, adhesive, and self-healing properties of EBO-Gel; (ii) Activated platelet-mediated hemostasis offered by GA. **d** In vitro procoagulant effects of EBO-Gel. **e** Illustration (Left) and Digital photos (Right) of hemostasis evaluation on rat liver hemorrhage model. **f** Quantitation of blood loss in **e** ($n$  =  3 biologically independent experiments). Data are presented as mean ± SD (**a**, **b**, **f**). Statistical analysis was performed by two-way ANOVA with Dunnett’s multiple comparisons (**a**) or two-tailed Student’s $t$ test (**f**). Representative images are shown from three independent experiments with similar results (**b**, **d**, **e**). Source data are provided as a Source Data file. GA gelatin, Exo-Gel adipose-derived stem cell (ADSC)-derived exosomes-embedded hydrogel, ONB-Gel oxygen nanobubble-embedded hydrogel, EBO-Gel EBO nanoparticles-embedded hydrogel.

To examine the biocompatibility of EBO-Gel, cytotoxicity test and hemolysis assay was conducted. For cytotoxicity experiments, HDF-a cells were incubated with four different concentrations of Exo-Gel, ONB-Gel, and EBO-Gel for 24 h. As shown in Fig. 6a, all groups performed high cell viability, surpassing 80%, which indicates their excellent biocompatibility. For hemolysis assay, all hydrogel groups demonstrated a remarkable blood compatibility, as evidenced by a hemolysis ratio of less than 2% (Fig. 6b).

Hemostasis plays a pivotal role in the initial phase of wound healing, serving as a critical step that establishes a temporary barrier to safeguard the underlying tissues against additional harm and potential infection^8^. Given the frequent occurrence of bleeding in both surgical wounds and traumatic injuries, advanced wound dressings should possess the ability to expedite the hemostasis process. Gelatin has gained significant attention to be a promising hemostasis material due to its similarity in composition to the extracellular matrix, enabling gelatin to effectively trigger platelet aggregation and facilitate hemostasis^41,42^. In addition, PVA hydrogel as a self-healing bioadhesive hydrogel offers a promising application as an embolic hemostatic agent, allowing it to seamlessly adhere to the site of bleeding, forming a physical seal that effectively controls and manages the bleeding process (Fig. 6c). Therefore, the hemostatic function of EBO-Gel was assessed both in vitro and in vivo. EBO-Gel, when mixed with rat whole blood and blood cells at a volume ratio of 2:1 for 1 min, showed coagulation. This underscores its hemostatic capability, similar to commercial products including CURAD® BloodStop® Hemostatic Gauze and BleedStop™, in comparison to both the control group and a non-hemostatic gel (Carbopol hydrogel group) (Fig. 6d and Supplementary Fig. 16). In the rat liver hemorrhage model, the bleeding site was promptly sealed upon application of EBO-Gel, leading to a substantial reduction in blood loss, aligned with the in vitro findings (Fig. 6e, f, Supplementary Figs. 17, 18). Our results highlight the effective hemostatic performance of EBO-Gel, in addition to wound healing characteristics.

### In vitro facilitation of proliferation, migration, and angiogenesis

**Fig. 7:**
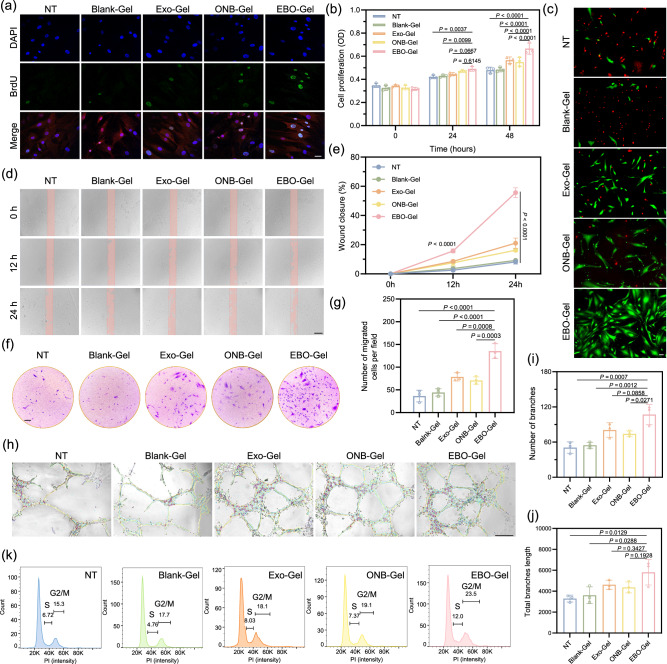
Evaluation of enhanced cell proliferation, migration, and angiogenesis. **a** Immunofluorescence images of BrdU staining in HDF-a cells. Scale bar: 50 μm. **b** Cell proliferation assay of HDF-a cells with different treatments ($n$  =  3 biologically independent samples). **c** Calcein AM/PI fluorescence staining to examine live/dead cells. Scale bar: 50 μm. **d** Scratch wound healing assay conducted on HDF-a cells with different treatments subjected to 0 h, 12 h, or 24 h of hypoxia. Scale bar: 200 μm. **e** In vitro wound closure rate followed by scratching assay in **d** ($n$  =  3 biologically independent samples). **f**, **g** Brightfield images and quantitative result of transwell migration of HDF-a cells ($n$  =  3 biologically independent samples). Scale bar: 200 μm. **h** Tube formation ability of HUVECs with different treatments. Scale bar: 200 μm. Quantitative results of (**i**) number of branches and (**j**) total branches length ($n$  =  3 biologically independent samples in **i** and **j**. **k** Cell cycle analysis of HDF-a cells with different treatments. Data are presented as mean ± SD (**b**, **e**, **g**, **I**, **j**). Statistical analysis was performed by two-way ANOVA with Dunnett’s multiple comparisons (**b**, **e**), one-way ANOVA with Tukey’s multiple comparisons (**g**, **i**, **j**). Representative images are shown from two (**a**, **c**) or three (**d**, **f**, **h**) independent experiments with similar results. Source data are provided as a Source Data file. NT no treatments, Blank-Gel hydrogel scaffold without nanoparticles, Exo-Gel adipose-derived stem cell (ADSC)-derived exosomes-embedded hydrogel, ONB-Gel oxygen nanobubble-embedded hydrogel, EBO-Gel EBO nanoparticles-embedded hydrogel.

Cell proliferation was assessed using HDF-a cells, which were exposed to hypoxic conditions for 24 and 48 h after treatment with hydrogels. Notably, the EBO-Gel group exhibited a higher number of proliferating cells in comparison to the other groups, as demonstrated in Fig. 7b, c. Furthermore, real-time monitoring of cell proliferation was conducted using a BrdU incorporation assay and cell cycle analysis. The EBO-Gel group displayed a significantly higher positive signal of BrdU (Fig. 7a) and more cells in S/(G2/M) phases compared to the other groups (Fig. 7k), indicating an increase in the newly synthesized DNA, which corroborates the ability of EBO-Gel in promoting cell proliferation.

ADSC-Exo plays a crucial role in promoting dermal fibroblast migration and wound healing by releasing MALAT1, a specific long non-coding RNA^43^. Additionally, the modulation of Apoptosis Peptidase Activating Factor 1 (APAF1) through miR-93-3p in ADSC-Exos contributes to improved cellular viability and migration, particularly in hypoxic conditions^44^. The migration ability of HDF-a cells under hypoxic conditions was assessed using both scratching assay and transwell migration test. In the scratching assay, cell migration was visually monitored at 0, 12, and 24 h. Remarkably, the EBO-Gel treatment group displayed a higher wound closure rate in comparison to the other groups, indicating enhanced cell migration (Fig. 7d, e). Similarly, the EBO-Gel group exhibited a higher number of migrated cells in the transwell after 24 h of co-incubation (Fig. 7f, g). These results collectively demonstrate the capacity of the EBO-Gel to promote fibroblast migration.

Angiogenesis, a pivotal process in wound healing, entails the formation of tube-like structures by endothelial cells, which progressively extend, branch, and establish interconnected networks^45^. The establishment of an extracellular matrix (ECM) is vital for neovascularization, with collagen deposition playing a fundamental role in aortic endothelial cell migration, which is an oxygen-dependent process^46^. Hence, oxygen supply might be a potential strategy for enhancing blood vessel formation. Moreover, EBO aids angiogenesis through exosomes containing regulators such as Nrf2 and FGF2, with exosomes promoting β-catenin activation and harboring microRNAs (miR-31, miR-125a) that modulate angiogenic pathways, collectively enhancing proangiogenic effects^47,48^. In vitro tube formation capacity was evaluated using HUVECs (Fig. 7h). After 6 h of co-incubation, the EBO-Gel-treated group developed a higher number of branches (Fig. 7i) and the maximum total branch length (Fig. 7j), indicating the potential of EBO-Gel to facilitate angiogenesis.

### In vivo wound healing analysis

**Fig. 8:**
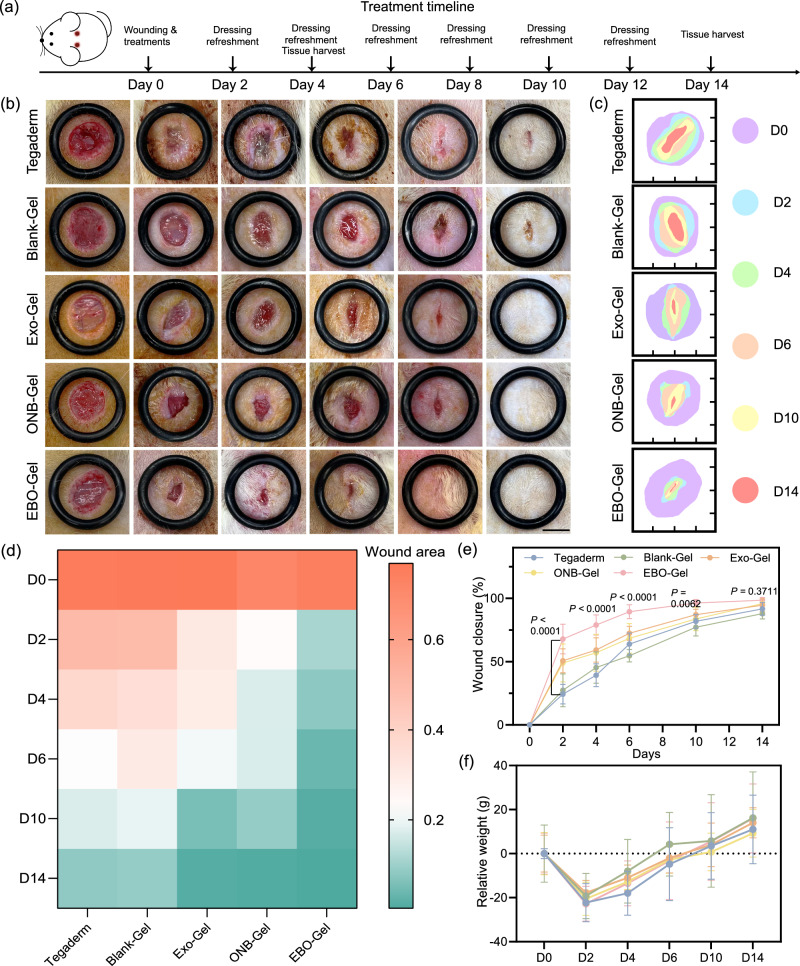
In vivo efficacy of EBO-Gel in a rat full-thickness wound model. **a** Illustration of wound creation and treatment timeline. **b** Representative digital photos of wounds with varied treatments at different time points. Scale bar: 7 mm. **c** Traces of wound closure from day 0 to day 14. Quantitative analysis of (**d**) wound area (cm^2^) and (**e**) wound closure rate ($n$  =  6 biologically independent wounds) over time. **f** Relative body weight of rats after varied treatments ($n$  =  4 animals per group). Data are presented as mean ± SD (**e**, **f**). Statistical analysis was performed by two-way ANOVA with Dunnett’s multiple comparisons (**e**). Source data are provided as a Source Data file. Tegaderm no treatments, Blank-Gel hydrogel scaffold without nanoparticles, Exo-Gel adipose-derived stem cell (ADSC)-derived exosomes-embedded hydrogel, ONB-Gel oxygen nanobubble-embedded hydrogel, EBO-Gel EBO nanoparticles-embedded hydrogel, D0-D14 days post-surgery.

EBO-Gel showed robust tissue adhesion, oxygen release, antioxidant, and enhanced exosome delivery properties, all of which are expected to be promising for noninvasive wound closure. Therefore, the wound healing performance of the EBO-Gel was assessed in a rat full-thickness defect model. Figure 8a depicts the surgical procedure, dressing application, and healing timeline. Results show that the EBO-Gel-treated group exhibited a notably accelerated healing rate compared to the other groups, particularly during the proliferation stage from day 2 to day 10 (Fig. 8b–e). Remarkably, the Tegaderm group exhibited signs of infection and inflammation on days 2 and 4, while the groups treated with hydrogel dressings showed no significant infection (Fig. 8b). Throughout the treatment duration, a reduction in body weight was observed during the initial two days post-surgery, which can be attributed to the recovery from anesthesia. Subsequently, the animals exhibited a gradual weight gain, with an increase in weight observed by day 14 (Fig. 8f). Additionally, after 14 days of treatment, the wounds treated with the EBO-Gel displayed excellent recovery quality, characterized by the absence of hypertrophic and keloid scars.

**Fig. 9:**
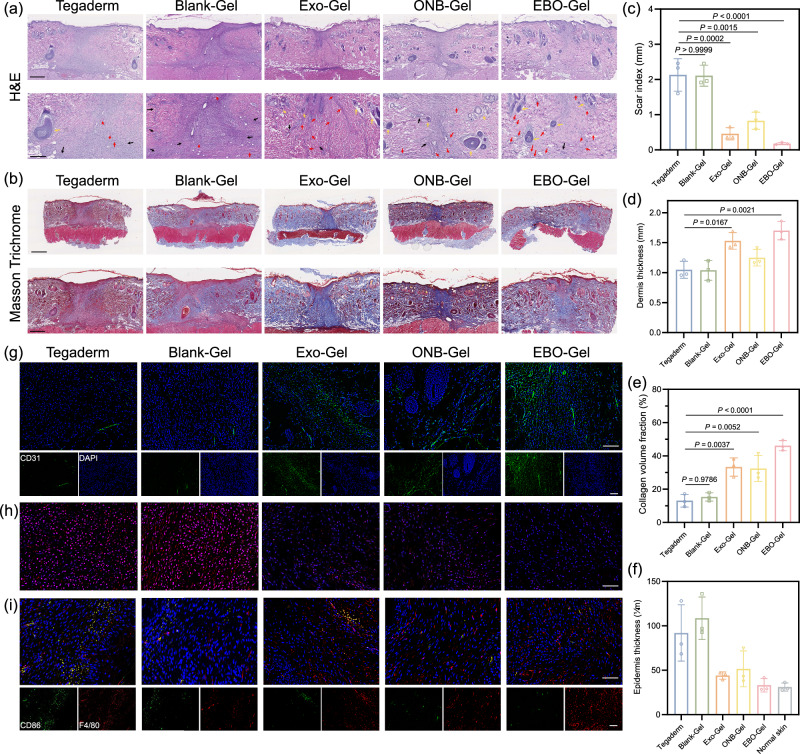
histological analysis of wounds that underwent treatments with different hydrogels. Representative images (top) and magnified images (bottom) of (**a**) H&E and (**b**) Masson’s trichrome staining of the wound tissue on Day 14. Red arrows: new blood vessel formation; Black arrows: inflammation area; Yellow arrows: newly generated-hair follicles. Scale bar: 500 μm (**a**) and 1 mm (**b**) for the normal-sized image; 250 μm (**a**) and 500 μm (**b**) for the magnified image. Various parameters for wound healing evaluation, including: (**c**) Scar index; (**d**) Dermis thickness; (**e**) Collagen volume fraction; and (**f**) Epidermis thickness ($n$  =  3 biologically independent samples in **c**–**f**). Representative fluorescence images of (**g**) CD31 immunostaining, (**h**) DHE (red) staining, and (**i**) CD86 and F4/80 immunostaining of wound tissues on Day 14 post-treatment. Cell nuclei were stained with DAPI (blue). Scale bar: 100 μm (CD31) and 50 μm (DHE and CD86). Data in are presented as mean ± SD (**c**–**f**). Statistical analysis was performed by one-way ANOVA with Tukey’s multiple comparisons (**c**–**f**). Representative images are shown from three independent experiments with similar results (**a**, **b**, **g**, **h**, **i**). Source data are provided as a Source Data file. Tegaderm no treatments, Blank-Gel hydrogel scaffold without nanoparticles, Exo-Gel adipose-derived stem cell (ADSC)-derived exosomes-embedded hydrogel, ONB-Gel oxygen nanobubble-embedded hydrogel, EBO-Gel EBO nanoparticles-embedded hydrogel.

To comprehensively assess the therapeutic effectiveness of the different treatments, tissue samples collected on Day 4 and Day 14 were subjected to histopathological evaluation using hematoxylin and eosin (H&E) staining and Masson’s trichrome staining, focusing on skin repair parameters including scar index, dermis thickness, epidermis thickness, and collagen fraction^49,50^. Day 4 represents a crucial time window for the inflammation and proliferation stages of the healing process, while Day 14 can reflect the regeneration stage and healing efficacy. As illustrated in Supplementary Figs. 19 and 20, the EBO-Gel group exhibited a consistently regenerated epidermis, increased fibroblast proliferation, enhanced angiogenesis, and collagen fiber formation. In contrast, wounds in the Tegaderm and Blank-Gel groups showed incomplete closure and higher areas of inflammation infiltration. This highlights the role of EBO-Gel in influencing the inflammation, proliferation, and early regeneration stages of healing. The staining results indicated visible wound closure and the development of newly formed epidermis across all treatment groups on Day 14 (Fig. 9a, b). However, the EBO-Gel treated group exhibited distinct characteristics with flatter wound surfaces, demonstrating a continuous and coherent epidermis, highlighting its significant potential in preventing keloid formation and achieving scarless wound healing. Furthermore, the EBO-Gel group displayed a higher density of newly formed blood vessels and reduced inflammation areas, signifying its capability to enhance angiogenesis and possess antioxidant properties. The increased count of newly generated hair follicles and glands (Supplementary Figs. 21 and 22), and the organized formation of granulation tissue further underscored the superior quality of healing achieved through EBO-Gel treatment.

The Scar Index (SI), representing scar size, is determined by dividing the scar area (mm²) by the corresponding average dermal thickness (mm). The calculated results showed that the group treated with EBO-Gel obtained the lowest scar index, indicating a more favorable healing outcome (Fig. 9c). Complete wound healing is also marked by the repair of dermis. Dermal thickness measurements revealed that the group treated with EBO-Gel exhibited a thicker dermal layer, indicating a more robust healing process and well-repaired tissue (Fig. 9d). Conversely, the thickness of the newly formed epidermis was observed to be closer to that of normal skin tissue, indicating a more balanced healing response (Fig. 9f). This thinner epidermis is advantageous in preventing the formation of thick scabs or keloids, which can otherwise impair the normal functioning of the regenerated tissue. Furthermore, Masson’s trichrome staining demonstrated collagen deposition and organization in various treatments. The EBO-Gel treated group showed an abundance of densely packed collagen fibers in magnified regions. Notably, these fibers demonstrated a more mature phenotype characterized by well-organized alignment and an intricate collagen network topology, especially in comparison to the Tegaderm and Blank-Gel group (Fig. 9e). The quantitative assessment of collagen volume fraction aligned with the observed findings, which verified the improved collagen deposition ability of EBO-Gel.

To assess the multifaceted impact of EBO-Gel on angiogenesis and anti-inflammatory responses in vivo, we performed fluorescent staining of wound tissues on Day 14 post-treatment. The immunofluorescent staining for CD31, a marker indicating angiogenesis, exhibited heightened expression and fluorescence intensity in the EBO-Gel treated group, showcasing its potential in promoting angiogenesis (Fig. 9g and Supplementary Fig. 23). By incorporating ADSC-exosomes, EBO-Gel has the potential to mitigate excessive inflammation, forestalling the development of dysfunctional scars throughout the healing process. Our examination encompassed ROS levels, inflammatory cytokine expression, and macrophage phenotypes. Dihydroethidium (DHE) staining unveiled significantly lower ROS levels in wounds treated with EBO-Gel in comparison to the Tegaderm control and Blank-Gel (Fig. 9h and Supplementary Fig. 23). Notably, the heightened DHE intensity in the Tegaderm control and Blank-Gel groups suggests prolonged elevated ROS levels in healed tissue even after 14 days of treatment, possibly contributing to hypertrophic scar formation. In the evaluation of immune cells through immunofluorescent staining for CD86 and CD206 with F4/80—markers for M1 inflammatory and M2 anti-inflammatory macrophages, respectively—EBO-Gel demonstrated lower CD86 and higher CD206 expression compared to control groups (Fig. 9i, Supplementary Figs. 23–25). Furthermore, the immunofluorescent staining of interleukin-6 (IL-6), an inflammatory marker, exhibited the lowest expression in the EBO-Gel group, solidifying its efficacious anti-inflammatory effects (Supplementary Figs. 26 and 27).

Effective wound closure could be potentially hindered if the adaptive hydrogel fails to degrade adequately in deep wounds, underscoring the crucial importance of its degradability^51^. For in vivo degradation assessment, the visual observation of EBO-Gel reveals a progressive reduction in size and complete degradation within a 3-day period (Supplementary Fig. 28). H&E staining of tissues surrounding the injection sites on Day 3 post-injection revealed no discernible inflammation induced by the injection compared to normal skin and subcutis tissues (Supplementary Fig. 29). This suggests the in vivo biocompatibility of EBO-Gel. Autolysis characterizes the degradation of EBO-Gel, where water molecules, with unshared electron pairs, initiate the hydrolysis of boron atoms^51,52^. This hydrolysis causes the gradual breakdown of the borax bond crosslinked network, weakening both borax and hydrogen bond crosslinks. Consequently, the hydrogel network progressively loosens when exposed to a water environment over time, ultimately resulting in the complete dissolution of the hydrogel. To comprehensively assess the biosafety of EBO-Gel, we conducted H&E staining on visceral slices obtained from animals treated for a 14-day period (Supplementary Fig. 30). The results revealed no obvious systemic toxicity in animals treated with EBO-Gel compared to untreated animals, validating the biosafety of EBO-Gel.

## Discussion

The healing of traumatic and surgical wounds represents a significant challenge that affects millions of individuals worldwide, placing immense strain on healthcare systems and patients alike. Among the most common complications encountered in such wounds are bleeding, poor healing, and the development of hypertrophic scarring. Adipose-derived stem cell-derived exosomes have emerged as promising therapeutic agents, boasting antioxidant and pro-angiogenic properties that promote tissue regeneration. However, the hypoxic microenvironment of poorly healing wounds can compromise their delivery efficacy.

Although acute hypoxia can stimulate initial tissue regeneration by increasing vascular endothelial growth factor (VEGF), prolonged hypoxia hinders optimal recovery^53^. While hypoxia initiates neovascularization, it cannot sustain the process^54^, emphasizing the importance of restoring oxygen levels after the initial acute hypoxia phase in wound healing. Moreover, lower oxygen tension during hypertrophic scar progression suggests oxygen supply as a potential strategy to expedite wound healing and prevent hypertrophic scar formation^55^. Addressing these multifaceted challenges, our study introduces a tissue adhesive PVA/GA hybrid hydrogel that exhibits a remarkable hemostatic synergistic effect by incorporating the therapeutic properties of gelatin, specifically targeting irregularly shaped and bleeding wounds. Moreover, we have taken a pioneering step by embedding exosome-coated oxygen nanobubbles within the hydrogel, thereby elevating the delivery efficiency of exosomes and addressing hypoxia. This combination not only accelerates the wound healing process but also effectively removes excess ROS from the wound tissue, alleviating hypoxia and mitigating the risk of hypertrophic scars. The scarless healing potential of EBO-Gel is attributed to the multifaceted functions of ADSC-Exos, enriched with microRNAs such as miR-192-5p and miR-29a that regulate fibrotic responses and keloid fibroblast migration^56^. Moreover, EBO-Gel shows promise in addressing abnormal metabolic conditions and oxygen tension in hypoxic hypertrophic scars^57,58^, offering a promising avenue for further investigation in scar management.

In summary, our research has culminated in the development of a hybrid hydrogel that possesses remarkable capabilities in hemostasis, anti-inflammation, hypoxia mitigation, and enhanced exosome delivery, which, when applied to full-thickness wounds, demonstrated an accelerated healing speed and significantly improved healing quality. Notably, EBO-Gel could also be potentially used for diabetic chronic wound treatment, leveraging its oxygen-supplying capability, antioxidant properties, promotion of cell migration, and enhancement of vascularization. Future research can target other ischemic conditions, including chronic wounds and potentially cancer.

## Methods

### Ethical statement

This study complies with all relevant ethical regulations. All animal-related procedures were conducted in accordance with the guidelines of the Institutional Animal Care and Use Committee and the Division of Animal Resources at the University of Illinois under the protocol approved by the Institutional Animal Care and Use Committee at the University of Illinois (IACUC Protocol#: 23012).

### Materials

Bovine serum albumin, dextran sulfate sodium salt (Mw 200 kDa), poly(vinyl alcohol) (Mw 89–98 kDa), sodium tetraborate decahydrate, collagenase type I, 5-Bromo-2′-deoxyuridine were purchased from Sigma-Aldrich. MTT (Catalog #M6494), CFSE (Catalog #C34554), Dio (Catalog #V22886), LAMP2 monoclonal antibody (Catalog #MA1-205), H_2_DCFDA (Catalog #D399), propidium iodide, BrDU primary antibody (Catalog #B35128), Alexa Fluor™ Plus 555 Phalloidin (Catalog #A30106), CD86 primary antibody (Catalog #PA5-114995), CD206 primary antibody (Catalog #MA5-44409), F4/80 monoclonal antibody (Catalog #14-4801-82),CD31 primary antibody (Catalog #MA1-26196), IL-6 primary antibody (Catalog #MA545069), Dihydroethidium (Catalog #D1168) were obtained from Thermo Fisher Scientific. Secondary antibodies used in this study were obtained from Thermo Fisher Scientific, including Goat anti-Mouse IgG (H + L) Cross-Adsorbed Secondary Antibody, FITC (Catalog #F-2761), Goat anti-Rabbit IgG (H + L) Secondary Antibody, FITC (Catalog #65-6111), and Goat anti-Rat IgG (H + L) Cross-Adsorbed Secondary Antibody, Alexa Fluor™ 555 (Catalog #A-21434). All antibodies were diluted 100 times except Goat anti-Rabbit IgG (H + L) Secondary Antibody (1:50). ROS/Superoxide detection assay kit (ab139476), calcein AM (ab141420), DAPI (ab228549) were purchased from Abcam. Human mesenchymal stem cell marker antibody panel was purchased from R&D systems (Catalog #SC017).

### Cell lines and animals

The HDFa cell line was purchased from American Type Culture Collection (PCS-201-012, ATCC) and cells were cultured in Fibroblast Basal Medium (PCS-201-013, ATCC) supplemented with the Fibroblast Growth Kit-Low Serum (PCS-201-041, ATCC), 1× Antimycotic–Antibiotic (15240096, Thermo Fisher) at 37 °C, 5% CO_2_. HUVECs (Lonza C2517A, used experimentally before passage 6) were maintained in Endothelial Cell Growth Medium 2 (EGM) EBMTM-2 Basal Medium (Catalog #CC-3156, Lonza) supplemented with EGMTM-2 SingleQuotsTM Supplements (Catalog #CC-4176, Lonza), 1× Antimycotic–Antibiotic (15240096, Thermo Fisher) at 37 °C, 5% CO_2_. Primary ADSCs were derived from discarded donor adipose tissue, as described below. Male Sprague-Dawley rats were purchased from Envigo Laboratory (Indianapolis, IN, USA). All rats were housed at a constant temperature and humidity in a room with an artificial 12 hrs light/dark cycle and allowed free access to food and water. All animal studies were performed in accordance with the guidelines of the Institutional Animal Care and Use Committee and the Division of Animal Resources at the University of Illinois.

### ADSCs isolation, culture, and characterization

ADSCs were isolated from human adipose tissue obtained from The Specimen Procurement Service Center, Carle Health, Carle Foundation Hospital. The tissues utilized in this study are discarded “de-identified tissues” with no personal information and are exempt from the need for formal Institutional Review Board (IRB) approval and informed consent. They do not fall under Human Subjects. Briefly, tissue was digested in 0.075% collagenase type 1 prepared in PBS containing 2% penicillin/streptomycin for 30 min at 37 °C, 5% CO_2_, and the mixture was centrifuged at 2000 rpm for 5 min. Then, the cell pellet was resuspended in 3 mL of DMEM supplemented with 20% FBS, 1% L-glutamine, and 1% penicillin/streptomycin, and the cell suspension was then filtered through 70 μm cell strainer. The cells were plated in a lysine coated tissue culture plate and adhered for 48 h. After the confluency reached 80%, the ADSCs were subcultured.

The isolated ADSCs were characterized by flow cytometry. The cells were incubated with human mesenchymal stem cell marker antibody panel for 30 min at room temperature, followed by centrifugation at 300 × *g* for 5 min, and the sample washed twice in 2 mL of PBS with 3% BSA. Then, the cells were incubated with secondary antibody for 30 min in the dark and were washed and resuspended in flow cytometry staining buffer for flow cytometric analysis using BD FACSDiva (v9.0).

### Exosome isolation and characterization

At the 3^rd^− 8^th^ passage, ADSCs were cultured in DMEM with 5% exosome-depleted FBS, and the spent media was collected after 48 h and centrifuged at 2000 × *g* for 10 min at 4 °C to remove cell debris. The supernatant was then ultrafiltered with a 100 kDa filter (Amicon 15), and the resulting solution was ultracentrifuged at 120,000 × *g* for 90 min at 4 °C to obtain exosome pellets. The exosome was resuspended in cold PBS and stored at −80 °C and used within one month.

To characterize the isolated exosome, nanoparticle tracking analysis (NTA) NTA (NanoSight NS300, Malvern Panalytical) was used to measure the concentration and size of exosome. TEM images were obtained by Scanning Transmission Electron Microscope (S)TEM (Thermo Fisher FEI, Tecnai G2 F20 S-TWIN). Samples were negatively stained with uranyl acetate (1 w/w%).

### Preparation of ONB and EBO

ONB was prepared through an ultrasonication method. Briefly, 40 mg of BSA and 80 mg of dextran sulfate were dissolved in 10 mL sterile 1 × PBS buffer (pH = 7.4), and then fully mixed using a magnetic stirrer overnight. Further, ultrasonication was conducted in ice bath with an ultrasonic cell disruptor (SFX250, Branson, USA) with 3 s on and 3 s off cycle, at 50% of amplitude for 7 min while oxygen was continuously introduced in this system. The resulting solution was filtered by a 0.22 μm cellulose acetate filter. Finally, ONB was collected through ultrafiltration with Amicon 15 filter (100 kDa).

EBO was generated by utilizing ultrasonication to coat the exosome membrane onto the ONB. Typically, exosomes and ONBs were thoroughly mixed at the ratio of 1:2, followed by ultrasonication. The ultrasonication process was carried out in an ice bath, with a cycle of 10 s on and 10 s off, at an amplitude of 50%, for a duration of 5 min. This cycle was repeated three times.

### Characterization of ONB and EBO

The hydrodynamic size distribution and zeta potential of ONB and EBO was obtained by the Litesizer™ 500 (Anton Paar). (S)TEM was employed to acquire TEM images, with the samples being negatively stained with 1 w/w% uranyl acetate. The NanoDrop™ One Microvolume UV-Vis Spectrophotometer (Catalog number: ND-ONE-W, ThermoFisher Scientific) was utilized to measure the UV-vis absorbance. The infrared spectra of BSA and ONB were obtained by a FT-IR spectrometer (Thermo-Nicolet Is-50 FTIR). Oxygen release property was monitored by Orion™ Versa Star Pro™ Dissolved Oxygen Electrochemistry Benchtop Meter (ThermoFisher Scientific) every 0.5 h in the duration of 10 h.

### EBO-Gel synthesis and characterization

PVA and gelatin were completely dissolved in distilled water, resulting in the final concentrations of 8 wt% and 2 wt%, respectively. Subsequently, a 2 wt% solution of borax was added to the mixture, facilitating the formation of the blank PVA/GA gel. To prepare the Exo-Gel, ONB-Gel, and EBO-Gel formulations, exosomes, ONBs, and EBO were evenly distributed within the PVA/GA mixture (10 wt% PVA and 2.5 wt% GA) and were further crosslinked by an equivalent volume of a 2 wt% borax solution.

All rheological tests were done on an ARES-G2 rotational rheometer (TA Instruments, DE) using a 25-mm parallel-plate geometry at 1 mm gap with sandpapers attached to both plates to prevent slippage. The gels were heated while being stirred at $T={T}_{g,{{{{{\rm{GA}}}}}}} \sim 60^\circ {{{{{\rm{C}}}}}}$ for 10 min and the samples were allowed to rest for 5 min before any rheological tests.

Oscillatory strain-amplitude sweep experiment was used to identify the critical yield-strain amplitude, ${\gamma }_{y}$, that approximates the boundary between linear and nonlinear regimes of both blank and EMO-laden PVA/GA gels. These tests were performed at $\omega=1{{{{{\rm{rad}}}}}}/{{{{{\rm{s}}}}}}$, and swept up from ${\gamma }_{0}=0.1\%$ to $500\%$. Both blank and EMO-laden gels exhibit ${\gamma }_{y} \sim 100\%$, see Supplementary Fig. 11. To assess the injectability, the complex viscosity, $|{\eta }^{*}|\equiv {\omega }^{-1}\sqrt{{{G}^{{\prime} }}^{2}+{{G}{{{{\hbox{'}}}{{\hbox{'}}}}}}^{2}}$, is next extracted from this strain-amplitude sweep experiment, and plotted against applied stress amplitude, ${\sigma }_{0},$ for both blank and EBO-hydrogels, Supplementary Fig. 12. Both hydrogels exhibit yield stress, ${\sigma }_{y},$ of about 2 kPa. Dramatic shear thinning occurs when the applied stress exceeds ${\sigma }_{y}$. To further assess the injectability, the stained hydrogel was loaded into a syringe with an inner diameter of 4.7 mm and subsequently extruded to conform to the shape of “UIUC”, see Fig. 3b. This subfigure shows the visual confirmation of shear thinning where the hydrogels were extrudable yet be able to retain their shape for at least 60 min on the substrate. A quantitative estimate of force required to extrude comes from a balance of applied force generating a pressure that must exceed the yield stress at the walls in circular pipe flow, $F=2{\sigma }_{y}\frac{L}{R}A=0.8{{{{{\rm{N}}}}}}$. Inverting this equation, we can estimate the applied wall shear stress during flow in the syringe, e.g. for a force on the order of a few Newtons, with L/R ~ 10, the wall shear stress is ~100,000 Pa. Our hydrogels exhibit good shape retention and shear thinning and thus suitable for in vivo application.

Linear viscoelasticity (LVE) was assessed using oscillatory shear frequency sweep experiments at small-strain amplitude (${\gamma }_{0}=5\%$). The LVE results are reported in Fig. 3h, and relaxation time is extracted from the crossover frequency, ${\omega }_{c}$: $\lambda=1/{\omega }_{c}\approx 1.5{{{{{\rm{s}}}}}}$. This signifies the solid-like behavior at a timescale shorter than $\lambda$, and the gels can flow at $\left|{\eta }^{*}\right|\approx {{{{\mathrm{2,000}}}}}{{{{{\rm{Pa}}}}}}\cdot {{{{{\rm{s}}}}}}$ from network rearranging at a longer timescale. Visual evidence confirms this behavior. The tilted vial in Fig. 3a, was used to make an estimate of the flow viscosity. Using gravitational flow down an incline, an upper bound viscosity is estimated to be $\eta=\rho g{h}^{2}\sin \theta /2{v}_{s}=1.9\times {10}^{4}{Pa}.s$ based on an assumed fluid thickness $h=10{{{{{\rm{mm}}}}}}$, tilt angle$\,\theta=40^\circ$, and maximum velocity based on a displacement of 5 mm over the 5 min of observation^59^. This viscosity is observed at the characteristic driving stress from gravity of $\sigma=\rho {gh}=98{{{{{\rm{Pa}}}}}}$.

Two methods were used to demonstrate the self-healing properties. The first method was to fragmentize the PVA-GA samples and reprocess them. To reprocess the samples, they were cut up into small pieces (about 5 × 5 mm squares) before blobbing and sandwiching them between two glass plates at 1.2 mm gap. For each reprocessing, about 10 wt% of fresh sample was added to compensate for small mass lost from the fragmentation. Linear viscoelasticity (vide supra) of the gels were measured as a probing surrogate metric for both virgin (a newly crosslinked gel) and reprocessed samples. Results reveal no significant difference up to the third reprocessing, with only a slight increase in LVE attributable to evaporation. The second method was to probe the rheological recovery from large deformation. For this experiment, a small-amplitude oscillatory shear (SAOS) at the strain amplitude of 5% was first applied for 60 s, and then large-amplitude oscillatory shear (LAOS) at 200% for 60 s, and lastly back to SAOS at 5% to probe the recovery of linear viscoelasticity. The recovery was monitored for 30 min until the PVA-GA gels reach a final logarithmic aging regime.

Collagen precursor solutions are made and kept on ice until the test time. Approximately 55 μL of the collagen precursor solutions was deposited on the bottom plate and the upper plate was then lowered into position. A thin layer of heavy mineral oil (FCC/USP, Fisher Chemical) was applied to the sample free surface to prevent evaporation. The temperature was then raised to 37 °C to initiate gelation.

ESEM (FEI Quanta FEG 450 ESEM) was employed to visualize microstructures of hydrogels. Macroscopic self-healing property was evaluated as follows: Two EBO-Gel specimens, each with a diameter of 2.2 cm, were fabricated and subsequently subjected to staining using crystal violet and red dyes, respectively. The hydrogels were then halved, and one half from each type of the hydrogel was juxtaposed. After 1 min, the reassembled gel was subjected to stretching to assess its healing capacity. The diffusion of dye was monitored for 100 min. The assessment of shape adaptability involved placing the hydrogel within various silicone molds to evaluate its ability to conform to different shapes. To demonstrate the injectability of the hydrogel, the stained hydrogel was loaded into a syringe with an inner diameter of 4.7 mm and subsequently extruded to form the shape of “UIUC”. Oxygen release within 48 h was monitored by Orion™ Versa Star Pro™ Dissolved Oxygen Electrochemistry Benchtop Meter (ThermoFisher Scientific) using a previously developed methodology^60^.

### In vitro degradation

For in vitro degradation analysis, EBO-Gels were submerged in a 5 mL PBS solution (pH = 7.4) with lysozyme (1000 U/mL). The incubation was in a constant-temperature incubator set at 37 °C. The hydrogels were taken out for lyophilization and weighed at intervals of 0, 1, 2, and 3 days. To maintain enzyme functionality, the lysozyme solution was refreshed daily. The in vitro degradation rate was determined using the formula:

$${{{{{\rm{Degradation\; rate}}}}}}=\left[\left({W}_{0}-{W}_{t}\right)/{W}_{0}\right]\times 100\%$$

where ${W}_{0}$ and ${W}_{t}$ represented the weights of the original and remaining hydrogels, respectively.

### Hemolysis assay

Fresh rat blood was obtained via cardiac puncture following euthanasia and anticoagulated using Heparin. Subsequently, erythrocytes were isolated through centrifugation at 10,000 × *g* for 5 min and washed using D-PBS. Next, 0.8 mL of diluted erythrocytes were exposed to 200 μL of Triton X-100, PBS, and different hydrogels. Following a 4-h incubation at room temperature, the mixtures were centrifuged at 10,016 × *g* for 5 min, and the absorbance of the supernatant was measured at 540 nm using a microplate reader. The hemolytic ratio was calculated using the following formula: Hemolysis ratio =$\,\left[\left({{{{{\rm{ODt}}}}}}-{{{{{\rm{ODn}}}}}}\right)/({{{{{\rm{ODp}}}}}}-{{{{{\rm{ODn}}}}}})\right]\times 100\%$. ${{{{{\rm{ODt}}}}}}$: OD value of tested samples, ${{{{{\rm{ODn}}}}}}$: OD value of negative control, ${{{{{\rm{ODp}}}}}}$: OD value of positive control.

### Cell viability

5 × 10^3^ HDF-a cells were pre-seeded into 96-well plate. After 24 h of culture, the medium was refreshed, and the HDF-a cells were co-incubated with hydrogels. MTT assay was performed to evaluate cell viability. In brief, 20 μL of the prepared 5 mg/mL MTT solution was added to each well of the plate and incubated at 37 °C for 4 h. Following the addition of 100 μL of DMSO, the plate was incubated for an additional 4 h at 37 °C in the dark. Ultimately, the absorbance at 490 nm was measured using a microplate reader (Synergy H1, BioTek Instruments).

### Cell proliferation

2 × 10^3^ HDF-a cells were pre-seeded into 96-well plate. After 24 h of normoxia incubation, the medium was refreshed, and the hydrogels were added. Then, the well plates were kept in hypoxia chamber with a mixture of 3% O_2_, 5% CO_2_, 92% N_2_ at 37 °C for 0 h, 24 h and 48 h, respectively. At different time point, WST-1 reagent was added and the optical density at 440 nm was then measured after 4 h of incubation according to manufacturer description.

### Cell migration

For scratching assay, HDF-a cells were pre-seeded in a 24-well plate. After the confluency reached to 80–90%, scratching wounds were created by 200 µL sterile pipette tips, and the culture medium was replaced with DMEM containing 1% FBS. Then, hydrogels were treated in the upper chamber of cell culture inserts (Millicell®, Sigma-Aldrich), and the plates were kept in hypoxia chamber with the hypoxic mixture as described. Cell migration was observed by microscope (ZEISS) at different timepoints and was quantified by ImageJ software (v1.53t).

For transwell migration assay, HDF-a cells were seeded in the upper chamber of cell culture inserts with a density of 1 × 10^4^ cells/mL, while the hydrogels were treated into the lower chamber with culture medium. After 24 h, the cells on the upper side of film were gently removed by a cotton swab, and the cells that migrated to the lower side were stained with 0.5% crystal violet for 1 h. The number of migrated cells were quantified by ImageJ software (v1.53t).

### Exosome uptake

To prepare Dio-labeled exosome, EBO was incubated with Dio (10 μM) for 20 min, followed by ultrafiltration using Amicon 15 at 1000 × *g* for 10 min. HDF-a cells were pre-seeded in 35 mm coverslip dishes and cultured with exosome-depleted medium. After attachment, Dio-labeled EBO was added and incubated for 6 h. The cells were fixed by 4% PFA after co-incubation, and the nucleus was stained with 4′,6-diamidino-2-phenylindole. Intracellular uptake was observed by Z-stack 3D confocal imaging with orthogonal views at the step size of 0.3 µm (Leica SP8).

### Immunofluorescence analysis

HDF-a cells were pre-seeded in 35 mm coverslip dishes. After attachment, hydrogels were added, and the imaging dishes were transferred to hypoxia chamber under the same hypoxic conditions as described. Following the completion of hypoxia incubation, the cells were washed with PBS and subsequently fixed with 4% paraformaldehyde. This was followed by permeabilization using 0.3% Triton X-100 for an additional 10 min. Then, the cells were incubated with a solution of 3% BSA in PBS for 1 h at 37 °C to prevent nonspecific binding. Next, various primary antibodies (anti-BrdU, anti-Lamp2) were applied to the cells and allowed to incubate overnight at 4 °C, and the cells were then incubated with secondary antibody for 1 h at 37 °C. For BrdU staining, the medium was replaced with 10 μM BrdU in culture medium before hydrogels were added. After fixation, the cells were incubated with 2 M HCl for 0.5 h for acid hydrolysis. Cytoskeleton was stained by Alexa Fluor™ Plus 555 Phalloidin for 20 min. Images were captured by a confocal laser scanning microscope (Leica SP8) and analyzed by ImageJ software (v1.53t).

### Tube formation assay

HUVECs for tube formation assay were used before passage 6. Typically, 100 μL of Matrigel (Corning) was added in a 96-well plate and incubated to gel at 37 °C for 1 h. 10,000 HUVECs were seeded into each well and hydrogels were then added. Plates were further transferred into the hypoxia chamber, and supplied with a mixture of 3% O_2_, 5% CO_2_, 92% N_2_ at 37 °C. After 6 h, brightfield images of tube formation were captured by Leica microscope. The number of branches and total length of branches was quantified by ImageJ software (v1.53t).

### In vitro hemostasis assay

Heparinized rat blood and hemocytes were mixed with Control, Carbopol gel (1 wt%), and EBO-Gel, respectively, at the volume ratio of 1:2. After 1 min, the vial was inversed, and the hemostasis property was observed.

### In vivo wound healing study

Male Sprague-Dawley rats of 250–300 g weight (8 weeks old) were obtained from Envigo Laboratory (Indianapolis, IN). All animal studies were performed in accordance with the guidelines of the Institutional Animal Care and Use Committee and the Division of Animal Resources at the University of Illinois (IACUC Protocol#: 23012). The rats were randomly sorted into five groups: NT, Blank-Gel, Exo-Gel, ONB-Gel, and EBO-Gel. The precursor solutions were prepared by thoroughly mixing 0.4 mL of PVA/GA (10 wt% PVA and 2.5 wt% GA) and 0.1 mL of nanoparticle solutions (Exo, ONB, and EBO) to achieve final concentrations of 8 wt% PVA and 2 wt% GA respectively. Hydrogels were created by combining the precursor solution with an equal volume of 2 wt% borax solution before application. Full-thickness wounds were then made on the back using a sterile disposable 8 mm diameter dermal biopsy punch (MEDLINE) to a depth of 2 mm. The hydrogels were applied to the wounds after surgery and refreshed every two days. In the Tegaderm group, no hydrogels were applied. The healing process was monitored by a digital camera, and the wound area was quantified by ImageJ software.

On day 4 and day 14, animals were euthanized, and the wound tissues were collected. Then, the tissues were fixed in 10% formalin neutral buffered solution (Sigma-Aldrich) and further embedded in paraffin and sectioned for H&E/Masson Trichrome staining according to the manufacturer manual. The histology images were acquired by microscope. The Scar Index (mm), denoted as Scar Area (mm²) divided by the Average Dermal Thickness (mm). Dermal thickness, epidermal thickness and scar area were quantified by NDP View 2 software. The collagen volume fraction was quantified by ImageJ software (v1.53t).

### Statistics and reproducibility

Statistical analysis was performed using GraphPad Prism (v9.0). The differences between any two groups were analyzed using a two-tailed, unpaired Student’s $t$ test. For comparisons involving more than two groups, one-way ANOVA with Tukey’s multiple comparisons was employed. Grouped data were assessed using two-way ANOVA with Dunnett’s multiple comparisons. $P < 0.05$ was considered statistically significant. The presented data indicate the mean ± SD from at least two independent experiments.

### Reporting summary

Further information on research design is available in the Nature Portfolio Reporting Summary linked to this article.

### Supplementary information (Please note: The SI document still has the old title - Please modify the title in SI to be consistent with the updated title in the main manuscript)

Supplementary Information

Peer Review File

Description of Additional Supplementary Files

Supplementary Video 1

Supplementary Video 2

Reporting Summary

### Source data

Source data

## Data Availability

The authors declare that all the data supporting the findings of this study are available within the article and its supplementary information. Source data are provided as a Source Data file. Source data are provided with this paper.
