# Supplementary material for: Exosome-coated oxygen nanobubble-laden hydrogel augments intracellular delivery of exosomes for enhanced wound healing
Source: Nat Commun. 2024 Apr 23;15:3435. doi: 10.1038/s41467-024-47696-5 (PMC11039765; doi:10.1038/s41467-024-47696-5)
Supplement: Supplementary file 1 — Supplementary Information [file 41467_2024_47696_MOESM1_ESM.pdf]

Exosome-coated Oxygen Nanobubble-laden Hydrogel Augments Intracellular Delivery of Exosomes for Enhanced Wound Healing

Xiaoxue Han[1,2.3,4], Chaimongkol Saengow[3,5], Leah Ju[1,2], Wen Ren[1,2], Randy H. Ewoldt[3,5], Joseph Irudayaraj[1,2,3,4,6 *]

[1]Department of Bioengineering, 1102 Everitt Lab, 1406 W. Green St., University of Illinois at Urbana-Champaign, Urbana, IL, 61801, USA

[2]Biomedical Research Center, Mills Breast Cancer Institute, Carle Foundation Hospital, Urbana, IL, 61801, USA

[3]Cancer Center at Illinois, Beckman Institute, Urbana, IL, 61801, USA

[4]Holonyak Micro and Nanotechnology Laboratory, Carle R. Woese Institute for Genomic Biology, Urbana, IL, 61801, USA

[5]Department of Mechanical Science and Engineering, University of Illinois at Urbana-Champaign, Urbana, IL, 61801, USA

[6]Carle Illinois College of Medicine, University of Illinois at Urbana-Champaign, Urbana, IL, 61801, USA

Author for correspondence: Dr. Joseph Irudayaraj, (217) 333-1867, jirudaya@illinois.edu

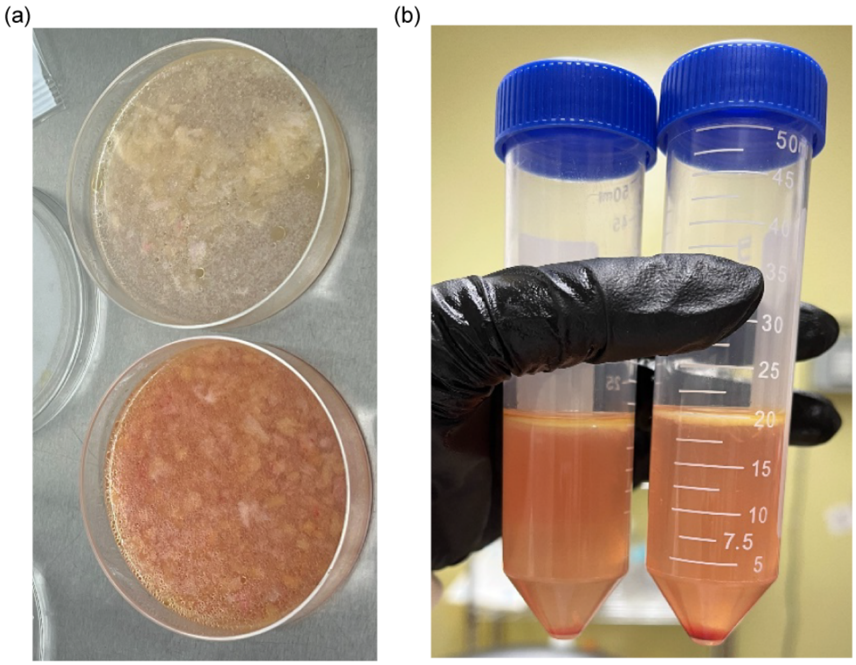

Supplementary Figure 1. The photos of (a) digested adipose tissue and (b) centrifuged cell pellet (mesenchymal stem cells were included).

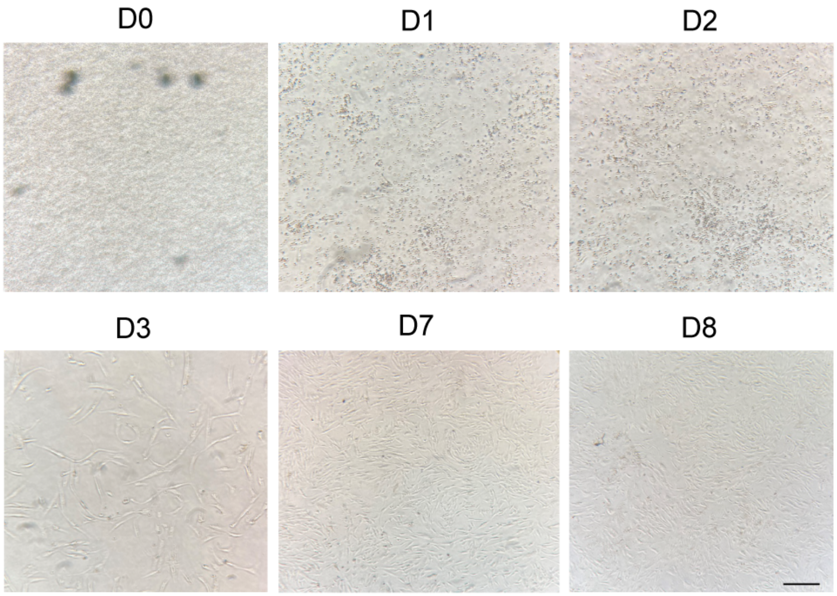

Supplementary Figure 2. The morphology of ADSCs from day 0 to day 8. Scale bar: 200 μm.

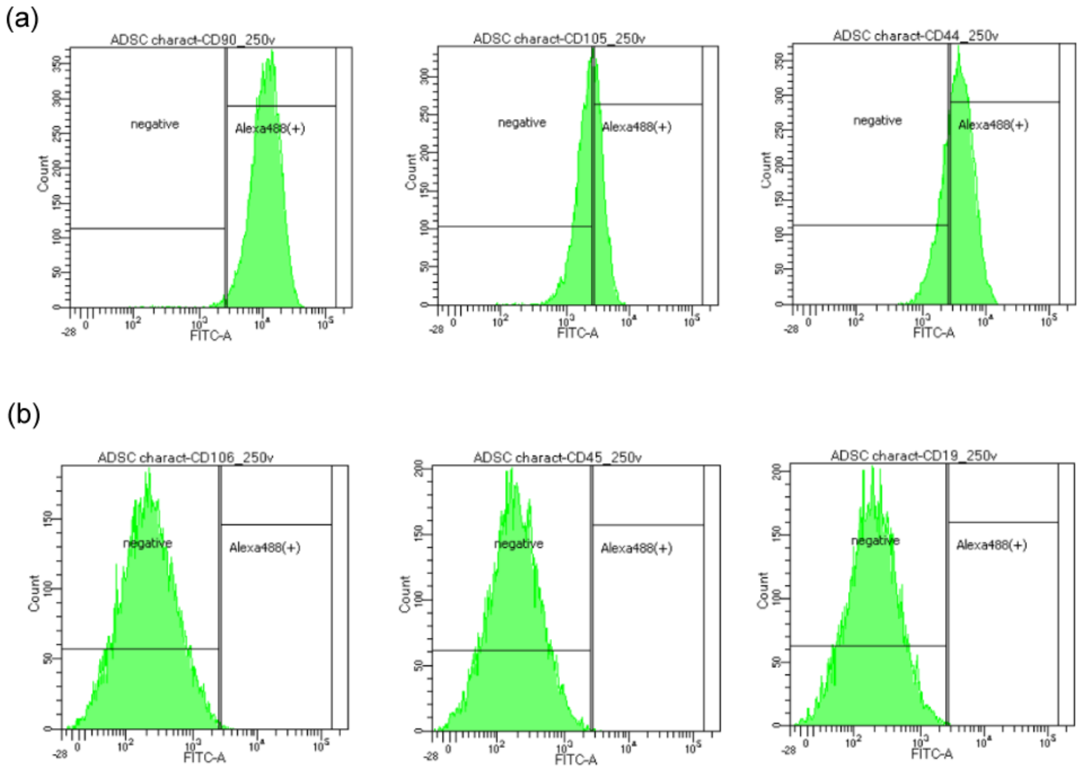

Supplementary Figure 3. Surface marker detection of isolated ADSCs, including a panel of (a) positive markers of CD90, CD105, and CD44, and (b) negative markers of CD 106, CD45, and CD19.

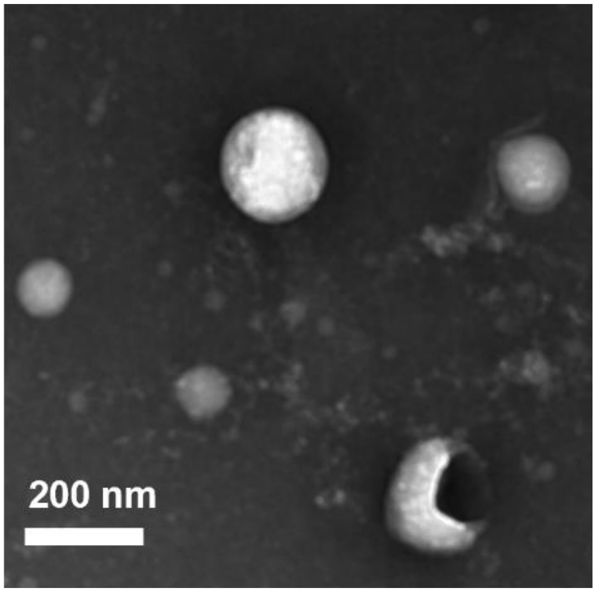

Supplementary Figure 4. Typical exosome structure observed under TEM. Scale bar: 200 nm. Representative images are shown from two independent experiments with similar results.

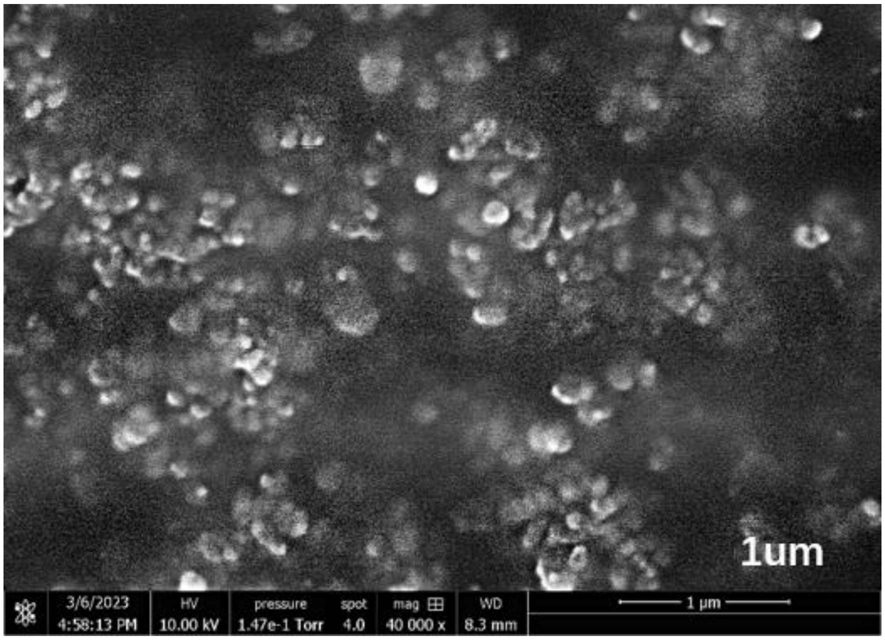

Supplementary Figure 5. SEM images of EBO. Scale bar = 1 μm. Representative images are shown from two independent experiments with similar results.

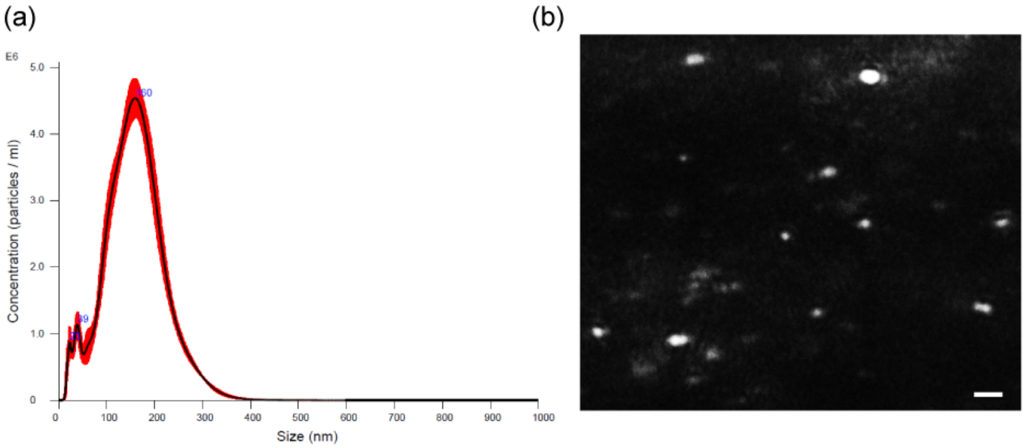

Supplementary Figure 6. (a) Particle concentrations of EBO (n = 5 independent experiments). (b) Representative images of EBO under NTA. Scale bar: 200 nm. Data are presented as mean ± SD (a). Representative images are shown from two independent experiments with similar results (b). Source data are provided as a Source Data file.

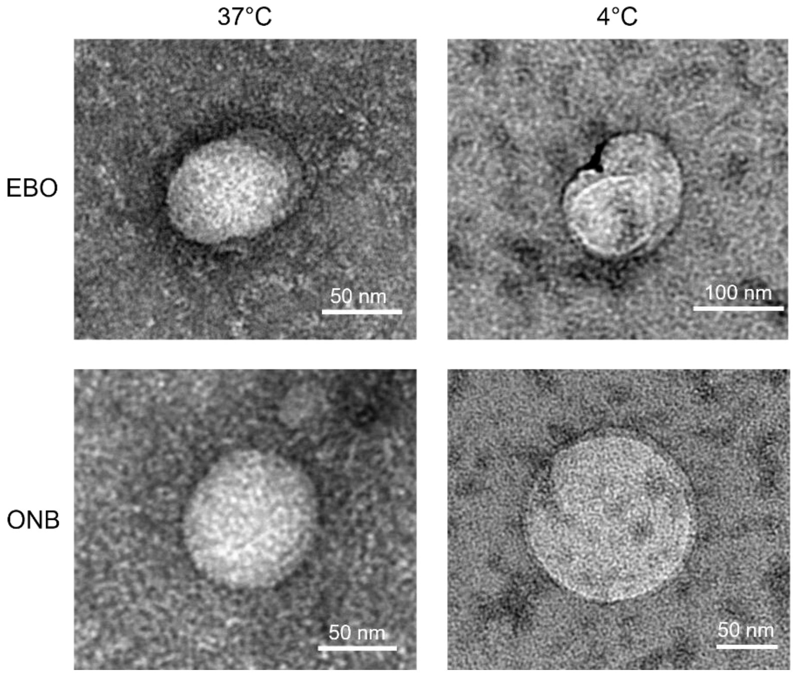

Supplementary Figure 7. TEM images of EBO and ONB following storage for 2 days at 37 °C or 1 month at 4 °C. Representative images are shown from two independent experiments with similar results.

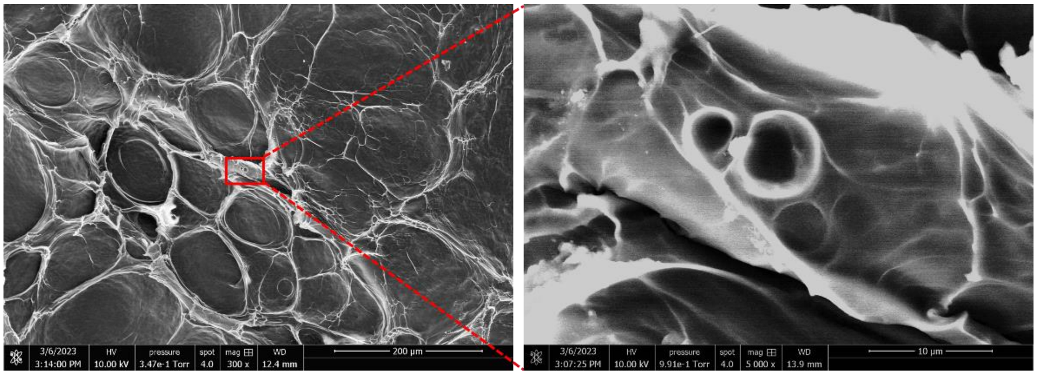

Supplementary Figure 8. SEM images of Blank-Gel (left) and the magnified image (right). Representative images are shown from two independent experiments with similar results.

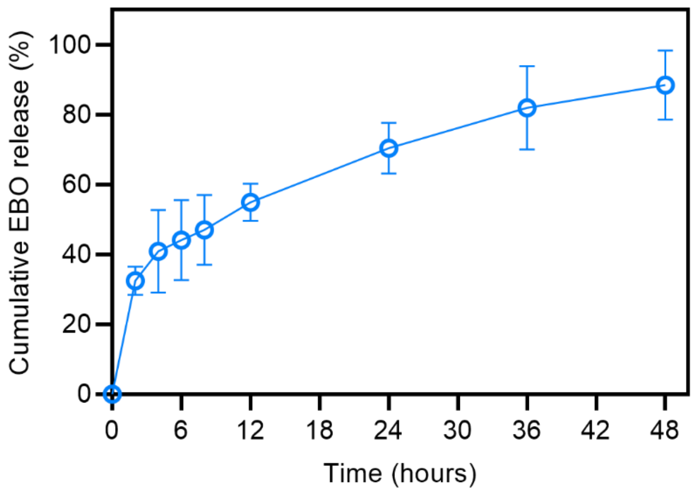

Supplementary Figure 9. EBO nanoparticle release profile from EBO-Gel within 48 hrs at a pH of 7.4, 37 °C (n = 3 independent samples). Data are presented as mean ± SD. Source data are provided as a Source Data file.

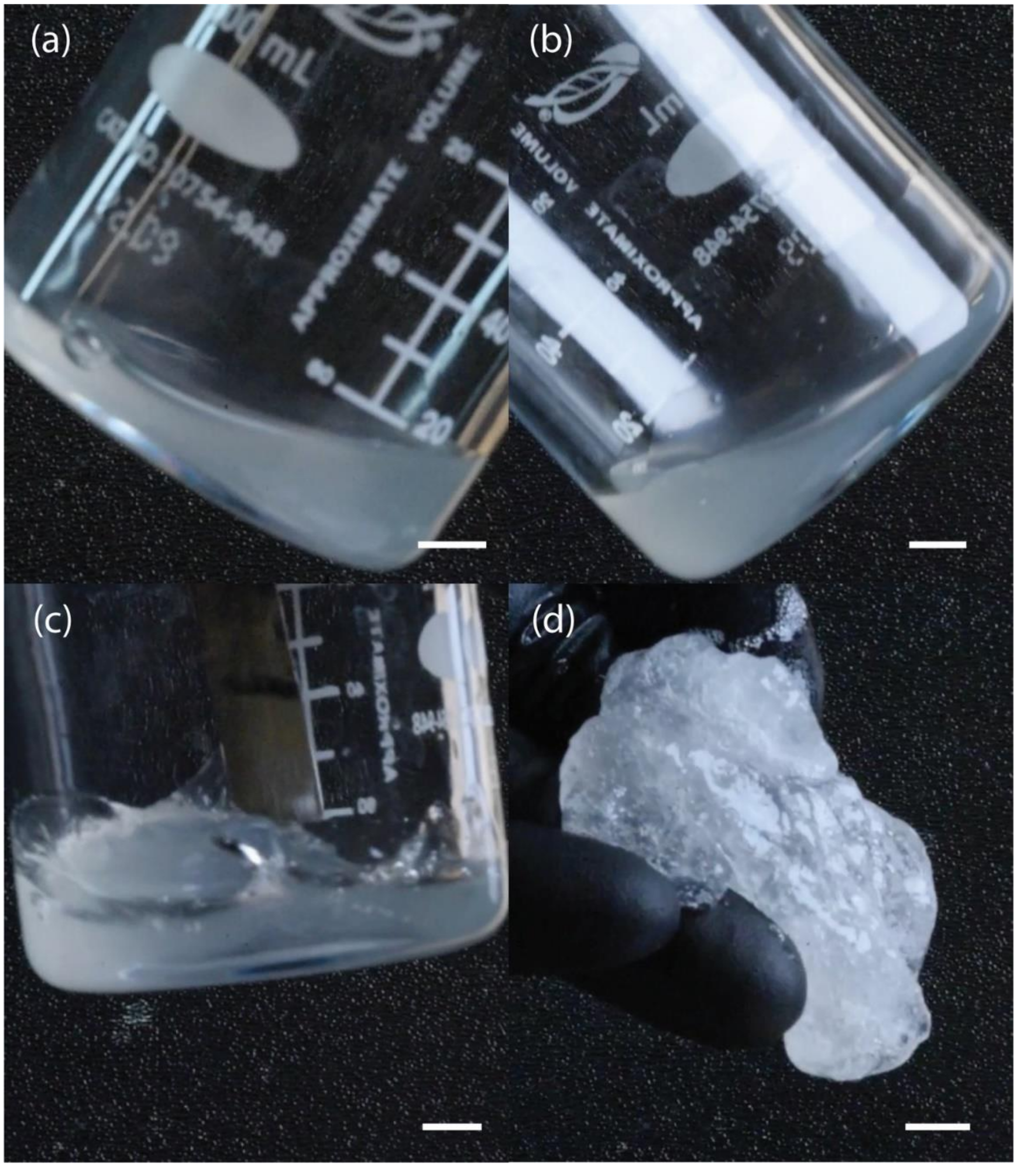

Supplementary Figure 10. Visual confirmation of crosslinks of hydrogels after mixing. (a) and (b) shows the flowable precursor mixture before sodium borate addition; (c) 4 seconds after sodium borate addition shows significant increases in viscosity, confirming crosslinking; (d) Well mixed final gel after letting the gel rest for 5 minutes. The crosslink reaction happened rapidly after sodium borate addition. All scalebars are 10 mm.

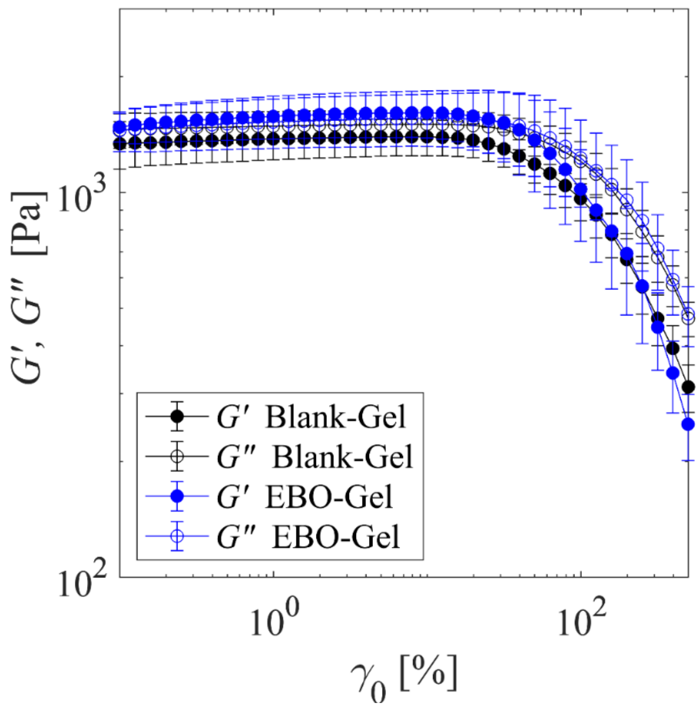

Supplementary Figure 11: Strain amplitude sweep of the PVA-GA-Borax hydrogels at 1 rad/s (n = 3 independent experiments). Critical yielding strain amplitude, γ_y, is estimated to be about 100%, marking the boundary of linear and nonlinear regimes. Subtle increases in both moduli are attributable to physical hydrogen bonds. Data are presented as mean ± SD. Source data are provided as a Source Data file.

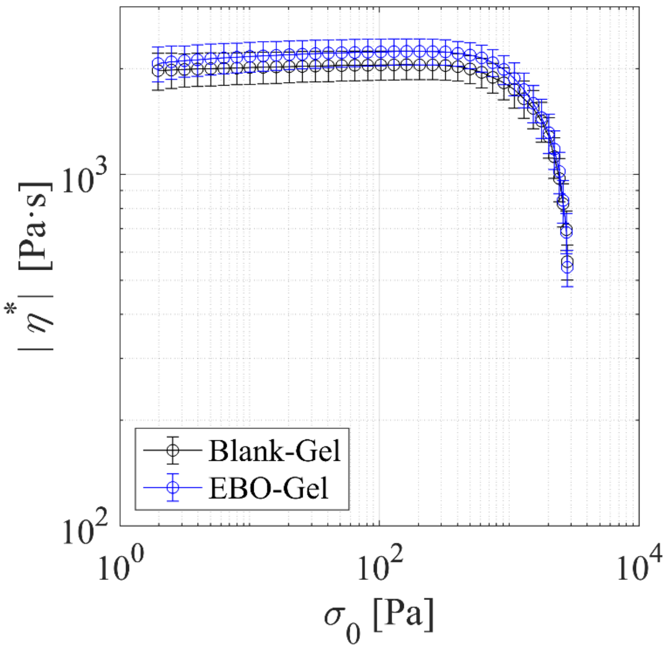

Supplementary Figure 12. Magnitude of complex viscosity (n = 3 independent experiments), $|\eta^*|$, as a function of applied stress amplitude, σ_0, showing high shear thinning behavior when σ_0 surpasses the thresholding yield stress, $\sigma_y \approx 2\ \mathrm{kPa}$, of both Blank and EBO hydrogels. The ordinate data is replotted from data in Figure S11, where $|\eta^*| \equiv \omega^{-1}\sqrt{G'^2 + G''^2}$. Data are presented as mean ± SD. Source data are provided as a Source Data file.

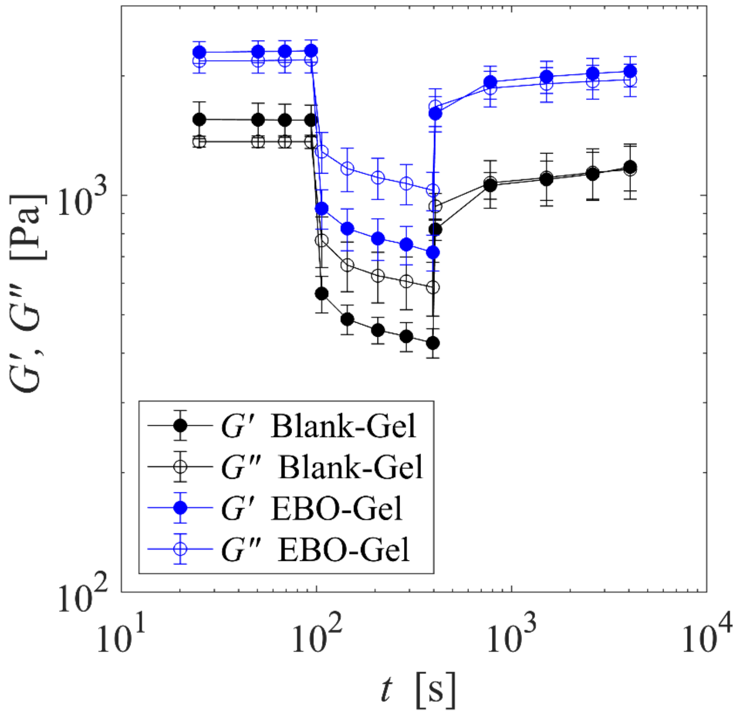

Supplementary Figure 13: Nonlinear rheology recovery signifying self-healing from SAOS-LAOS-SAOS experiment at 1 rad/s (n = 3 independent experiments). The gel shows instant recovery at the outset of second SAOS and continues aging from hydrogen bonding. Data are presented as mean ± SD. Source data are provided as a Source Data file.

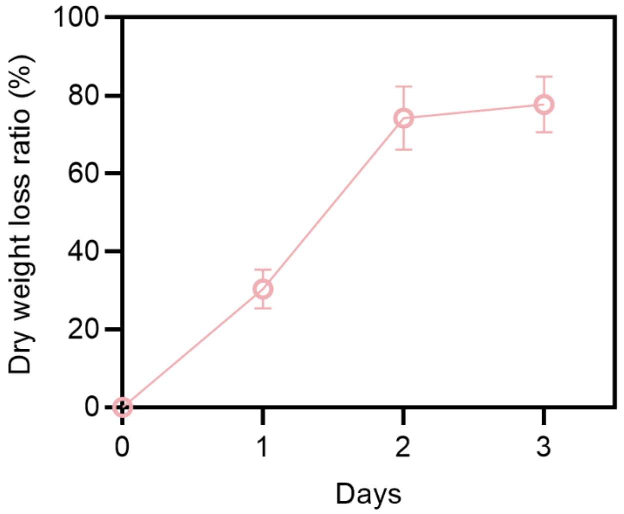

Supplementary Figure 14. In vitro degradation rate of EBO-Gel within 3 days in PBS at 37 °C (n = 3 independent samples). Data are presented as mean ± SD. Source data are provided as a Source Data file.

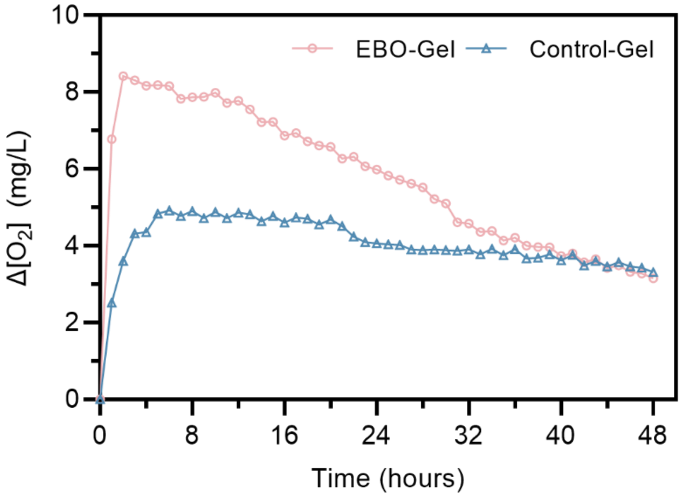

Supplementary Figure 15. Oxygen concentration profile of EBO-Gel and Control-Gel (Blank-Gel with oxygenated exo/BSA/Dex solution) in 48 hrs. Source data are provided as a Source Data file.

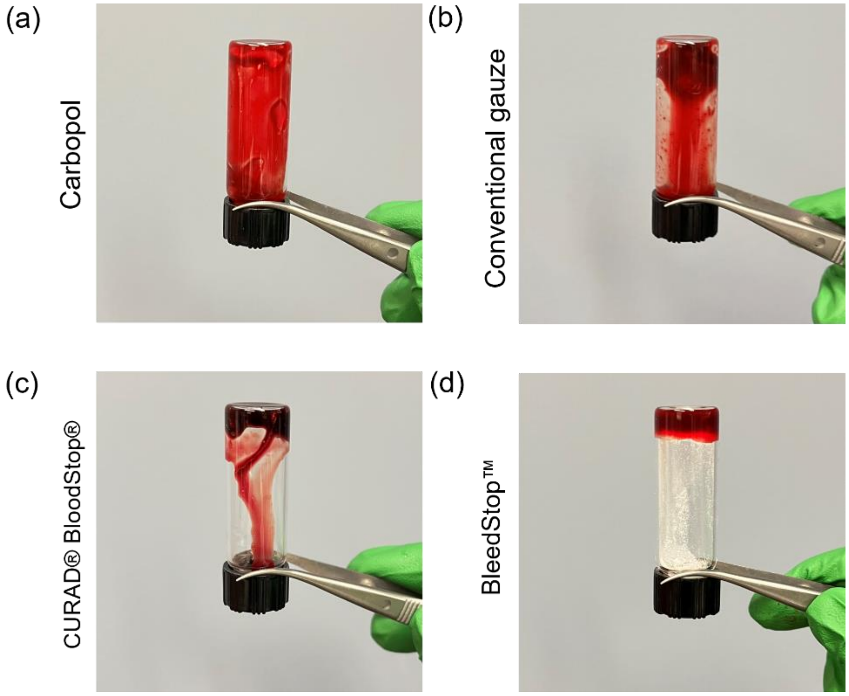

Supplementary Figure 16. In vitro procoagulant effects of different treatments including (a) A non-hemostatic hydrogel, Carbopol; (b) Conventional gauze; (c) CURAD® BloodStop® Hemostatic Gauze; and (d) BleedStop™. Representative images are shown from three independent experiments with similar results.

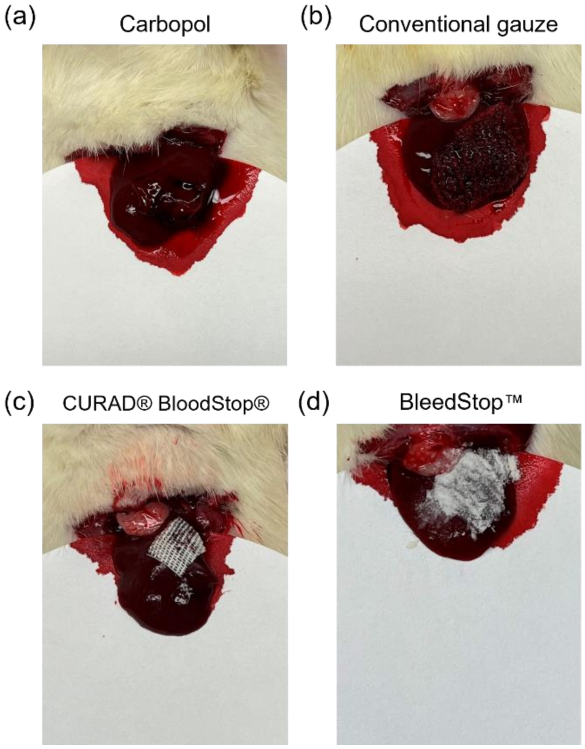

Supplementary Figure 17. In vivo hemostasis evaluation of different treatments including (a) A non-hemostatic hydrogel, Carbopol; (b) Conventional gauze; (c) CURAD® BloodStop® Hemostatic Gauze; and (d) BleedStop™. Representative images are shown from three independent experiments with similar results.

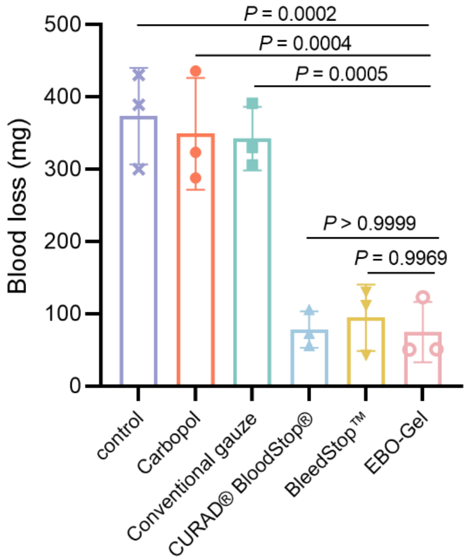

Supplementary Figure 18. Quantitation of blood loss of various treatments (n = 3 biologically independent samples). Data are presented as mean ± SD. Statistical analysis was performed by one-way ANOVA with Tukey's multiple comparisons. Source data are provided as a Source Data file.

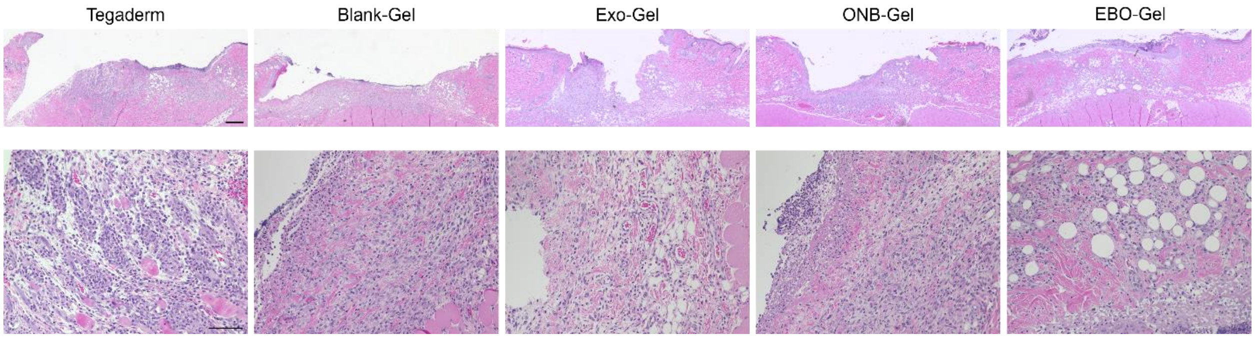

Supplementary Figure 19. Representative (Row 1) and the corresponding magnified images (Row 2) of H&E staining of the wound tissues on Day 4 post-treatment. Scale bar: 500 μm for Row 1 and 100 μm for Row 2. Representative images are shown from three independent experiments with similar results.

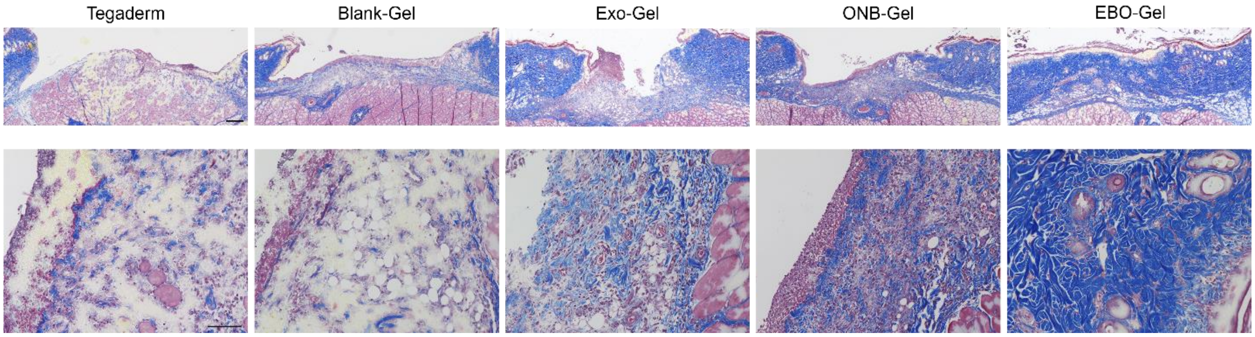

Supplementary Figure 20. Representative (Row 1) and the corresponding magnified images (Row 2) of Masson's trichrome staining of the wound tissues on Day 4 post-treatment. Scale bar: 500 μm for Row 1 and 100 μm for Row 2. Representative images are shown from three independent experiments with similar results.

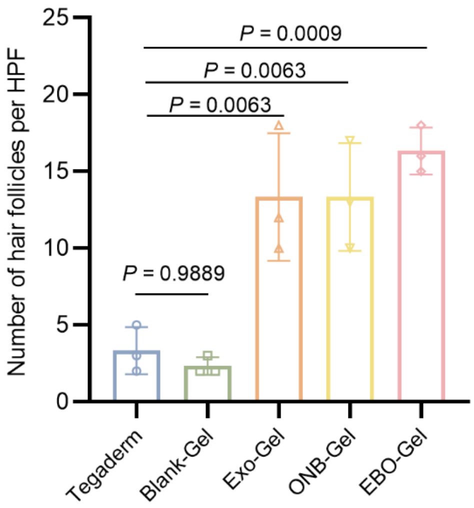

Supplementary Figure 21. Quantitative analysis of hair follicle counts per high-power field (HPF) (n = 3 biologically independent samples). Data are presented as mean ± SD. Statistical analysis was performed by one-way ANOVA with Tukey's multiple comparisons. Source data are provided as a Source Data file.

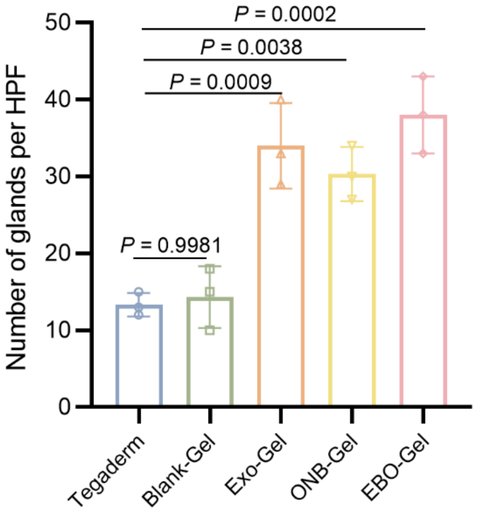

Supplementary Figure 22. Quantitative analysis of gland count (sebaceous glands and sweat glands) per high-power field (HPF) (n = 3 biologically independent samples). Data are presented as mean ± SD. Statistical analysis was performed by one-way ANOVA with Tukey's multiple comparisons. Source data are provided as a Source Data file.

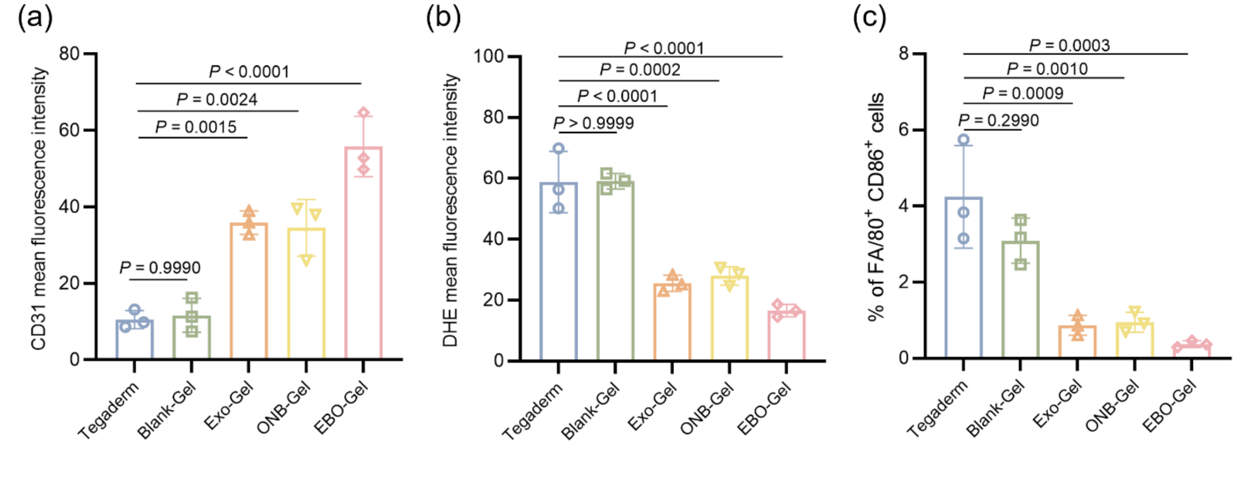

Supplementary Figure 23. Quantification of fluorescent intensity of (a) CD31 and (b) DHE. (c) Quantification of the percentage of M1 macrophages (n = 3 biologically independent samples). Data are presented as mean ± SD. Statistical analysis was performed by one-way ANOVA with Tukey's multiple comparisons. Source data are provided as a Source Data file.

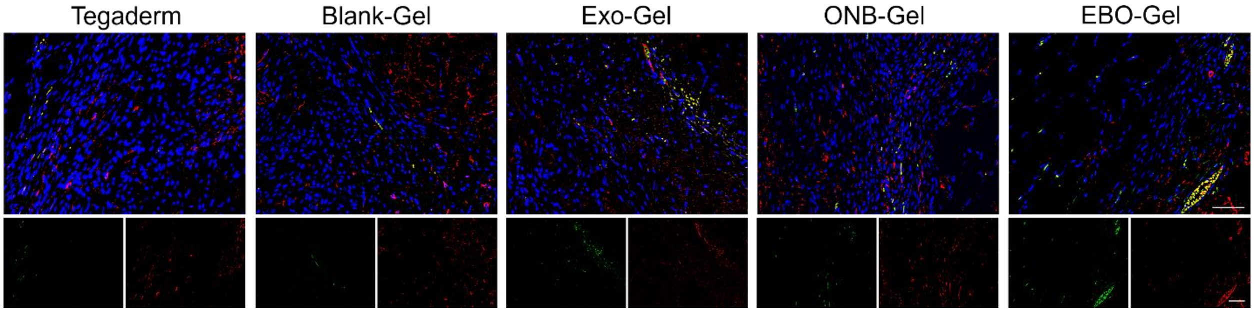

Supplementary Figure 24. Wound tissue immunofluorescent staining of CD206 (green) and F4/80 (red). Cell nuclei were stained with DAPI (blue). Scale bar: 50 μm. Representative images are shown from three independent experiments with similar results.

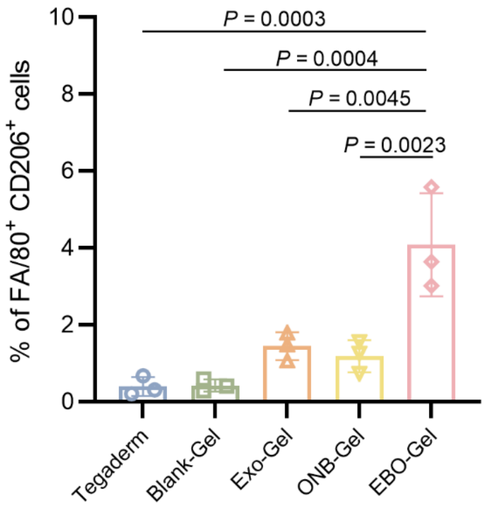

Supplementary Figure 25. Quantification of the percentage of M2 macrophages (n = 3 biologically independent samples). Data are presented as mean ± SD. Statistical analysis was performed by one-way ANOVA with Tukey's multiple comparisons. Source data are provided as a Source Data file.

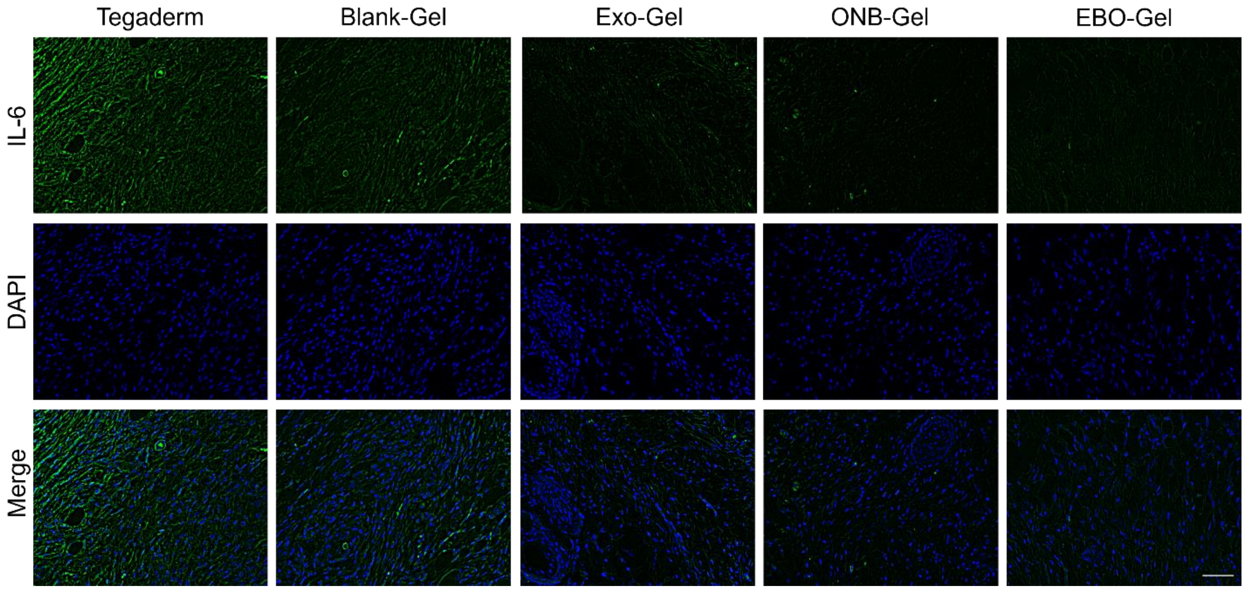

Supplementary Figure 26. Wound tissue immunofluorescent staining of IL-6 (green). Cell nuclei were stained with DAPI (blue). Scale bar: 50 µm. Representative images are shown from three independent experiments with similar results.

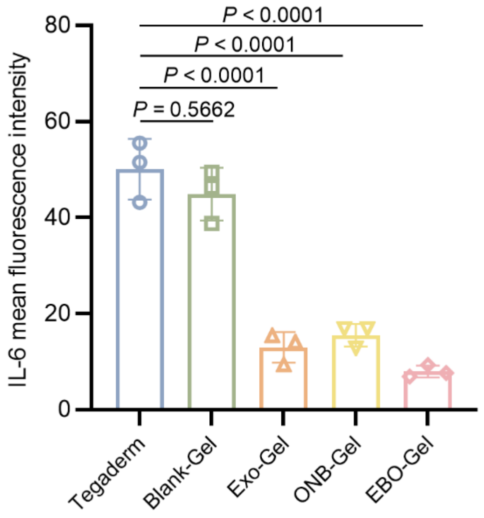

Supplementary Figure 27. Quantification of fluorescent intensity of IL-6 staining (n = 3 biologically independent samples). Data are presented as mean ± SD. Statistical analysis was performed by one-way ANOVA with Tukey's multiple comparisons. Source data are provided as a Source Data file.

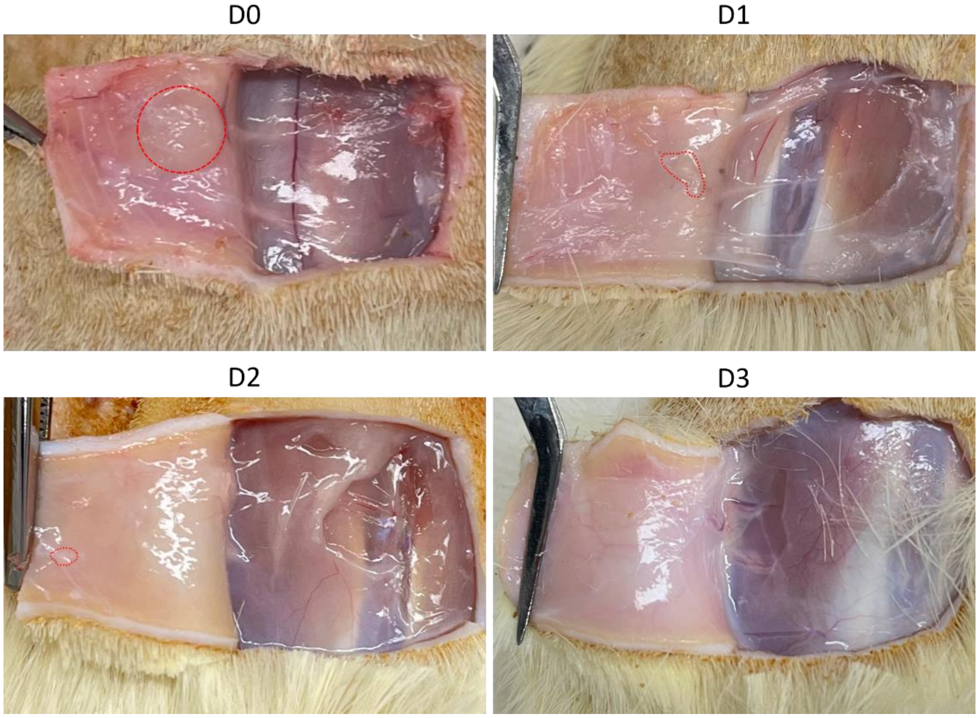

Supplementary Figure 28. Overall observation of in vivo degradation of EBO-Gel within 3 days (Residual gel is indicated by the red area). Representative images are shown from three independent experiments with similar results.

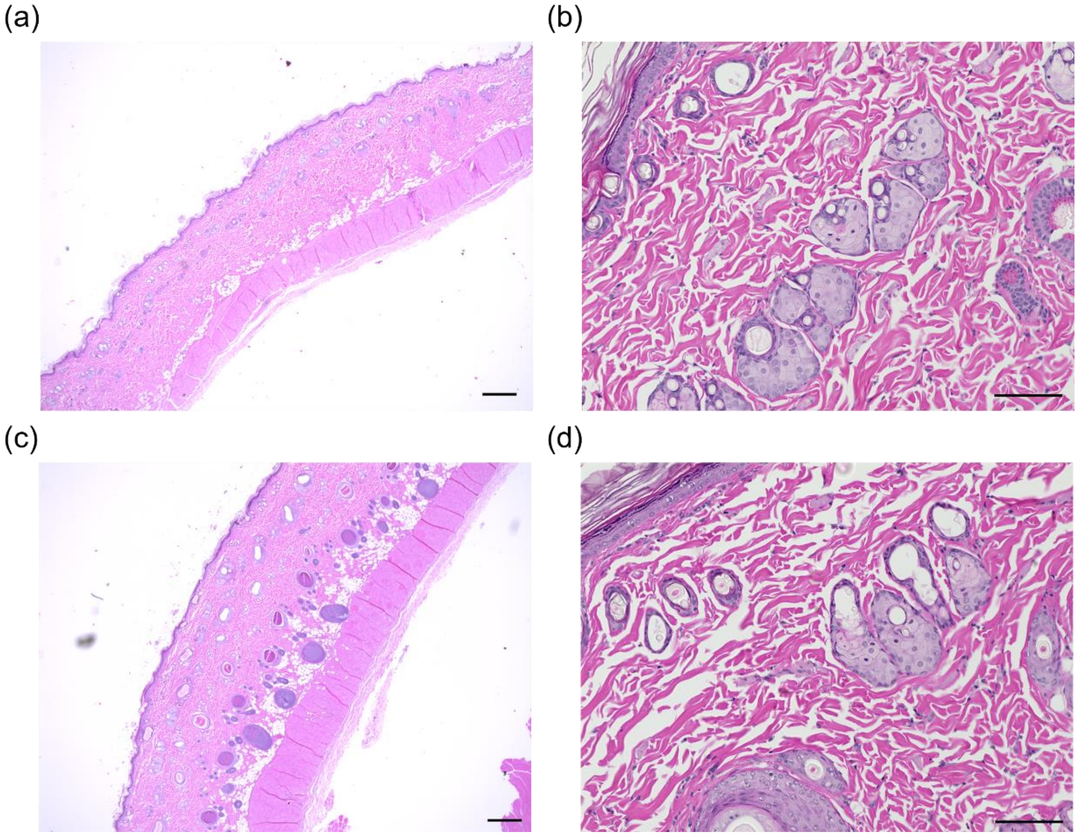

Supplementary Figure 29. H&E staining of the skin and subcutis tissues of (a) and (b) normal group, (c) and (d) EBO-Gel injected group at Day 3 post-injection. Scale bar: 500 µm (a and c), 100 µm (b and d). Representative images are shown from three independent experiments with similar results.

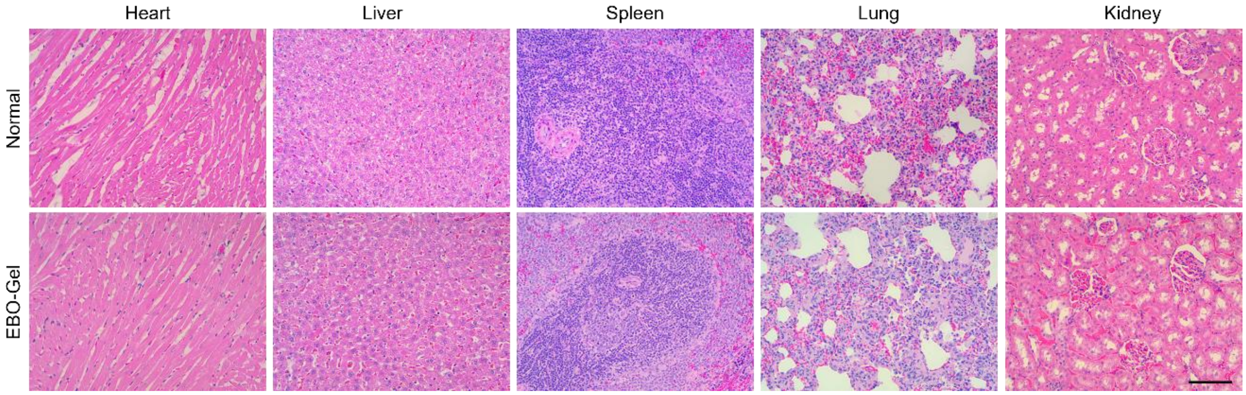

Supplementary Figure 30. Images of H&E-stained major organs collected from unwounded healthy rat and EBO-Gel treated rat at day 14 post-surgery. Scale bar: 100 µm. Representative images are shown from three independent experiments with similar results.
