# Supplementary material for: Exosome-coated oxygen nanobubble-laden hydrogel augments intracellular delivery of exosomes for enhanced wound healing
Source: Nat Commun. 2024 Apr 23;15:3435. doi: 10.1038/s41467-024-47696-5 (PMC11039765; doi:10.1038/s41467-024-47696-5)
Supplement: Supplementary file 3 — Description of Additional Supplementary Files [file 41467_2024_47696_MOESM3_ESM.docx]

**Description of Additional Supplementary Files**

**File Name: Supplementary Video 1
Description:** Z-stack uptake after 6h incubation.

**File Name: Supplementary Video 2
Description:** Gel formation.
