# Supplementary material for: Exosome-coated oxygen nanobubble-laden hydrogel augments intracellular delivery of exosomes for enhanced wound healing
Source: Nat Commun. 2024 Apr 23;15:3435. doi: 10.1038/s41467-024-47696-5 (PMC11039765; doi:10.1038/s41467-024-47696-5)
Supplement: Supplementary file 6 — Reporting Summary [file 41467_2024_47696_MOESM6_ESM.pdf]

Corresponding author(s): Joseph Irudayaraj

Last updated by author(s): Mar 21, 2024

Reporting Summary

Nature Portfolio wishes to improve the reproducibility of the work that we publish. This form provides structure for consistency and transparency in reporting. For further information on Nature Portfolio policies, see our Editorial Policies and the Editorial Policy Checklist.

Statistics

For all statistical analyses, confirm that the following items are present in the figure legend, table legend, main text, or Methods section.

n/a	Confirmed	
☐	☒	The exact sample size (*n*) for each experimental group/condition, given as a discrete number and unit of measurement
☐	☒	A statement on whether measurements were taken from distinct samples or whether the same sample was measured repeatedly
☐	☒	The statistical test(s) used AND whether they are one- or two-sided<br>*Only common tests should be described solely by name; describe more complex techniques in the Methods section.*
☒	☐	A description of all covariates tested
☐	☒	A description of any assumptions or corrections, such as tests of normality and adjustment for multiple comparisons
☐	☒	A full description of the statistical parameters including central tendency (e.g. means) or other basic estimates (e.g. regression coefficient) AND variation (e.g. standard deviation) or associated estimates of uncertainty (e.g. confidence intervals)
☐	☒	For null hypothesis testing, the test statistic (e.g. *F*, *t*, *r*) with confidence intervals, effect sizes, degrees of freedom and *P* value noted<br>*Give P values as exact values whenever suitable.*
☒	☐	For Bayesian analysis, information on the choice of priors and Markov chain Monte Carlo settings
☒	☐	For hierarchical and complex designs, identification of the appropriate level for tests and full reporting of outcomes
☒	☐	Estimates of effect sizes (e.g. Cohen's *d*, Pearson's *r*), indicating how they were calculated

Our web collection on statistics for biologists contains articles on many of the points above.

Software and code

Policy information about availability of computer code

Data collection	No computer code was used. Litesizer™ 500 (Anton Paar) for the hydrodynamic diameter and zeta potential; NanoSight NS300 (Malvern Panalytical) for the particle concentration; Thermo-Nicolet Is-50 FTIR for IR spectrum; Thermo Fisher FEI, Tecnai G2 F20 S-TWIN for TEM; FEI Quanta FEG 450 ESEM for SEM; ARES-G2 rotational rheometer (TA Instruments, DE) for rheology studies; Orion™ Versa Star Pro™ Dissolved Oxygen Electrochemistry Benchtop Meter (ThermoFisher Scientific) for oxygen measure; BD LSRFortessa for flow cytometry; Leica LAS X (v3.0) for collecting confocal images.
Data analysis	No computer code was used. Image J (v1.53t), Zeiss Zen (v2.0), and BZ-X800 Analyzer (v1.1.1.8), Graphpad Prism (v9.0), BD FACSDiva 9.0, Flowjo V10.8 and routine softwares are used and described in the manuscript and reported below.

For manuscripts utilizing custom algorithms or software that are central to the research but not yet described in published literature, software must be made available to editors and reviewers. We strongly encourage code deposition in a community repository (e.g. GitHub). See the Nature Portfolio guidelines for submitting code & software for further information.

Data

Policy information about availability of data

All manuscripts must include a data availability statement. This statement should provide the following information, where applicable:

- Accession codes, unique identifiers, or web links for publicly available datasets
- A description of any restrictions on data availability
- For clinical datasets or third party data, please ensure that the statement adheres to our policy

The authors declare that all the data supporting the findings of this study are available within the article and its supplementary information. Source data are provided as a Source Data file.

Research involving human participants, their data, or biological material

Policy information about studies with human participants or human data. See also policy information about sex, gender (identity/presentation), and sexual orientation and race, ethnicity and racism.

Reporting on sex and gender	N/A
Reporting on race, ethnicity, or other socially relevant groupings	N/A
Population characteristics	N/A
Recruitment	N/A
Ethics oversight	N/A

Note that full information on the approval of the study protocol must also be provided in the manuscript.

Field-specific reporting

Please select the one below that is the best fit for your research. If you are not sure, read the appropriate sections before making your selection.

☒ Life sciences ☐ Behavioural & social sciences ☐ Ecological, evolutionary & environmental sciences

For a reference copy of the document with all sections, see nature.com/documents/nr-reporting-summary-flat.pdf

Life sciences study design

All studies must disclose on these points even when the disclosure is negative.

Sample size	The sample size was established based on the pilot study and the previous experimental experiences (Nature Communications, 14(1), 7658.; Nat. Commun. 14 (2023) 3431; Bioactive Materials, 35, 67-81). A power analysis (using G*POWER 3.1 software) for a one-way ANOVA was also used to determine sample size. Generally 3-5 independent replicates for in vitro experiments, and 3-6 independent biological replicates for in vivo experiments, as described in the Method and figure captions.
Data exclusions	No data were excluded.
Replication	All of the studies are repeated at lease twice as indicated in the figure legends. All attempts at replication were successful.
Randomization	All samples/cells/animals were randomly allocated into different groups before treatment.
Blinding	The investigators are aware of the experimental groups because the experimental results are not affected by blinding. The experimental procedure and data analysis were conducted with a commitment to impartiality to the greatest extent possible.

Reporting for specific materials, systems and methods

We require information from authors about some types of materials, experimental systems and methods used in many studies. Here, indicate whether each material, system or method listed is relevant to your study. If you are not sure if a list item applies to your research, read the appropriate section before selecting a response.

Materials & experimental systems

n/a	Involved in the study
☐	☒ Antibodies
☐	☒ Eukaryotic cell lines
☒	☐ Palaeontology and archaeology
☐	☒ Animals and other organisms
☒	☐ Clinical data
☒	☐ Dual use research of concern
☒	☐ Plants

Methods

n/a	Involved in the study
☒	☐ ChIP-seq
☐	☒ Flow cytometry
☒	☐ MRI-based neuroimaging

Antibodies

Antibodies used

CD31 Monoclonal Antibody (WM59, Thermofisher, Catalog # MA1-26196, Lot: YG3995821), Dilution: 1:100
CD86 Polyclonal Antibody (Thermofisher, Catalog # PA5-114995, Lot: YK4124233A), Dilution: 1:100
IL-6 Monoclonal Antibody (11-D4, Thermofisher, Catalog # MA5-45069, Lot: YK4138019A), Dilution: 1:100
LAMP2 Monoclonal Antibody (H4B4, Thermofisher, Catalog # MA1-205, Lot: YC373894), Use a concentration of 2 μg/mL
CD206 (MMR) Recombinant Rabbit Monoclonal Antibody (BLR109H, Thermofisher, Catalog # MA5-44409, Lot: YK4124392), Dilution: 1:100
F4/80 Monoclonal Antibody (BM8), eBioscience™, Catalog # 14-4801-82, Lot: 2739530, Use a concentration of 10 μg/mL
Human Mesenchymal Stem Cell Marker Antibody Panel (R&D systems, Catalog #: SC017), Dilution: 1:100
BrdU Monoclonal Antibody (MoBU-1) (Thermofisher, Catalog # B35128), Dilution: 1:100
Goat anti-Mouse IgG (H+L) Cross-Adsorbed Secondary Antibody, FITC, Thermofisher, Catalog # F-2761, Use a concentration of 1 μg/mL
Goat anti-Rabbit IgG (H+L) Secondary Antibody, FITC, Thermofisher, Catalog # 65-6111, Dilution: 1:50
Goat anti-Rat IgG (H+L) Cross-Adsorbed Secondary Antibody, Alexa Fluor™ 555, Thermofisher, Catalog # A-21434, Use a concentration of 1 μg/mL

Validation

All antibodies are commercially available and validated by the respective manufacturers. The validation and quality control are available on the manufactures' website and datasheet. All antibodies were previously reported and routinely used for the application in this study.
CD31:
https://www.thermofisher.com/antibody/product/CD31-Antibody-clone-WM59-Monoclonal/MA1-26196
CD86:
https://www.thermofisher.com/antibody/product/CD86-Antibody-Polyclonal/PA5-114995
IL-6:
https://www.thermofisher.com/antibody/product/IL-6-Antibody-clone-11-D4-Monoclonal/MA5-45069
LAMP2:
https://www.thermofisher.com/antibody/product/LAMP2-Antibody-clone-H4B4-Monoclonal/MA1-205
CD206:
https://www.thermofisher.com/antibody/product/CD206-MMR-Antibody-clone-BLR109H-Recombinant-Monoclonal/MA5-44409
F4/80:
https://www.thermofisher.com/antibody/product/F4-80-Antibody-clone-BM8-Monoclonal/14-4801-82
Human Mesenchymal Stem Cell Marker Antibody Panel:
https://www.rndsystems.com/products/human-mesenchymal-stem-cell-marker-antibody-panel_sc017
BrdU:
https://www.thermofisher.com/antibody/product/BrdU-Antibody-clone-MoBU-1-Monoclonal/B35128
Goat anti-Mouse IgG (H+L) Cross-Adsorbed Secondary Antibody:
https://www.thermofisher.com/antibody/product/Goat-anti-Mouse-IgG-H-L-Cross-Adsorbed-Secondary-Antibody-Polyclonal/F-2761
Goat anti-Rabbit IgG (H+L) Secondary Antibody:
https://www.thermofisher.com/antibody/product/Goat-anti-Rabbit-IgG-H-L-Secondary-Antibody-Polyclonal/65-6111
Goat anti-Rat IgG (H+L) Cross-Adsorbed Secondary Antibody:
https://www.thermofisher.com/antibody/product/Goat-anti-Rat-IgG-H-L-Cross-Adsorbed-Secondary-Antibody-Polyclonal/A-21434

Eukaryotic cell lines

Policy information about cell lines and Sex and Gender in Research

Cell line source(s)

HDF-a is purchased from ATCC. HUVEC is purchased from Lonza. Primary ADSC is derived from discarded donor adipose tissue that obtained from The Specimen Procurement Service Center, Carle Health, Carle Foundation Hospital. A description of the commercially available cell lines and primary ADSCs is provided in the matherial and methods section.

Authentication

Primary ADSCs cell line was tested by typical morphology and flow cytometry to be CD90, CD105, CD44 positive and CD106, CD45, CD19 negative. HDF-a and HUVEC cell lines were authenticated by STR profiling.

Mycoplasma contamination

All cell lines are negative for mycoplasma contamination.

Commonly misidentified lines (See ICLAC register)	No commonly misidentified cell lines were used in this study.

Animals and other research organisms

Policy information about studies involving animals; ARRIVE guidelines recommended for reporting animal research, and Sex and Gender in Research

Laboratory animals	Sprague-Dawley rats (8 weeks old) were obtained from Envigo Laboratory (Indianapolis, IN).
Wild animals	No wild animal was used in this study.
Reporting on sex	Male mice were used in this study because previous studies show a female advantage in healing rates (Brain, behavior, and immunity, 23(5), 629-635.). To eliminate the gender effect in wound healing, we choose male rats to conduct the study.
Field-collected samples	No samples collected from the field were involved in this study.
Ethics oversight	All animal studies were performed in accordance with the guidelines of the Institutional Animal Care and Use Committee and the Division of Animal Resources at the University of Illinois (Protocol#: 23012).

Note that full information on the approval of the study protocol must also be provided in the manuscript.

Plants

Seed stocks	N/A
Novel plant genotypes	N/A
Authentication	N/A

Flow Cytometry

Plots

Confirm that:

- [x] The axis labels state the marker and fluorochrome used (e.g. CD4-FITC).
- [x] The axis scales are clearly visible. Include numbers along axes only for bottom left plot of group (a 'group' is an analysis of identical markers).
- [x] All plots are contour plots with outliers or pseudocolor plots.
- [x] A numerical value for number of cells or percentage (with statistics) is provided.

Methodology

Sample preparation	Treated cells were washed and stained with fluorescent probe or antibodies following the manufacturer's instructions.
Instrument	BD LSRFortessa
Software	BD FACSDiva 9.0 and Flowjo V10.8
Cell population abundance	10,000 cells were counted and analyzed in all experiments.
Gating strategy	FSC-SSC and singlets were pre-gated. All debris was excluded.

- [x] Tick this box to confirm that a figure exemplifying the gating strategy is provided in the Supplementary Information.
