# Supplementary material for: Exosome-coated oxygen nanobubble-laden hydrogel augments intracellular delivery of exosomes for enhanced wound healing
Source: Nat Commun. 2024 Apr 23;15:3435. doi: 10.1038/s41467-024-47696-5 (PMC11039765; doi:10.1038/s41467-024-47696-5)
Supplement: Supplementary file 2 — Peer Review File [file 41467_2024_47696_MOESM2_ESM.pdf]

nature portfolio

Peer Review File

Exosome-coated Oxygen Nanobubble-laden Hydrogel Augments Intracellular Delivery for Enhanced Wound Healing

Open Access This file is licensed under a Creative Commons Attribution 4.0 International License, which permits use, sharing, adaptation, distribution and reproduction in any medium or format, as long as you give appropriate credit to the original author(s) and the source, provide a link to the Creative Commons license, and indicate if changes were made. In the cases where the authors are anonymous, such as is the case for the reports of anonymous peer reviewers, author attribution should be to 'Anonymous Referee' followed by a clear attribution to the source work. The images or other third party material in this file are included in the article's Creative Commons license, unless indicated otherwise in a credit line to the material. If material is not included in the article's Creative Commons license and your intended use is not permitted by statutory regulation or exceeds the permitted use, you will need to obtain permission directly from the copyright holder. To view a copy of this license, visit http://creativecommons.org/licenses/by/4.0/.

REVIEWER COMMENTS

Reviewer #1 (Remarks to the Author):

In this manuscript, the authors reported an oxygen-loaded nanobubble loaded with exosomes and loaded into PVA/gelatin hydrogel for wound repair. The authors confirmed the oxygen-releasing capacity of the hydrogel and evaluated its pro-cell migration and vascularization effects through in vitro experiments. However, this manuscript need to be major revised:

1. The in vitro and in vivo degradation properties of the hydrogels need to be confirmed. In addition, does the accumulation of borax during the hydrogel degradation process cause a biosafety hazard? Please provide reasonable confirmation.

2. The manuscript stated that ADSCs-derived exosomes can achieve antioxidant effects. Please provide reasonable confirmation for this statement.

3. In what way is oxygen released from the hydrogel? Is the oxygen release process controllable? Additionally, the oxygen loading needs to be confirmed. From the oxygen release results in the manuscript, we know that oxygen release tends to decrease after 10 hours. This does not seem to be a continuous process. Can this meet the needs of wound repair?

4. For hemostatic testing of hydrogels, comparisons with commercial hemostatic products are needed to highlight the advantages of hydrogels in hemostasis.

5. What are the specific mechanisms by which hydrogels promote cell migration and vascularization? Although it is possible that exosome-secreted cytokines play a key role, this is worth exploring. The manuscript is full of descriptions of experimental conclusions and lacks output of scientific questions.

6. Chronic wounds such as diabetic wounds seem to be more suitable for the application of hydrogels due to their ability to supply oxygen, promote cell migration and promote vascularization. Why did the author not choose diabetic wounds to verify the wound repair of hydrogel?

7. The degree of vascularization of the regenerated tissue needs to be confirmed.

Reviewer #2 (Remarks to the Author):

Comments to the Authors

The paper entitled “Exosome-coated Oxygen Nanobubble-laden Hydrogel Augments Intracellular Exosome Delivery to Mitigate Hypoxia for Enhanced Wound Healing” aimed to highlight the delayed wound healing due to chronic hypoxia and inadequate angiogenesis. It specifically provided updates on the coating of oxygen nanobubbles with exosomes and incorporating them into a polyvinyl alcohol (PVA)/gelatin hybrid hydrogel for accelerating the wound healing process. The topic itself is of great interest but the manuscript needs some improvements before it can be published:

General comments:

1. The author should mention the content of the recent paper published in the Introduction section for getting the recent updates worked in this topic.

2. Figures mentioned in the research paper could have been more precise.

Specific comments:

1. The author should mention the data published after 2020 to show the recent advancements committed in this field.

2. The author has not mentioned the clear statistics or ratio of people suffering from delayed wound healing.

3. In Figure 2, the peaks of IR spectra could have been clearer and sharper.

4. Line 48 to 52 is a very long sentence, and it could have been divided into 2 or more sentences having proper meaning.

5. Lines 156 to 159 are very long sentences, and they could also have been divided into 2 or more sentences giving proper meaning.

6. Data on drug release factors are missing in this article, kindly check it and emphasize this content in your paper.

7. There are several statements given without references! This should be avoided as well as avoid giving your own opinion (without references)

All over: The manuscript is well written and provides in-depth information regarding this usage of a novel strategy for accelerating wound healing. As I suggested above, before accepting it for publication I recommend this change and would like to see the revised manuscript.

Reviewer #3 (Remarks to the Author):

In this manuscript, Han et al developed an exosome-coated oxygen-laden hydrogel to alleviate hypoxia, facilitate exosome delivery, and promote wound healing. Overall, the design of the project is effective and the manuscript is well-organized. However, there are some deficiencies that need to be improved. In the method session, a more detailed description of the experimental procedures should be provided. Some of the conclusions are not adequately supported by current data. Further analyses and characterizations are needed to validate the design and function of the material. Below are the comments and suggested changes.

1. Figure 1: Authors should provide the chemical reaction of the crosslinking process and evidence of successful boron ester formation in the hydrogel.

2. Line 117: Further information is required regarding the longevity of the core-shell structure of EBO.

3. Figure 2f: a 3D or Z-stack image with orthogonal views is required to show particles are internalized in the cell, not on the cell membrane.

4. Figure 3: The EBO-Gel needs to be further characterized to prove that it is suitable for in vivo application. It is recommended to provide the exact gelation time and injectability. To effectively evaluate the injectability of hydrogel precursors, it is essential to include shear-thinning data sets that involve stress-relaxation, viscosity, or injection force measurements.

5. Figure 3i, Need to include a high-magnification image of the blank gel for comparison. Due to the small size of exosomes, their presence is challenging to discern, and a control image would help in visual differentiation.

6. Figure 3e, incorporating cyclic strain data would provide a more comprehensive representation of the self-healing properties of the hydrogel, demonstrating continuous property changes over time.

7. Figure 6d: For the in vitro hemostatic characterization, a non-hemostatic hydrogel is needed as a control group to prove that the coagulation is due to gelation/PVA, not because the gelation holds and retains the blood cells or whole blood.

8. Line 302: In the in vivo experiment, did the author mix the precursor with borax before injection or inject them separately? What is the volume of hydrogel applied to the wounds? Considering that the hydrogel volumes applied may vary over time due to different wound closure, it is imperative to explain how this variation was controlled.

9. Figure 8: From the wound closure results, the most significant differences between groups can be found between day 2 and day 6. Such a time window is also the crucial inflammatory/regenerative stage in wound healing. However, the authors show all histological analyses on day 14, where wounds in all groups have closed. It is recommended that the authors provide histological analysis at an early-middle time point.

10. Figure 9: The authors claim that EBO-Gel promotes angiogenesis while reducing inflammation and oxidative stress. However, H&E and trichrome staining are insufficient to substantiate claims regarding infection, inflammation (referenced in line 307), and angiogenesis. Data on immune cells and blood vessels are necessary to provide conclusive evidence.

11. Line 457: In all in vitro experiments, the authors use 3% O2 as their hypoxic condition. Is this concentration physiologically related to healthy and wounded dermis? It is suggested that the authors discuss how the extent of hypoxia affects wound healing, since many studies reported that hypoxia is a stimulating factor in tissue regeneration.

12. Line 507: Please explain why the hydrogel needs to be applied to the wound every two days. Are the hydrogels fully degraded within 2 days? Were old hydrogels removed before applying new ones? Detailed information on this aspect is needed in the experiment section.

13. The manuscript should elucidate the mechanisms by which EBO-Gel treatment facilitates scarless healing.

Point-by-Point Response to Reviewers

We thank the reviewers for the valuable feedback on our manuscript **NCOMMS-23-48068** entitled "**Exosome-coated Oxygen Nanobubble-laden Hydrogel Augments Intracellular Exosome Delivery to Mitigate Hypoxia for Enhanced Wound Healing**." After carefully revising, we provided our point-by-point response as follows.

In our point-by-point response below, we use the following conventions:

- Reviewer comments are typeset in normal font.
- Our response is typeset in blue.
- *Descriptions of changes made to the manuscript are typeset in blue italics,* with references to new or revised text in the revised manuscript typeset in red. The new figures and references are highlighted in yellow.
- *Related references are provided under each response in this Reply document.*

Reply to Comments from Reviewer #1

In this manuscript, the authors reported an oxygen-loaded nanobubble loaded with exosomes and loaded into PVA/gelatin hydrogel for wound repair. The authors confirmed the oxygen-releasing capacity of the hydrogel and evaluated its pro-cell migration and vascularization effects through in vitro experiments. However, this manuscript need to be major revised:

RESPONSE: We sincerely appreciate the interest and opportunity to revise. We have expanded our approach and validated our concepts with additional in vivo experiments. We are confident that our revision with additional results provides further support for our findings and addresses the questions raised by the reviewers. We are thankful for the critique and constructive feedback that contributed to the overall quality and clarity of our work.

Comment 1. The in vitro and in vivo degradation properties of the hydrogels need to be confirmed. In addition, does the accumulation of borax during the hydrogel degradation process cause a biosafety hazard? Please provide reasonable confirmation.

RESPONSE: Thank you for this insightful suggestion. We concur with the question on affirming the degradability of adaptive hydrogel, since deficiencies could potentially hinder the process of wound closure, especially if the hydrogel does not degrade in the deep wound tissue. In the light of this suggestion, we have conducted both in vitro and in vivo degradation evaluation of the EBO-Gel (ADSC-derived **E**xosome coated **B**SA-based **O**xygen nanobubbles-embedded hydrogel) in the revised manuscript as have revised the text as follows.

"Effective degradation is crucial to prevent the retention of residual hydrogel in deep wounds, that could impede the healing due to its strong adhesion and tissue adaptability. In vitro degradation analysis revealed that EBO-Gel can degrade by 80% within 3 days under simulated physiological conditions (Figure S14), ensuring compatibility with the subsequent healing process and alignment with the dressing change intervals in in vivo studies." (Page 8, Line221-226)

The following was included in the Methods Section:

"For in vitro degradation analysis, EBO-Gels were submerged in a 5mL PBS solution (pH = 7.4) with lysozyme (1000 U/mL). The incubation was in a constant-temperature incubator set at 37 °C. The hydrogels were taken out for lyophilization and weighed at intervals of 0, 1, 2, and 3 days. To maintain enzyme functionality, the lysozyme solution was refreshed daily. The in vitro degradation rate was determined using the formula:

*Degradation rate = [(W0-Wt)/W0] * 100%*

Where W0 and Wt represents the weights of the original and remaining hydrogels, respectively." (SI: Page 4-5, Line 129-135)

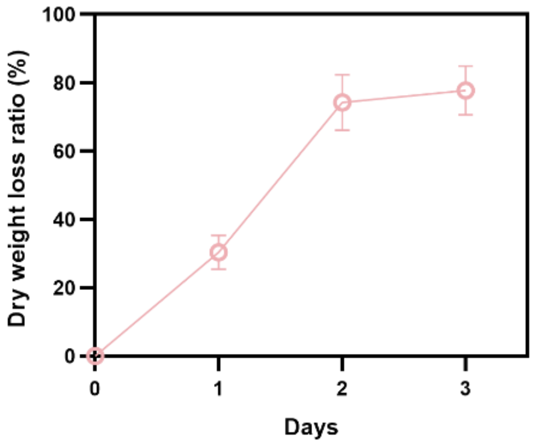

Figure S14. In vitro degradation rate of EBO-Gel within 3 days in PBS at 37 °C. (n = 3, mean ± SD)

"Effective wound closure could be potentially hindered if the adaptive hydrogel fails to degrade adequately in deep wounds, underscoring the crucial importance of its degradability[51]. For in vivo degradation assessment, the visual observation of EBO-Gel reveals a progressive reduction in size and complete degradation within a 3-day period (Figure S28). H&E staining of tissues surrounding the injection sites on Day 3 post-injection revealed no discernible inflammation induced by the injection compared to normal skin and subcutis tissues (Figure S29). This suggests the in vivo biocompatibility of EBO-Gel. Autolysis characterizes the degradation of EBO-Gel, where water molecules, with unshared electron pairs, initiate the hydrolysis of boron atoms[51, 52]. This hydrolysis causes the gradual breakdown of the borax bond crosslinked network, weakening both borax and hydrogen bond crosslinks. Consequently, the hydrogel network progressively loosens when exposed to a water environment over time, ultimately resulting in the complete dissolution of the hydrogel. To comprehensively assess the biosafety of EBO-Gel, we conducted H&E staining on visceral slices obtained from animals treated for a 14-day period (Figure S30). The results revealed no obvious systemic toxicity in animals treated with EBO-Gel compared to untreated animals, validating the biosafety of EBO-Gel." (Page 17, Line 423-436)

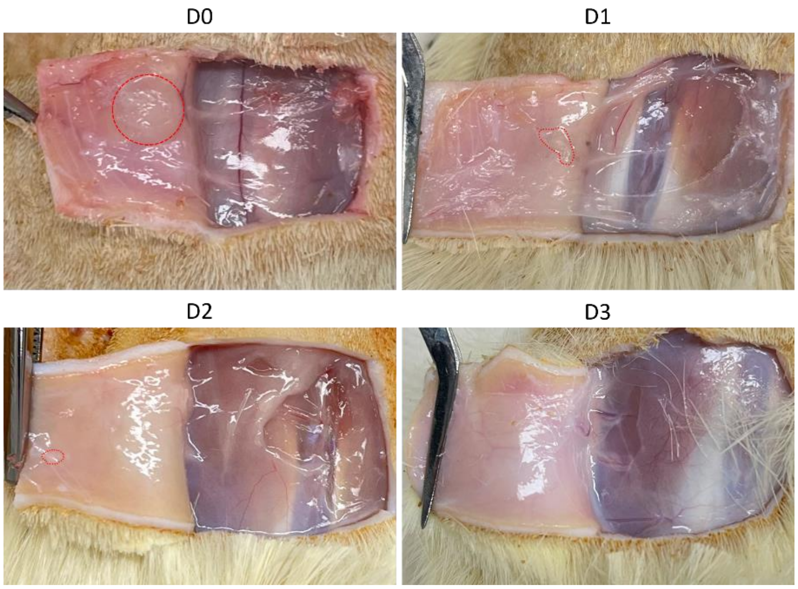

Figure S28. Overall observation of in vivo degradation of EBO-Gel within 3 days. (Residual gel is indicated by the red area)

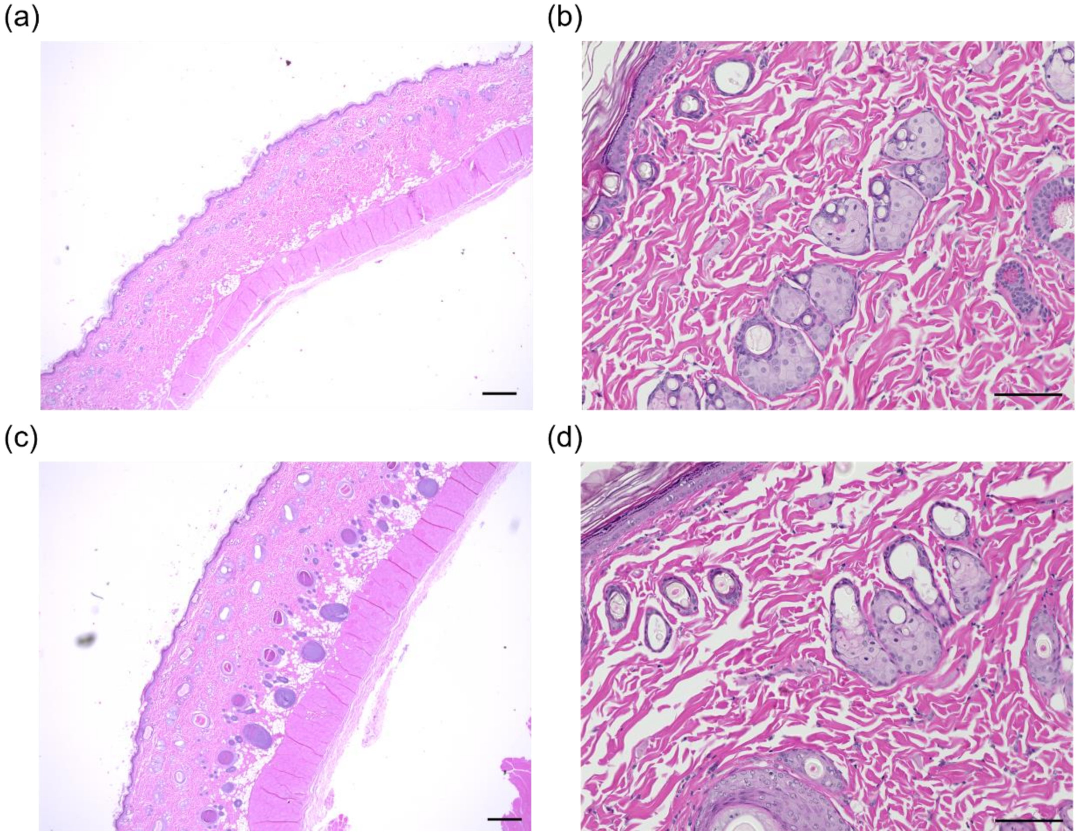

Figure S29. H&E staining of the skin and subcutis tissues of (a) and (b) normal group, (c) and (d) EBO-Gel injected group on Day 3 post-injection. Scale bar = 500 μm (a and c), 100 μm (b and d).

We agree that biosafety implications of excess borax should be addressed and appreciate this comment. In our hydrogel preparation protocol, we have taken proactive measures to address this concern by washing the hydrogels with deionized water before utilization, to effectively remove any surplus borax. Additionally, we would like to emphasize that existing research has further demonstrated the safety of PVA/borax hydrogel wound dressings based on the borate ester bond in animal models (1,2).

It is pertinent to note that the borax concentration employed in our study is 2 wt%, a level lower than the 5 wt% reported in a related work (2). To further address this concern and ensure transparency, we evaluated the in vivo safety of our EBO-Gel. The results were incorporated in the revised manuscript (SI, Page 24, Figure S30), specifically in the form of visceral slices obtained from animals subjected to treatment for a period of 14 days, as outlined below.

Page 17, Line 433-436:

"To comprehensively assess the biosafety of EBO-Gel, we conducted H&E staining on visceral slices obtained from animals treated for a 14-day period (Figure S30). The results revealed no obvious systemic toxicity in animals treated with EBO-Gel compared to untreated animals, validating the biosafety of EBO-Gel."

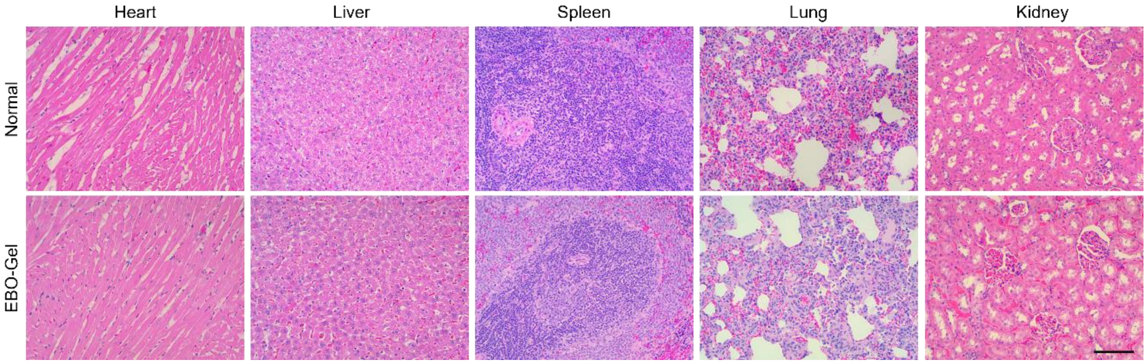

Figure S30. Images of H&E-stained major organs collected from unwounded healthy rat and EBO-Gel treated rat on day 14 post-surgery. Scale bar = 100 μm.

References

(1) Chen, M., Wu, Y., Chen, B., Tucker, A. M., Jagota, A., & Yang, S. (2022). Fast, strong, and reversible adhesives with dynamic covalent bonds for potential use in wound dressing. Proceedings of the National Academy of Sciences, 119(29), e2203074119.

(2) Zhang, H., Li, W. X., Tang, S., Chen, Y., Lan, L. M., Li, S., ... & Jiang, G. B. (2023). A Boron‐Based Probe Driven Theranostic Hydrogel Dressing for Visual Monitoring and Matching Chronic Wound Healing. Advanced Functional Materials, 33(51), 2305580.

Comment 2. The manuscript stated that ADSCs-derived exosomes can achieve antioxidant effects. Please provide reasonable confirmation for this statement.

RESPONSE: ADSC-derived exosomes (ADSCs-Exos) have been utilized in prior works to alleviate oxidative stress-induced damage across a variety of conditions, including organ damage due to sepsis inflammation (1) and reduced heart function post-myocardial infarction (2). Notably, the antioxidant effect of ADSC exosomes has been noted in prior wound healing studies (3), which provides a strong justification for their use in our wound dressing experiments.

ADSCs-Exos exhibit anti-inflammatory effects by modulating cellular responses to oxidative stress. Studies reveal that ADSCs-Exos secrete eHSP90, mitigating hypoxia and oxidative stress damage and indirectly suppressing inflammation (4). Furthermore, the high expression level of transcription factor Nrf2 in ADSCs-exo contributes to the protective effects against oxidative stress and inflammation by inhibiting the expression of inflammatory proteins and reactive oxygen species (ROS) production in wound healing (5). This mechanism involves the modulation of oxidative stress responses, positioning ADSC-derived exosomes as promising anti-inflammatory agents for wound healing applications.

Related references provided below are listed in the revised manuscript (Page 9, Line 249, Ref #37).

References

(1) Shen, K. et al. Exosomes from adipose-derived stem cells alleviate the inflammation and oxidative stress via regulating Nrf2/HO-1 axis in macrophages. Free Radical Biology and Medicine 165, 54–66 (2021).

(2) Xu, H., Wang, Z., Liu, L., Zhang, B. & Li, B. Exosomes derived from adipose tissue, bone marrow, and umbilical cord blood for cardioprotection after myocardial infarction. J of Cellular Biochemistry 121, 2089–2102 (2020).

(3) Song, Y., You, Y., Xu, X., Lu, J., Huang, X., Zhang, J., ... & Du, Y. (2023). Adipose‐Derived Mesenchymal Stem Cell‐Derived Exosomes Biopotentiated Extracellular Matrix Hydrogels Accelerate Diabetic Wound Healing and Skin Regeneration. Advanced Science, 10(30), 2304023.

(4) Ren, S., Chen, J., Guo, J., Liu, Y., Xiong, H., Jing, B., ... & Chen, Z. (2022). Exosomes from adipose stem cells promote diabetic wound healing through the eHSP90/LRP1/AKT Axis. Cells, 11(20), 3229.

(5) Li, X., Xie, X., Lian, W., Shi, R., Han, S., Zhang, H., ... & Li, M. (2018). Exosomes from adipose-derived stem cells overexpressing Nrf2 accelerate cutaneous wound healing by promoting vascularization in a diabetic foot ulcer rat model. Experimental & molecular medicine, 50(4), 1-14.

Comment 3. In what way is oxygen released from the hydrogel? Is the oxygen release process controllable? Additionally, the oxygen loading needs to be confirmed. From the oxygen release results in the manuscript, we know that oxygen release tends to decrease after 10 hours. This does not seem to be a continuous process. Can this meet the needs of wound repair?

RESPONSE: This is an excellent comment. The EBO-Gel supplies oxygen through the release of oxygen from nanobubbles embedded in the hydrogel. Previous studies have demonstrated that oxygen nanobubbles generated from ultrasound-induced cavitation have the potential to release oxygen due to their higher internal pressure than the surrounding environment (1). These bubbles enhance the solubility of their internal oxygen gas core into the liquid, thereby increasing the dissolved oxygen (DO) concentration (1-3). The transport process from the bubble to the bulk fluid can be characterized in three steps (4): **(i)** diffusion

from the bulk gas to the inner surface of the particle monolayer shell, **(ii)** diffusion through the shell and absorption into the liquid phase, and **(iii)** diffusion in the liquid from the bubble surface to the bulk.

Studies have shown that in a hypoxic environment, oxygen gas diffuses out of the bubbles, typically occurring within 2 hours (5, 6). To confirm oxygen loading, we compared the oxygen release profiles of EBO-Gel with a Control-Gel prepared by mixing PVA/GA solution with an equivalent oxygenated Exo/BSA/Dex mixture without ultrasonication. Results indicated that the maximum oxygen release concentration of EBO-Gel is ~8.4 mg/L, compared to ~4.9 mg/L for the Control-Gel, confirming oxygen loading in the EBO nanoparticle.

Additionally, we extended the oxygen release monitoring to 48 hours, to align with our dressing-refreshing interval in animal studies. While the oxygen release rate decreased after 12 hours, the DO concentration in the hypoxic environment remains higher than in the Control-Gel until 40 hours. This supports our experimental approach to refreshing dressings every 2 days to maintain optimal oxygen concentration in the wound bed.

We are grateful for this comment and have provided the following clarification in the revised manuscript (Page 9, Line 239-243):

"Extended oxygen release profiles were compared with a Control-Gel formed without ultrasonication. Despite a decline after 12 hours, the dissolved oxygen in the hypoxic environment exceeded that in Control-Gel by 40 hours (Figure S15). This supports our treatment of changing dressings every 2 days in subsequent in vivo experiments to sustain optimal oxygen levels in the wound bed."

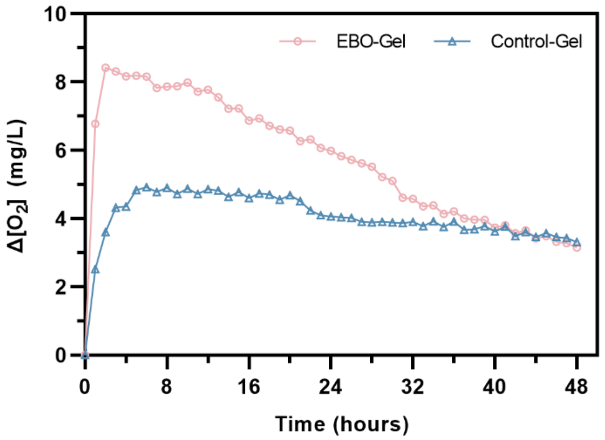

Figure S15. Oxygen concentration profile of EBO-Gel and Control-Gel (Blank-Gel with oxygenated exo/BSA/Dex solution) in 48 hrs.

References

(1) Kheir, J. N., Scharp, L. A., Borden, M. A., Swanson, E. J., Loxley, A., Reese, J. H., ... & McGowan Jr, F. X. (2012). Oxygen gas–filled microparticles provide intravenous oxygen delivery. Science translational medicine, 4(140), 140ra88-140ra88.

(2) Sayadi, L. R., Banyard, D. A., Ziegler, M. E., Obagi, Z., Prussak, J., Klopfer, M. J., ... & Widgerow, A. D. (2018). Topical oxygen therapy & micro/nanobubbles: a new modality for tissue oxygen delivery. International wound journal, 15(3), 363-374.

(3) Knowles, H. J., Vasilyeva, A., Sheth, M., Pattinson, O., May, J., Rumney, R., ... & Stride, E. (2023). Use of oxygen-loaded nanobubbles to improve tissue oxygenation: Bone-relevant mechanisms of action and effects on osteoclast differentiation. Biomaterials, 122448.

(4) Feshitan, J. A., Legband, N. D., Borden, M. A., & Terry, B. S. (2014). Systemic oxygen delivery by peritoneal perfusion of oxygen microbubbles. Biomaterials, 35(9), 2600-2606.

(5) Khan, M. S., Hwang, J., Seo, Y., Shin, K., Lee, K., Park, C., ... & Choi, J. (2018). Engineering oxygen nanobubbles for the effective reversal of hypoxia. Artificial cells, nanomedicine, and biotechnology, 46(sup3), 318-327.

(6) Messerschmidt, V., Ren, W., Tsipursky, M., & Irudayaraj, J. (2023). Characterization of Oxygen Nanobubbles and In Vitro Evaluation of Retinal Cells in Hypoxia. Translational Vision Science & Technology, 12(2), 16-16.

Comment 4. For hemostatic testing of hydrogels, comparisons with commercial hemostatic products are needed to highlight the advantages of hydrogels in hemostasis.

RESPONSE: As suggested, we have conducted additional in vitro and in vivo hemostasis assessments to compare the efficacy of the developed EBO-Gel with two commercially available hemostatic products, CURAD® BloodStop® Hemostatic Gauze and BleedStop™. The results underscore the hemostatic advantages of EBO-Gel, showcasing either parity or lower blood loss compared to CURAD® and BleedStop™, respectively. To ascertain that the observed hemostatic features are attributed to the coagulative properties of PVA/GA, an additional Carbopol gel group, representing a non-hemostatic hydrogel, was introduced in our experiments. The results consistently demonstrate that Carbopol gel, akin to the control PVA gel, manifests analogous blood loss, affirming the inherent hemostatic characteristics of EBO-Gel. A comprehensive discussion of these outcomes is included in the revised manuscript and is also presented here for your review:

"This underscores its hemostatic capability, similar to commercial products including CURAD® BloodStop® Hemostatic Gauze and BleedStop™, in comparison to both the control group and a non-hemostatic gel (Carbopol hydrogel group) (Figure 6d and Figure S16). In the rat liver hemorrhage model, the bleeding site was promptly sealed upon application of EBO-Gel, leading to a substantial reduction in blood loss, aligned with the in vitro findings (Figure 6e, 6f, S17, S18). Our results highlight the effective hemostatic performance of EBO-Gel, in addition to wound healing characteristics." (Page 12, Line 306-312)

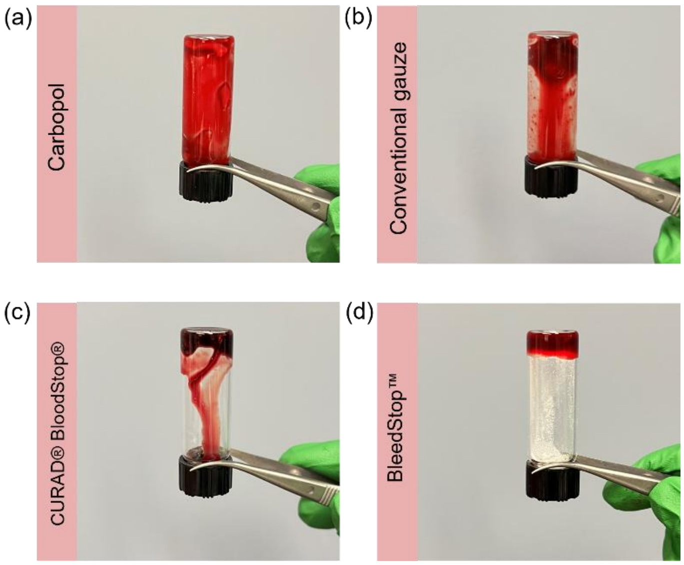

Figure S16. In vitro procoagulant effects of different treatments including (a) A non-hemostatic hydrogel, Carbopol; (b) Conventional gauze; (c) CURAD® BloodStop® Hemostatic Gauze; and (d) BleedStop™.

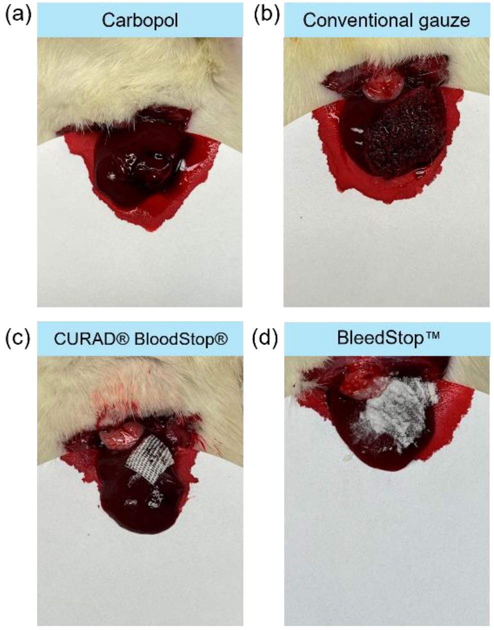

Figure S17. In vivo hemostasis evaluation of different treatments including (a) A non-hemostatic hydrogel, Carbopol; (b) Conventional gauze; (c) CURAD® BloodStop® Hemostatic Gauze; and (d) BleedStop™.

Comment 5. What are the specific mechanisms by which hydrogels promote cell migration and vascularization? Although it is possible that exosome-secreted cytokines play a key role, this is worth exploring. The manuscript is full of descriptions of experimental conclusions and lacks output of scientific questions.

RESPONSE: This is an important comment. We agree that further clarification on the mechanisms governing how EBO-Gel promotes cell migration and angiogenesis should be provided. Consequently, we have discussed the potential mechanisms here and provided an overview of these insights in the revised manuscript (Page 13-14, Line 330-333, 341-347).

The proposed EBO-Gel promotes cell migration and vascularization due to the following mechanisms: (a) regulation of wound healing signaling pathways by the Adipose-derived stem cell-derived exosomes (ADSC-Exos) and (b) oxygen delivery.

Cell migration: ADSC-Exos have emerged as promising agents facilitating heightened migration of dermal fibroblasts, thereby expediting wound healing. In a study by Cooper et al. (1), the observed pro-migratory effect was shown to result from the release of a specific long non-coding RNA (lncRNA) termed MALAT1 (metastasis-associated lung adenocarcinoma transcript 1), a constituent of ADSC-Exos. MALAT1 is known for its regulatory roles in cell migration (2,3), thereby mediating the healing processes, especially in circumstances characterized by compromised oxygen and nutrient availability.

Another pivotal mechanism involves the modulation of Apoptosis Peptidase Activating Factor 1 (APAF1), a participant in the mitochondrial apoptosis pathway. The elevated APAF1 expression is associated with diminished viability and migration of HaCaT cells. ADSC-Exos, through the action of miR-93-3p contained within these exosomes, actively deactivate APAF1 (4). This miRNA-mediated regulatory process serves to restore cellular functionality and hinder apoptosis in epithelial HaCaT cells, contributing significantly to the enhancement of cellular viability and migration during wound healing.

Angiogenesis: A foundational prerequisite for neovascularization is the establishment of an extracellular matrix (ECM) endowed with an optimal oxygen supply (5,6). Collagen deposition, a fundamental aspect of the ECM, is vital for aortic endothelial cell migration. However, poor fibroblast production of collagen, an oxygen-dependent process, hinders adequate ECM production. The molecular oxygen is indispensable for the hydroxylation of proline and lysine during collagen synthesis, concomitant with the maturation of protocollagen into the structurally resilient triple-helical collagen (7). In instances of oxygen scarcity, the production is confined to protocollagen, which lacks the tensile strength characteristic of mature collagen. This compromise in collagen dynamics impedes endothelial migration, which impairs blood vessel formation.

Additionally, studies emphasize the crucial role of ADSCs in promoting angiogenesis, primarily through paracrine exosomes. Key regulators within ADSC-Exos, such as vascular endothelial growth factor A (VEGFA), the transcription factor nuclear factor-E2-related factor 2 (Nrf2) and fibroblast growth factor 2 (FGF2), play pivotal roles in angiogenic processes (8). Moreover, β-catenin activation induced by ADSC-Exos further amplifies proangiogenic effects, contributing to improved cutaneous wound healing (9). Furthermore, ADSC-Exos harbor microRNAs, such as miR-31 and miR-125a, which play crucial roles in promoting angiogenesis by modulating various signaling pathways, including the suppression of the angiogenic inhibitor delta-like 4 (DLL4) expression (8). These multifaceted mechanisms collectively contribute to the proangiogenic effects of ADSC-derived exosomes in endothelial cells.

We have provided an overview of these insights with references in the manuscript and revised the text as follows.

Page 13-14, Line 330-333:

"ADSC-Exo plays a crucial role in promoting dermal fibroblast migration and wound healing by releasing MALAT1, a specific long non-coding RNA[43]. Additionally, the modulation of Apoptosis Peptidase Activating Factor 1 (APAF1) through miR-93-3p in ADSC-Exos contributes to improved cellular viability and migration, particularly in hypoxic conditions[44]."

Page 14, Line 341-347:

"The establishment of an extracellular matrix (ECM) is vital for neovascularization, with collagen deposition playing a fundamental role in aortic endothelial cell migration, which is an oxygen-dependent process[46]. Hence, oxygen supply might be a potential strategy for enhancing blood vessel formation. Moreover, EBO aids angiogenesis through exosomes containing regulators such as Nrf2 and FGF2, with exosomes promoting β-catenin activation and harboring microRNAs (miR-31, miR-125a) that modulate angiogenic pathways, collectively enhancing proangiogenic effects[47, 48]."

References

(1) Cooper, D. R., Wang, C., Patel, R., Trujillo, A., Patel, N. A., Prather, J., ... & Wu, M. H. (2018). Human adipose-derived stem cell conditioned media and exosomes containing MALAT1 promote human dermal fibroblast migration and ischemic wound healing. Advances in wound care, 7(9), 299-308.

(2) Li, C., Wei, S., Xu, Q., Sun, Y., Ning, X., & Wang, Z. (2022). Application of ADSCs and their exosomes in scar prevention. Stem Cell Reviews and Reports, 1-16.

(3) Ying, L., Chen, Q., Wang, Y., Zhou, Z., Huang, Y., & Qiu, F. (2012). Upregulated MALAT-1 contributes to bladder cancer cell migration by inducing epithelial-to-mesenchymal transition. Molecular biosystems, 8(9), 2289-2294.

(4) Zhou, C., Zhang, B., Yang, Y., Jiang, Q., Li, T., Gong, J., ... & Zhang, Q. (2023). Stem cell-derived exosomes: emerging therapeutic opportunities for wound healing. Stem Cell Research & Therapy, 14(1), 107.

(5) Strodtbeck, F. (2001). Physiology of wound healing. Newborn and infant nursing reviews, 1(1), 43-52.

(6) Rhodes, J. M., & Simons, M. (2007). The extracellular matrix and blood vessel formation: not just a scaffold. Journal of cellular and molecular medicine, 11(2), 176-205.

(7) Hong, W. X., Hu, M. S., Esquivel, M., Liang, G. Y., Rennert, R. C., McArdle, A., ... & Longaker, M. T. (2014). The role of hypoxia-inducible factor in wound healing. Advances in wound care, 3(5), 390-399.

(8) Qiu, H., Liu, S., Wu, K., Zhao, R., Cao, L., & Wang, H. (2020). Prospective application of exosomes derived from adipose - derived stem cells in skin wound healing: A review. Journal of cosmetic dermatology, 19(3), 574-581.

(9) Rani, S., & Ritter, T. (2016). The exosome - A naturally secreted nanoparticle and its application to wound healing. Advanced materials, 28(27), 5542-5552.

Comment 6. Chronic wounds such as diabetic wounds seem to be more suitable for the application of hydrogels due to their ability to supply oxygen, promote cell migration and promote vascularization. Why did the author not choose diabetic wounds to verify the wound repair of hydrogel?

RESPONSE: We appreciate this insightful comment on the potential application of EBO-Gel in chronic wounds such as diabetic wounds.

While we acknowledge the suitability of chronic wounds as an in vivo model for testing EBO-Gel efficiency, it is important to note that EBO-Gel is also well-suited for acute surgical wound healing.

The choice to focus on acute surgical wounds is motivated by the critical global medical issue of acute wound healing failure, which poses a significant source of morbidity and mortality for surgical patients. Instances such as incisional hernias, gastrointestinal anastomotic leaks, and vascular pseudoaneurysms often occur despite patient optimization and standardized surgical techniques (1). EBO-Gel exhibits several key features making it suitable for a surgical wound healing model:

(a) Hemostasis Capacity: Clinical studies have demonstrated impaired surgical wound healing in patients after severe blood loss. The self-healing bio-adhesive properties of EBO-Gel makes it a promising application as an embolic hemostatic agent. Its ability to seamlessly adhere to the site of bleeding forms a physical seal, effectively controlling and managing the bleeding process. Gelatin, a component of EBO-Gel, has gained attention for its promising hemostatic properties, triggering platelet aggregation, and facilitating hemostasis (2).

(b) Oxygen Supply: Oxygen therapy has been proven to be effective in angiogenesis in both chronic and acute wound healing. Hopfner et al demonstrated the neovascularization potential of an oxygen-generating microalgae scaffold in mice surgical full-skin defects (3). EBO-Gel provides a means to support oxygen supply during the critical early stages of healing.

(c) ADSC-Exos: Enriched with fibrosis-regulating microRNAs such as MiR-192-5p and miR-29a, ADSC-Exos in EBO-Gel demonstrate efficiency in preventing fibrosis and hypertrophic scar formation (4). This is crucial for maintaining the normal mechanical strength of healed skin post-surgery.

We agree that application of EBO-Gel in chronic wounds, especially diabetic wound models, is a logical extension and will consider this suggestion in our future works to broaden the application of our conceived methods. We have noted this point in the revised manuscript. We thank you for this suggestion.

Page 20, Line 478-481:

"Notably, EBO-Gel could also be potentially used for diabetic chronic wound treatment, leveraging its oxygen-supplying capability, antioxidant properties, promotion of cell migration, and enhancement of vascularization. Future research can target other ischemic conditions, including chronic wounds and potentially cancer."

References

(1) Dubay, D. A., & Franz, M. G. (2003). Acute wound healing: the biology of acute wound failure. Surgical Clinics, 83(3), 463-481.

(2) Choi, Y. S., Hong, S. R., Lee, Y. M., Song, K. W., Park, M. H., & Nam, Y. S. (1999). Study on gelatin-containing artificial skin: I. Preparation and characteristics of novel gelatin-alginate sponge. Biomaterials, 20(5), 409-417.

(3) Schenck, T. L., Hopfner, U., Chávez, M. N., Machens, H. G., Somlai-Schweiger, I., Giunta, R. E., ... & Egaña, J. T. (2015). Photosynthetic biomaterials: a pathway towards autotrophic tissue engineering. Acta biomaterialia, 15, 39-47.

(4) Li, Y., Zhang, J., Shi, J., Liu, K., Wang, X., Jia, Y., ... & Hu, D. (2021). Exosomes derived from human adipose mesenchymal stem cells attenuate hypertrophic scar fibrosis by miR-192-5p/IL-17RA/Smad axis. Stem Cell Research & Therapy, 12, 1-16.

Comment 7. The degree of vascularization of the regenerated tissue needs to be confirmed.

RESPONSE: We agree and consequently have expanded the tissue CD31 immunofluorescent staining to demonstrate angiogenesis of the regenerated tissue. We now have provided results as below.

As an important biomarker of endothelial cells, the expression level of CD31 is widely evaluated in angiogenesis studies (1). As depicted in Figure 9a (Page 19) and Figure S23 (SI: Page 21), the results of immunofluorescent staining for CD31 exhibited increased expression and fluorescence intensity in the EBO-Gel treated group, highlighting the fact that EBO-Gel promotes angiogenesis.

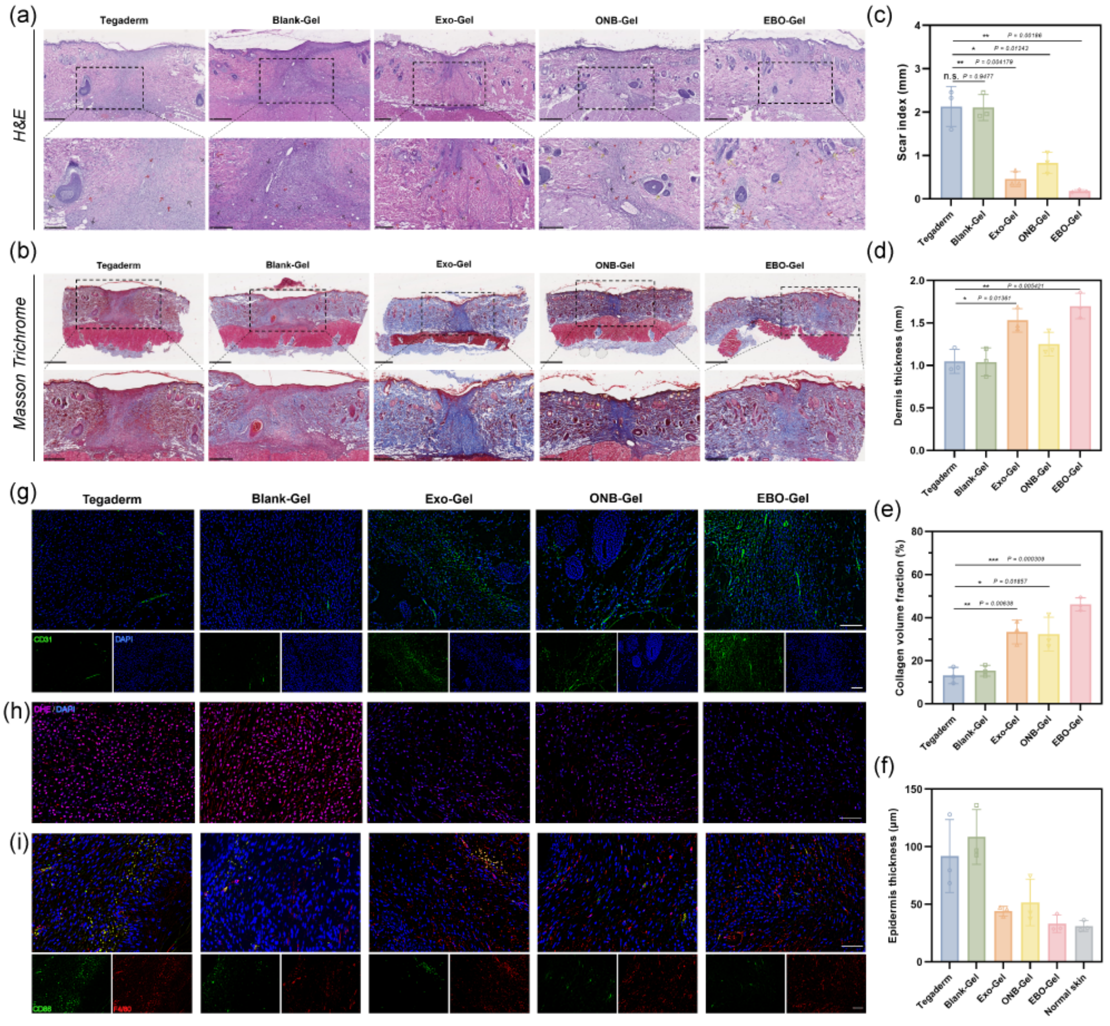

Figure 9. histological analysis of wounds that underwent treatments with different hydrogels.

Representative images of (a) H&E and (b) Masson's trichrome staining of the wound tissue on Day 14. Red arrows: new blood vessel formation; Black arrows: inflammation area; Yellow arrows: newly generated-hair follicles. Scale bar: 500 µm for the normal-sized image, and 250 µm for the magnified image. Various parameters for wound healing evaluation, including: (c) Scar index; (d) Dermis thickness; (e) Epidermis thickness; and (f) Collagen volume fraction (n = 3). Representative fluorescence images of (g) CD31 immunostaining, (h) DHE staining, and (i) CD86 and F4/80 immunostaining of wound tissues on Day 14 post-treatment. Scale bar = 100 µm (CD31) and 50 µm (DHE and CD86).

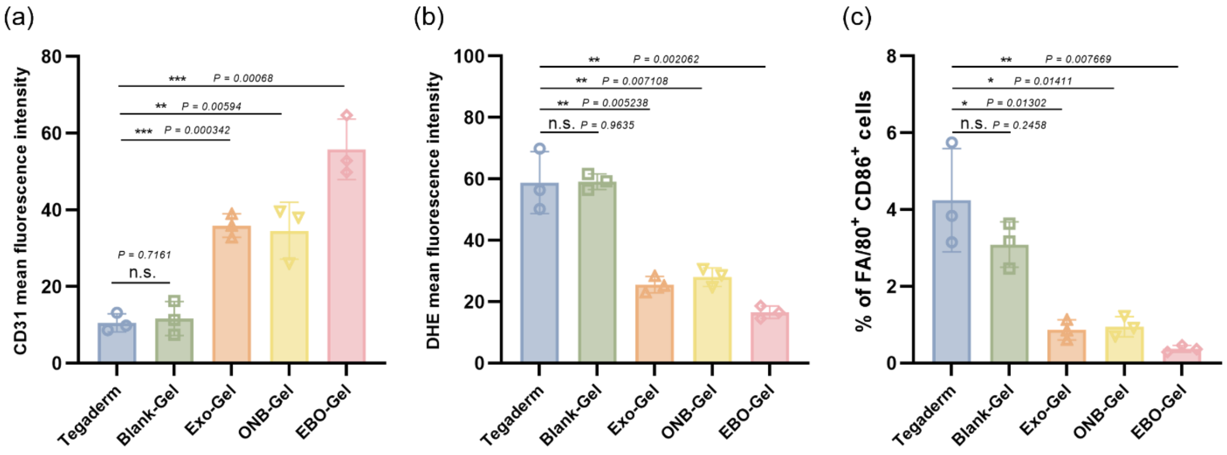

Figure S23. Quantification of fluorescent intensity of (a) CD31 and (b) DHE (n = 3, mean ± SD). (c) Quantification of the percentage of M1 macrophages (n = 3, mean ± SD).

References

[1] Pusztaszeri, Marc P., Walter Seelentag, and Fred T. Bosman. "Immunohistochemical expression of endothelial markers CD31, CD34, von Willebrand factor, and Fli-1 in normal human tissues." Journal of Histochemistry & Cytochemistry 54.4 (2006): 385-395.

Reply to Comments from Reviewer #2

Comments to the Authors

The paper entitled "Exosome-coated Oxygen Nanobubble-laden Hydrogel Augments Intracellular Exosome Delivery to Mitigate Hypoxia for Enhanced Wound Healing" aimed to highlight the delayed wound healing due to chronic hypoxia and inadequate angiogenesis. It specifically provided updates on the coating of oxygen nanobubbles with exosomes and incorporating them into a polyvinyl alcohol

(PVA)/gelatin hybrid hydrogel for accelerating the wound healing process. The topic itself is of great interest but the manuscript needs some improvements before it can be published:

RESPONSE: We are grateful for the interest and positive feedback and have carefully revised the manuscript according to the suggestions.

General comments:

Comment 1. The author should mention the content of the recent paper published in the Introduction section for getting the recent updates worked in this topic.

RESPONSE: We have reviewed the manuscript and provided an overview of the recent efforts in this field and added the relevant references. Please see our response to "Specific comments - Comment 1, 2, 4, 5, and 7"

Comment 2. Figures mentioned in the research paper could have been more precise.

RESPONSE: As suggested, we added additional results and re-plotted the figure to improve clarity. Please see the response in "Specific comments – Comment 3 and 6."

Specific comments:

Comment 1. The author should mention the data published after 2020 to show the recent advancements committed in this field.

RESPONSE: We appreciate this comment. After a thorough literature search, we have incorporated an overview and a representative reference list of exosome-laden hydrogel research in tissue regeneration post-2020 into the revised introduction section. The following text and references are now available in the revised manuscript:

Page 2, Line 54-58:

"Recent studies emphasize the potential of exosome-loaded hydrogels as multifunctional dressings for both acute and chronic wound healing[11, 12]. The data showcases their capability to mitigate oxidative stress, stimulate angiogenesis, and enhance fibroblast migration, contributing positively to all phases of the wound healing process[13, 14, 15].

References (Page 24, Ref #11-15)

11. Shang S, Zhuang K, Chen J, Zhang M, Jiang S, Li W. A bioactive composite hydrogel dressing that promotes healing of both acute and chronic diabetic skin wounds. Bioactive Materials 34, 298-310 (2024).

12. Gondaliya P, et al. Mesenchymal Stem Cell-Derived Exosomes Loaded with miR-155 Inhibitor Ameliorate Diabetic Wound Healing. Mol Pharm 19, 1294-1308 (2022).

13. Cheng P, et al. Hypoxia endothelial cells-derived exosomes facilitate diabetic wound healing through improving endothelial cell function and promoting M2 macrophages polarization. Bioactive Materials 33, 157-173 (2024).

14. Xiong Y, et al. Reactive Oxygen Species-Scavenging Nanosystems in the Treatment of Diabetic Wounds. Advanced Healthcare Materials 12, 2300779 (2023).

15. Hu S, et al. Exosome-eluting stents for vascular healing after ischaemic injury. Nature Biomedical Engineering 5, 1174-1188 (2021)."

Comment 2. The author has not mentioned the clear statistics or ratio of people suffering from delayed wound healing.

RESPONSE: Thank you for pointing out this. The requested statistical information is provided in the revised manuscript (Page 2, Line 41-43), and explained below:

Delayed wound healing affects 6.5 million patients in the US, with surgical wounds being the costliest among all wound types, contributing significantly to total Medicare spending (1,2). This high cost is primarily associated with the substantial number of inpatient and outpatient surgeries, totaling 14.4, 11.9 and 10.3 million procedures in 2018, 2019, and 2020 (3) (Data of 2019 and 2020 is from the Agency for Healthcare Research and Quality, US. https://www.ahrq.gov). Millions of surgical wounds are generated each year in routine medical procedures in the United States and Europe, emphasizing the imperative to enhance healing outcomes, minimize aesthetic impact, and restore tissue function (4). While minor injuries typically heal effectively, larger wounds or significant blood loss can impede the healing process, resulting in delays and potential development of pathological scars such as keloids and hypertrophic scars.

References

(1) Nussbaum, S. R., Carter, M. J., Fife, C. E., DaVanzo, J., Haught, R., Nusgart, M., & Cartwright, D. (2018). An economic evaluation of the impact, cost, and medicare policy implications of chronic nonhealing wounds. Value in Health, 21(1), 27-32.

(2) Sen, C. K., Gordillo, G. M., Roy, S., Kirsner, R., Lambert, L., Hunt, T. K., ... & Longaker, M. T. (2009). Human skin wounds: a major and snowballing threat to public health and the economy. Wound repair and regeneration, 17(6), 763-771.

(3) McDermott, K. W., & Liang, L. (2021). Overview of operating room procedures during inpatient stays in US hospitals, 2018.

(4) Eming, S. A., Martin, P., & Tomic-Canic, M. (2014). Wound repair and regeneration: mechanisms, signaling, and translation. Science translational medicine, 6(265), 265sr6-265sr6.

Comment 3. In Figure 2, the peaks of IR spectra could have been clearer and sharper.

RESPONSE: The Fourier-transform infrared (FT-IR) spectrum plot has been re-plotted, and peaks are noted in the revised figure below. The updated Figure 2h is now available in the revised manuscript (Page 6, Line 159) and listed below for reference:

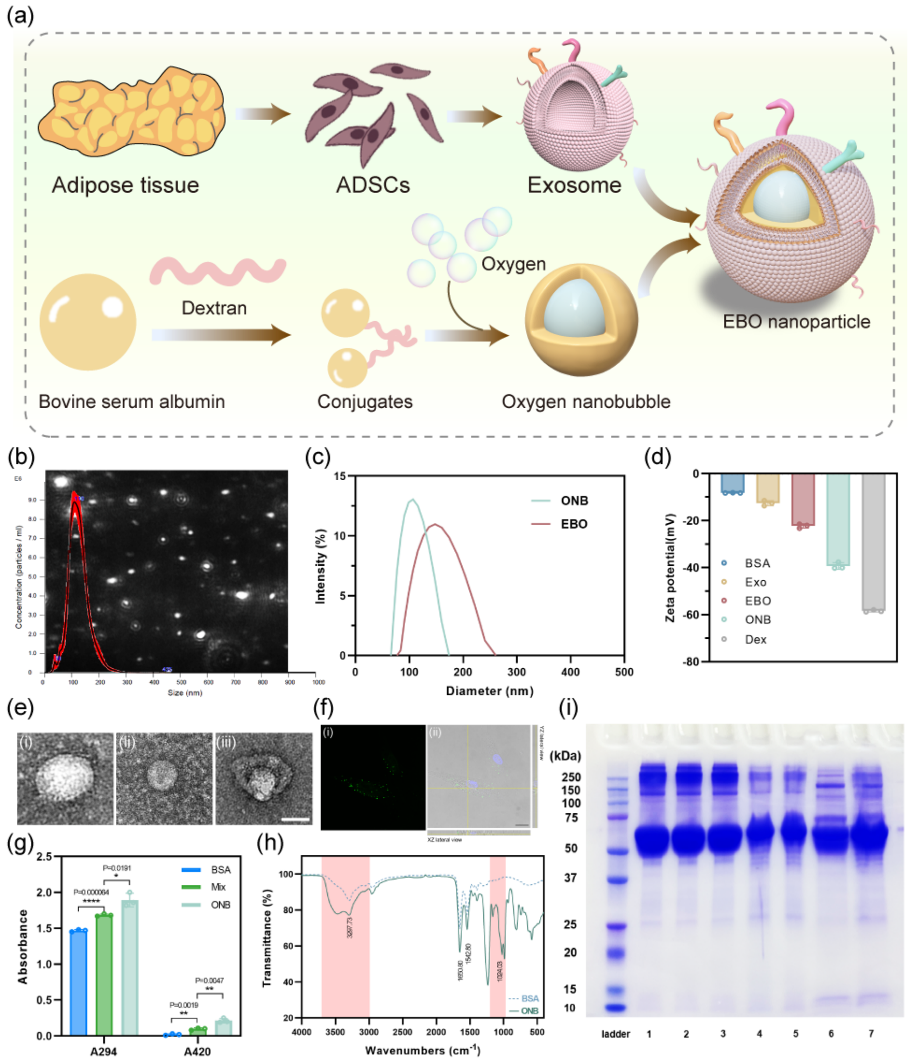

Figure 2. Synthesis and characterization of EBO.

(a) Schematic of the preparation of ONB and EBO. (b) Concentration distribution and scattered images (background) of exosomes. Diameter distribution (c) and zeta potential values (d) of ONB and EBO (n=3). (e) TEM images of (i) exosomes; (ii) ONB; (iii) EBO. Scale bar = 50 nm. (f) (i) EBO uptake and (ii) Z-stack slice with orthogonal views of EBO (Green) internalization in HDF-a cells after 6 hours of incubation. Scale bar = 10 µm. (g) The browning intensity of early (A_{294}) and late (A_{420}) MRP (n=3). (h) Infrared spectra of BSA and ONB. (i) SDS-PAGE of different formulations. Lanes: 1. Natural BSA; 2. Mixture of BSA and dextran sulfate; 3. Ultrasonicated BSA; 4. Shell; 5. ONB; 6. Exosomes; 7. EBO. Data are shown as mean ± standard deviation (SD).

Comment 4. Line 48 to 52 is a very long sentence, and it could have been divided into 2 or more sentences having proper meaning.

RESPONSE: We thank the reviewer for this suggestion. As suggested, we have divided the mentioned sentence in the revised manuscript, as detailed below. We hope that this modification enhances the readability of the manuscript.

Page 2, Line 50-54:

"Adipose-derived stem cell (ADSC)-derived exosomes have emerged as promising therapeutic agents in tissue regeneration. This is primarily attributed to their capability to function as intercellular communicators and harbor a rich cargo of bioactive molecules, including proteins, nucleic acids, and lipids. These molecules contribute to wound healing by exerting anti-inflammatory effects, inhibiting apoptosis, promoting angiogenesis, and facilitating enhanced cell migration and proliferation[9,10]."

Comment 5. Lines 156 to 159 are very long sentences, and they could also have been divided into 2 or more sentences giving proper meaning.

RESPONSE: Thank you for the suggestion. The specified sentence was edited in the updated manuscript and listed below. We hope that this modification enhances the readability of the manuscript.

Page 7, Line 186-190:

"To ascertain the shape adaptability, the hydrogel underwent iterative remodeling, wherein it was repeatedly reconfigured into various morphologies including star shape, crescent moon shape, round, and cross shape, which demonstrates the favorable capacity of EBO-Gel to conform effectively to irregular wound shapes, thus highlighting its adaptability in a clinical setting (Figure 3c)."

Comment 6. Data on drug release factors are missing in this article, kindly check it and emphasize this content in your paper.

RESPONSE: We are grateful for this input aimed at enhancing the comprehensiveness of our work. In response to this suggestion, we have examined the EBO nanoparticle release profile within a 48-hour window, to coincide with the change in dressing interval in our in vivo studies. We hope that this supplementary result will address any concerns and contribute to the improvement of the manuscript.

The revised documents now incorporate the following content:

"The EBO release profile was shown in Figure S9, indicating that over 80% of EBO is released from EBO-Gel within 48 hours. This corresponds with the intervals for refreshing wound dressings, ensuring the efficient release of nanoparticles to the wound bed before the next dressing." (Page 7, Line 183-186)

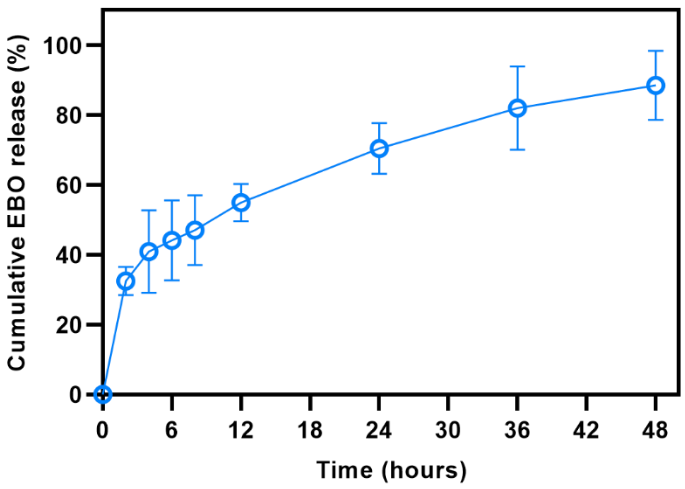

Figure S9. EBO nanoparticle release profile from EBO-Gel within 48 hrs at a pH of 7.4, 37 °C. (n = 3, mean ± SD) (SI: Page 12, Line 247-249)

Comment 7. There are several statements given without references! This should be avoided as well as avoid giving your own opinion (without references)

RESPONSE: We apologize for this oversight. We carefully revised the manuscript and added the missing relevant references. These references include:

Ref# 3, to clarify the statistics on the number of cases suffering from delayed wound healing. (See Reviewer #2, Comment 2)

Ref# 11-15, to show recent studies in this field. (See Reviewer #2, Comment 1)

Ref# 43-44, 46-48, to explain the promoted migration and angiogenesis capacity of hydrogel.

Ref# 53-58, to discuss the role of hypoxia in wound healing and the potential mechanism of scarless wound healing.

All over: The manuscript is well written and provides in-depth information regarding this usage of a novel strategy for accelerating wound healing. As I suggested above, before accepting it for publication I recommend this change and would like to see the revised manuscript.

RESPONSE: We greatly appreciate this feedback. We are hopeful that the revised manuscript provides further clarification of the methodology and discussion.

Reply to Comments from Reviewer #3

In this manuscript, Han et al developed an exosome-coated oxygen-laden hydrogel to alleviate hypoxia, facilitate exosome delivery, and promote wound healing. Overall, the design of the project is effective and the manuscript is well-organized. However, there are some deficiencies that need to be improved. In the method session, a more detailed description of the experimental procedures should be provided. Some of the conclusions are not adequately supported by current data. Further analyses and characterizations are needed to validate the design and function of the material. Below are the comments and suggested changes.

RESPONSE: We greatly appreciate the interest and positive feedback. A point-by-point response follows:

Comment 1. Figure 1: Authors should provide the chemical reaction of the crosslinking process and evidence of successful boron ester formation in the hydrogel.

RESPONSE: We agree on the importance of providing the chemical reaction involved in the crosslinking process, as it lays the foundation for the self-healing, adhesive, and biodegradable properties of EBO-Gel. Consequently, we have incorporated the reaction details (1-4) in the revised manuscript (Figure 1a, Page 3-4, Line 94-96) and list below for your review:

Page 3, Line 80-83:

"The mechanism that is responsible for self-healing ability of the PVA/GA hydrogels is the boronic crosslinks. Two possible routes were presumed that depicts intraspecies (PVA-PVA) and interspecies (PVA-GA) crosslink: (i) boronic-esters[23]; and (ii) ionic crosslinks[24] (Figure 1a)."

The evidence of successful boron-ester formation in both Blank-Gel and EBO-Gel is now presented in Figure S10 and Video S2, from which the relaxation time and viscosity of the hydrogel is significantly increased from the crosslinks. This is confirmed by our frequency sweep results that the viscosity of our hydrogel is about 2,000 Pa·s.

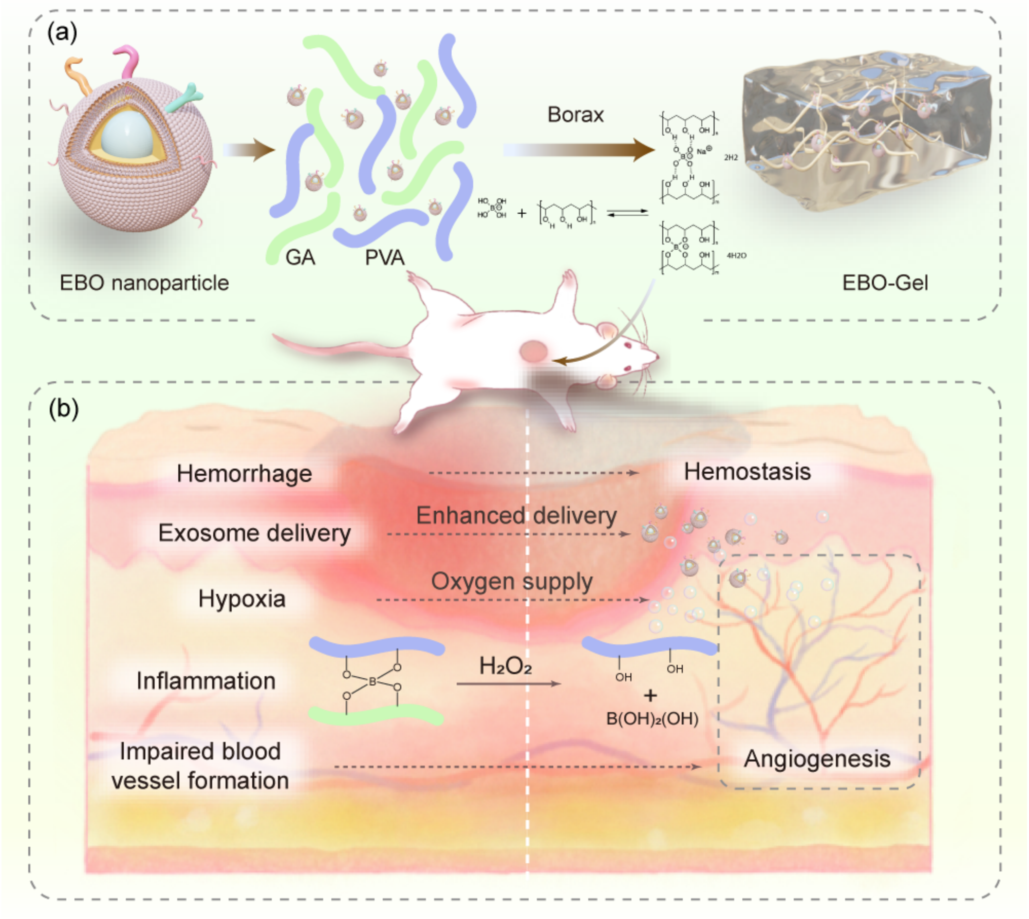

Figure1. Schematic illustration of EBO-Gel-mediated wound healing.

(a) Crosslinking mechanisms and structure of EBO-Gel. (b) Enhanced wound healing programmed by hemostasis, promoted exosome delivery, oxygen supply, angiogenesis, and antioxidant properties offered by EBO-Gel.

References

(1) Yu, H., Zhao, L., & Wang, L. (2023). Double - network PVA/gelatin/borax hydrogels with self - healing, strength, stretchable, stable, and transparent properties. Journal of Applied Polymer Science, 140(20), e53852.

(2) Liu, C., Lei, F., Li, P., Wang, K., & Jiang, J. (2021). A review on preparations, properties, and applications of cis-ortho-hydroxyl polysaccharides hydrogels crosslinked with borax. International Journal of Biological Macromolecules, 182, 1179-1191.

(3) Nijenhuis, K. T. (1997). Thermoreversible networks: viscoelastic properties and structure of gels. Advances in polymer science, 130(1).

(4) Shibayama, M., Hiroyuki, Y., Hidenobu, K., Hiroshi, F., & Shunji, N. (1988). Sol-gel transition of poly (vinyl alcohol)-borate complex. Polymer, 29(11), 2066-2071.

Comment 2. Line 117: Further information is required regarding the longevity of the core-shell structure of EBO.

RESPONSE: In the revised manuscript, we investigated the duration of the core-shell structure of EBO under different storage conditions: 2 days at 37 °C and 1 month at 4 °C. The detailed information is provided in the updated manuscript as listed below:

Page 5, Line 152-158:

"The extended duration of oxygen nanobubble presence during storage is crucial for efficient oxygen release. To evaluate the stability, particularly the longevity of the core-shell structure of EBO, TEM images were obtained under different storage conditions (2 days at 37 °C and 1 month at 4 °C). As illustrated in Figure S7, despite the dissociated protein observed in the background after 1 month at 4 °C (possibly exosome fragments with negative staining), EBO successfully maintains its core-shell structure in both conditions. This resilience is advantageous for ensuring sustained oxygen supply upon subsequent use."

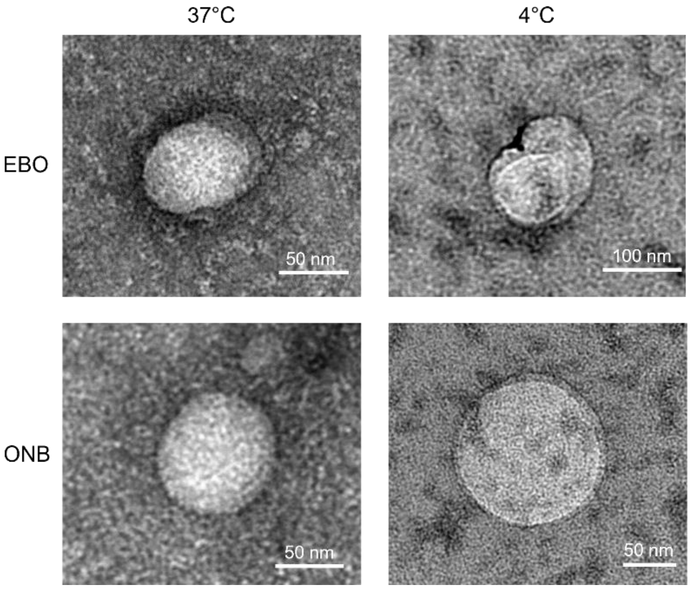

Figure S. TEM images of EBO and ONB following storage for 2 days at 37 °C or 1 month at 4 °C.

Comment 3. Figure 2f: a 3D or Z-stack image with orthogonal views is required to show particles are internalized in the cell, not on the cell membrane.

RESPONSE: Thank you for this insightful suggestion. We confirmed the EBO nanoparticle internalization in HDF-a cells using Z-stack 3D confocal imaging with orthogonal views. The orthogonal XZ and YZ sections of a Z-stack demonstrate the presence of EBOs in the cytoplasm (Figure 2f (ii) and Supplementary Video S1). The result is provided in the revised manuscript (Page 4, Line 131-133).

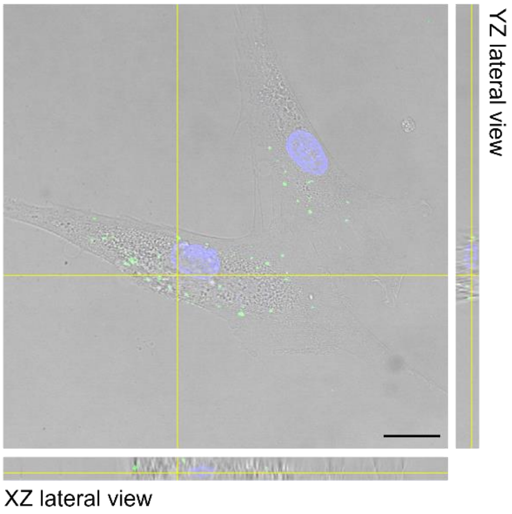

Figure 2. (f) (ii) Z-stack slice with orthogonal views of EBO (Green) internalization in HDF-a cells after 6 hours of incubation. Scale bar = 10 µm.

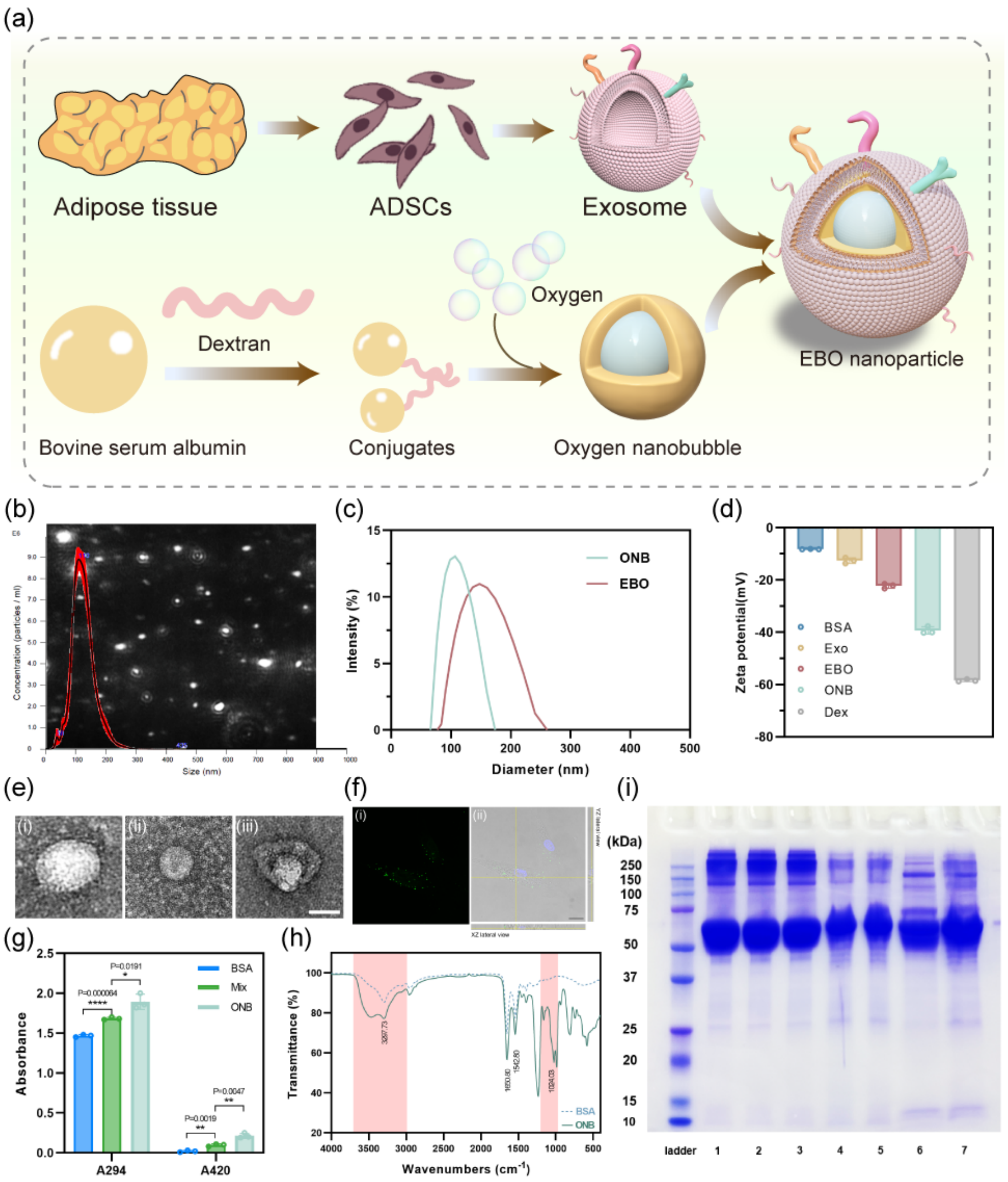

Figure 2. Synthesis and characterization of EBO.

(a) Schematic of the preparation of ONB and EBO. (b) Concentration distribution and scattered images (background) of exosomes. Diameter distribution (c) and zeta potential values (d) of ONB and EBO (n=3). (e) TEM images of (i) exosomes; (ii) ONB; (iii) EBO. Scale bar = 50 nm. (f) (i) EBO uptake and (ii) Z-stack slice with orthogonal views of EBO (Green) internalization in HDF-a cells after 6 hours of incubation. Scale bar = 10 µm. (g) The browning intensity of early (A_{294}) and late (A_{420}) MRP (n=3). (h) Infrared spectra of BSA and ONB. (i) SDS-PAGE of different formulations. Lanes: 1. Natural BSA; 2. Mixture of BSA and dextran sulfate; 3. Ultrasonicated BSA; 4. Shell; 5. ONB; 6. Exosomes; 7. EBO. Data are shown as mean ± standard deviation (SD).

Comment 4. Figure 3: The EBO-Gel needs to be further characterized to prove that it is suitable for in vivo application. It is recommended to provide the exact gelation time and injectability. To effectively evaluate the injectability of hydrogel precursors, it is essential to include shear-thinning data sets that involve stress-relaxation, viscosity, or injection force measurements.

RESPONSE: We appreciate this feedback. To provide further clarification, we have included a video depicting the gelation process and the flow curve in the revised Supplementary Information, both of which are also detailed below for your review:

The crosslinking reaction time (gelation time) is too short to measure. The main crosslinking, i.e. boronic ester, is established upon mixing. However, hydrogen bonds that crosslink gelatin-gelatin and gelatin-PVA chains continues to form, and the hydrogel modulus continues to grow as a result. The visual evidence of the boronic ester formation is now provided in Figure S10. The latter was measured from the cyclic strain test (SAOS-LAOS-SAOS), depicted by the very slow mutation, see Figure S13.

As for the evidence of shear thinning, we extracted dynamic viscosity, $|\eta^*|$, as a function of applied stress amplitude, σ_0. This curve shows extreme shear thinning properties once the applied stress exceeds the critical yield stress, σ_y, see Figure S12. The shear thinning behavior is further confirmed in Figure 3b, where the injection of the hydrogel was possible through a 1 mL syringe. Calculation of the force required to extrude the gel is provided: $F = 2\sigma_y \frac{L}{R} A = 0.8\mathrm{N}$, which falls well below an average hand strength ~360N (1). This discussion is now included in the revised Supplementary Information (SI: Page 3, Line 77-92).

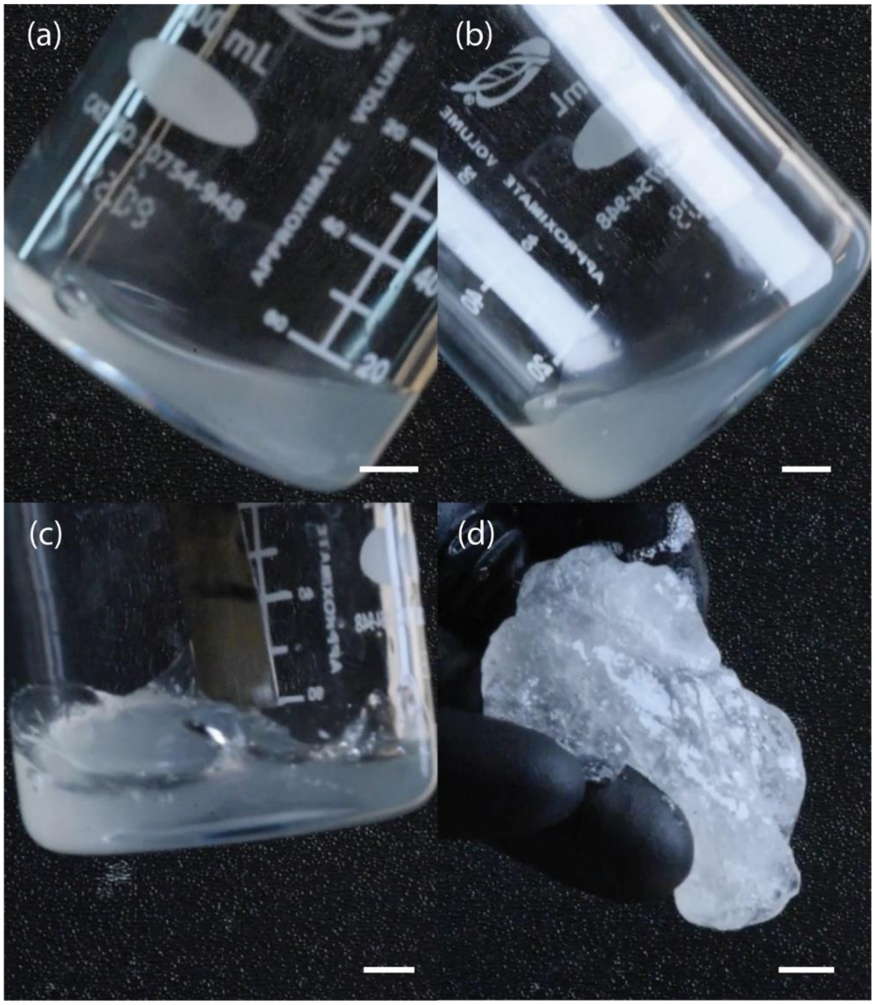

Figure S10. Visual confirmation crosslinks of hydrogels after mixing. (a) and (b) shows the flowable precursor mixture before sodium borate addition; (c) 4 seconds after sodium borate addition shows significant increases in viscosity, confirming crosslinking; (d) Well mixed final gel after letting the gel rest for 5 min. The crosslink reaction happened rapidly after sodium borate addition. All scale bars are 10 mm.

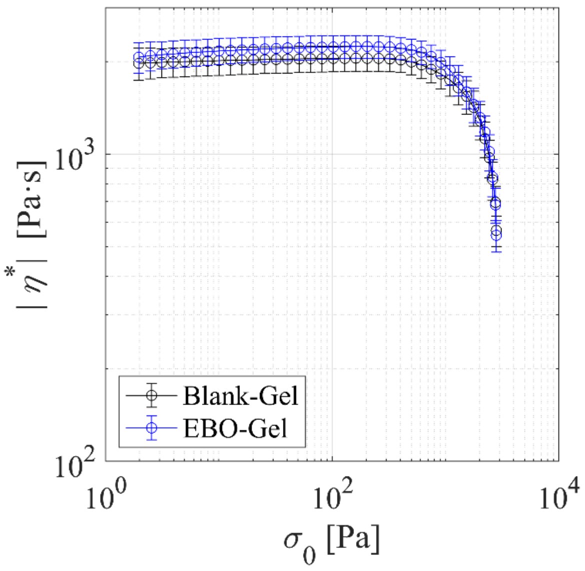

Figure S12. Magnitude of complex viscosity, $|\eta^*|$, as a function of applied stress amplitude, σ_0, showing high shear thinning behavior when σ_0 surpasses the thresholding yield stress, $\sigma_y \approx 2$ kPa, of both blank and EBO hydrogels. The ordinate data is replotted from data in Figure S11, where $|\eta^*| \equiv \omega^{-1}\sqrt{G'^2 + G''^2}$.

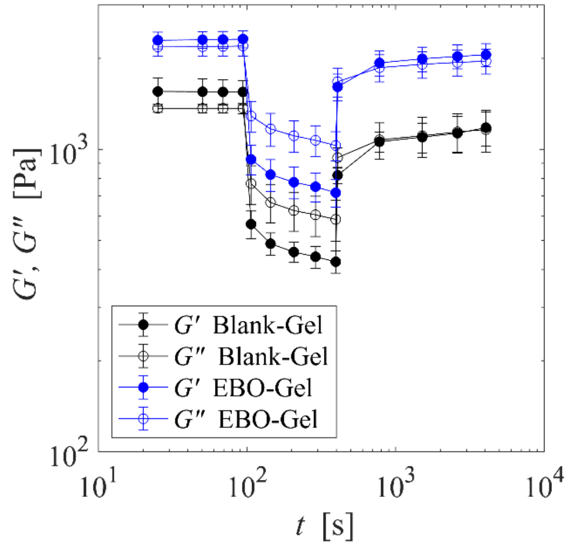

Figure S13. Nonlinear rheology recovery signifying self-healing from SAOS-LAOS-SAOS experiment at 1 rad/s. The gel shows instant recovery at the outset of second SAOS and continues aging from hydrogen bonding.

References

(1) Skedung, L., Danerlöv, K., Olofsson, U., Johannesson, C. M., Aikala, M., Kettle, J., ... & Rutland, M. W. (2011). Tactile perception: Finger friction, surface roughness and perceived coarseness. Tribology International, 44(5), 505-512.

Comment 5. Figure 3i, Need to include a high-magnification image of the blank gel for comparison. Due to the small size of exosomes, their presence is challenging to discern, and a control image would help in visual differentiation.

RESPONSE: Thank you for the comment. In response to the suggestion, we have included the updated SEM images in the revised Supplementary Information (Figure S) and manuscript (Figure 3), where red arrows are used to emphasize the rough areas with nanoscale particles on the surface. These details have been incorporated into the manuscript (Page 7, Line 181-183), and the specifics are listed below.

"SEM images reveal the porous structure and the presence of nanoscale particle in EBO-Gel (Figure 3i). In contrast to the smooth surface observed in Blank-Gel (Figure S8), EBO-Gel exhibits a rougher surface with nanoscale particles adhering to the gel skeleton, indicating the existence of EBO."

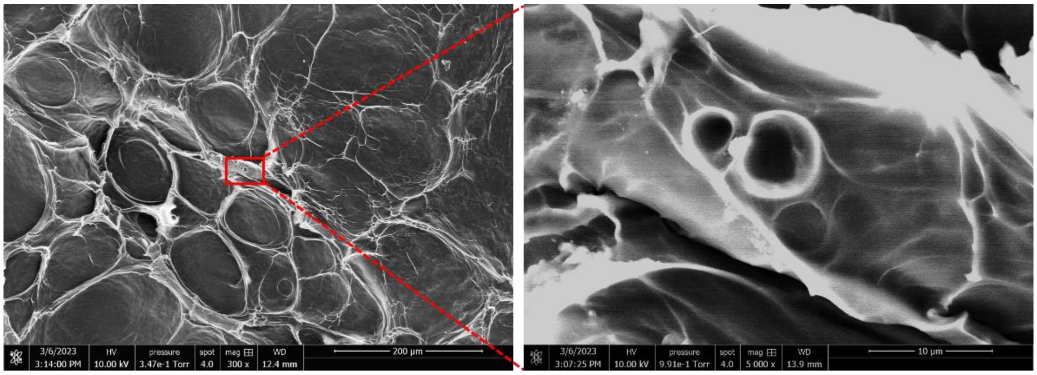

Figure S8. SEM images of Blank-Gel (left) and the magnified image (right).

Comment 6. Figure 3e, incorporating cyclic strain data would provide a more comprehensive representation of the self-healing properties of the hydrogel, demonstrating continuous property changes over time.

RESPONSE: We are pleased to provide further insights into gel characterization.

In response, we have now provided the results from cyclic strain amplitude. On a rotational rheometer, we subjected our hydrogels to linear ($\gamma_0 = 5\%$), nonlinear ($\gamma_0 = 200\%$) and linear ($\gamma_0 = 5\%$) strain (Figure S13). This figure shows recoverability from nonlinear strain, which can imply self-healing property. Moreover, the strain amplitude sweep data in Figure S11 confirms that the applied strain amplitudes are indeed in linear and nonlinear regimes.

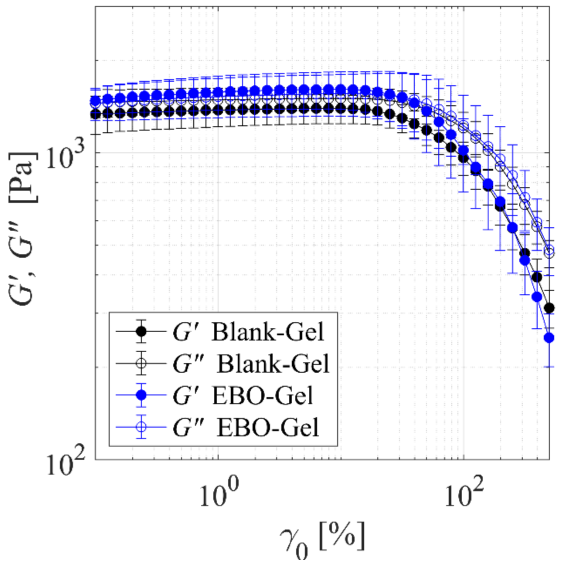

Figure S11. Strain amplitude sweep of the PVA-GA-Borax hydrogels at 1 rad/s. Critical yielding strain amplitude, γ_y, is estimated to be about 100%, marking the boundary of linear and nonlinear regimes. Subtle increases in both moduli are attributable to physical hydrogen bonds.

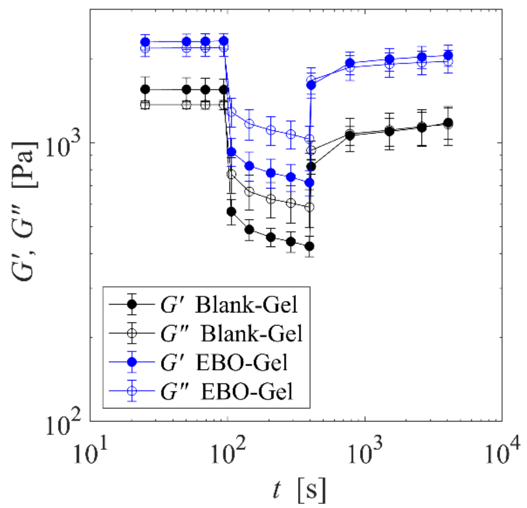

Figure S13. Nonlinear rheology recovery signifying self-healing from SAOS-LAOS-SAOS experiment at 1 rad/s. The gel shows instant recovery at the outset of second SAOS and continues aging from hydrogen bonding.

Comment 7. Figure 6d: For the in vitro hemostatic characterization, a non-hemostatic hydrogel is needed as a control group to prove that the coagulation is due to gelation/PVA, not because the gelation holds and retains the blood cells or whole blood.

RESPONSE: This is an excellent point. To answer this question, we introduced a Carbopol gel, a hydrogel based on synthetic polymers and demonstrates a non-hemostatic nature, as the control group (1). As illustrated in Figure S, the Carbopol gel shows no significant procoagulant effects, aligning with the Control group and conventional gauze group. Additionally, we compared the in vitro hemostasis ability of EBO-Gel with two commercial products, CURAD® BloodStop® Hemostatic Gauze and BleedStop™. The results indicate that EBO-Gel has similar procoagulant effects with these commercial hemostatic products, further underscoring its potential in the application of wound hemostasis.

The revised documents now incorporate the following content:

"This underscores its hemostatic capability, similar to commercial products including CURAD® BloodStop® Hemostatic Gauze and BleedStop™, in comparison to both the control group and a non-hemostatic gel (Carbopol hydrogel group) (Figure 6d and Figure S16)." (Page 12, Line 306-309)

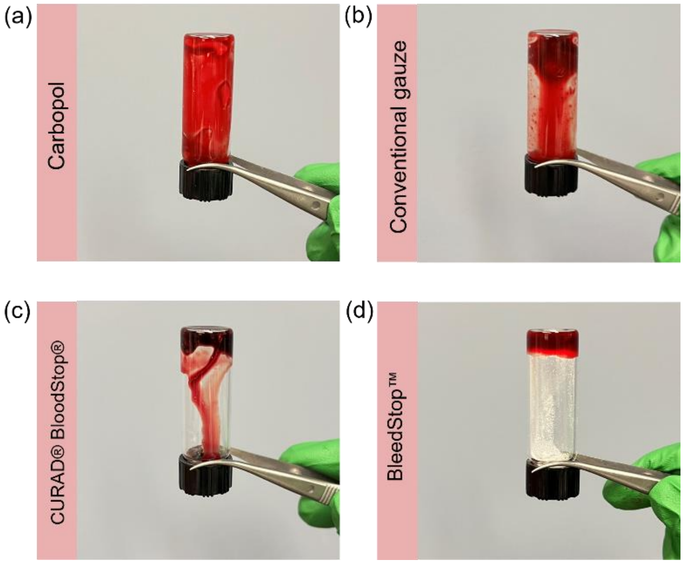

Figure S16. In vitro procoagulant effects of different treatments including (a) A non-hemostatic hydrogel, Carbopol; (b) Conventional gauze; (c) CURAD® BloodStop® Hemostatic Gauze; and (d) BleedStop™.

References

(1) Mohandas, A., PT, S. K., Raja, B., Lakshmanan, V. K., & Jayakumar, R. (2015). Exploration of alginate hydrogel/nano zinc oxide composite bandages for infected wounds. International journal of nanomedicine, 10(sup2), 53-66.

Comment 8. Line 302: In the in vivo experiment, did the author mix the precursor with borax before injection or inject them separately? What is the volume of hydrogel applied to the wounds? Considering that the hydrogel volumes applied may vary over time due to different wound closure, it is imperative to explain how this variation was controlled.

RESPONSE: We acknowledge the significance of providing this information. To clarify, in the in vivo experiment, we combined the precursor solution with the 2wt% borax solution prior to administration. The overall volume constituted 0.5 ml, comprising 0.4 ml of PVA/GA (10wt% PVA and 2.5wt% GA) and 0.1 ml of EBO. A detailed elucidation of this procedure is now available in the updated manuscript (refer to Page 22, Line 560-564) for thorough comprehension.

"The precursor solutions were prepared by thoroughly mixing 0.4 ml of PVA/GA (10 wt% PVA and 2.5 wt% GA) and 0.1 ml of nanoparticle solutions (Exo, ONB, and EBO) to achieve final concentrations of 8 wt% PVA and 2 wt% GA respectively. Hydrogels were formed by combining the precursor solution with an equal volume of 2 wt% borax solution before application."

We value this input and hope we have clarified this comment.

Comment 9. Figure 8: From the wound closure results, the most significant differences between groups can be found between day 2 and day 6. Such a time window is also the crucial inflammatory/regenerative stage in wound healing. However, the authors show all histological analyses on day 14, where wounds in all groups have closed. It is recommended that the authors provide histological analysis at an early-middle time point.

RESPONSE: We appreciate your suggestion, and in accordance with the comment, we conducted histological staining of the wound tissue collected at Day 4 post-treatment. The discussion of these new results is now provided in the revised manuscript (Page 16, Line 377-383) as follows:

"Day 4 represents a crucial time window for the inflammation and proliferation stages of the healing process, while Day 14 can reflect the regeneration stage and healing efficacy. As illustrated in Figure S19 and S20, the EBO-Gel group exhibited a consistently regenerated epidermis, increased fibroblast proliferation, enhanced angiogenesis, and collagen fiber formation. In contrast, wounds in the Tegaderm and Blank-Gel groups showed incomplete closure and higher areas of inflammation infiltration. This highlights the role of EBO-Gel in influencing the inflammation, proliferation, and early regeneration stages of healing."

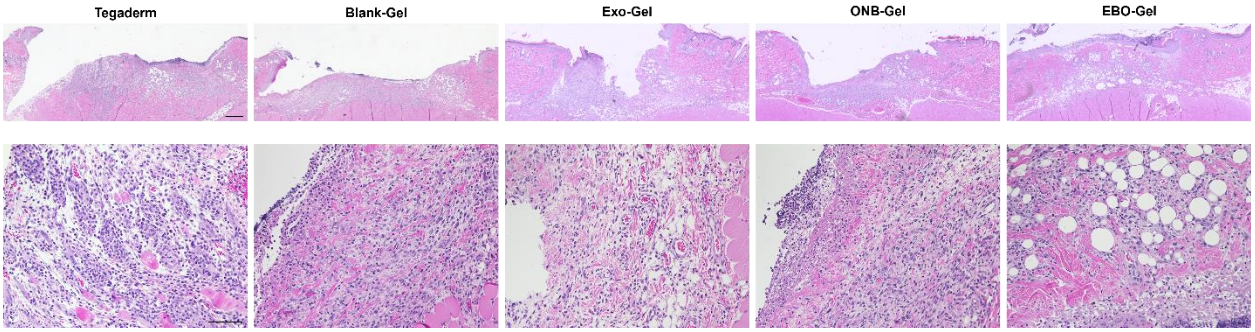

Figure S19. Representative (Row 1) and the corresponding magnified images (Row 2) of H&E staining of the wound tissues on Day 4 post-treatment. Scale bar = 500 µm for Row 1 and 100 µm for Row 2.

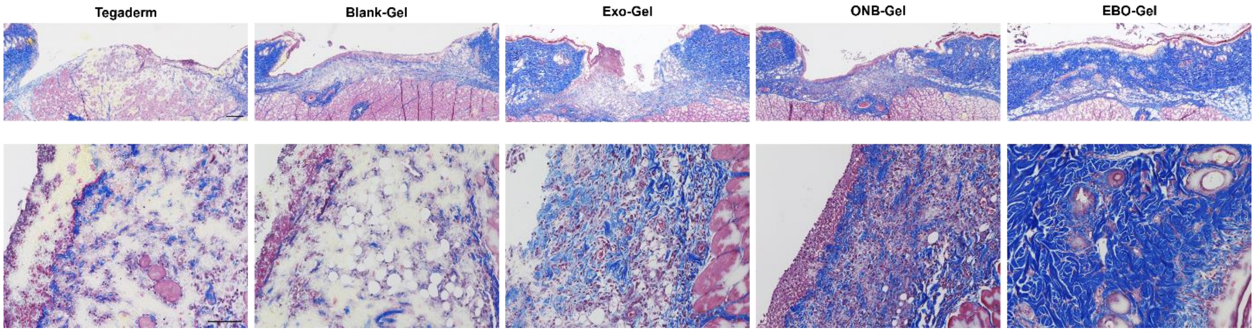

Figure S20. Representative (Row 1) and the corresponding magnified images (Row 2) of Masson's trichrome staining of the wound tissues on Day 4 post-treatment. Scale bar = 500 µm for Row 1 and 100 µm for Row 2.

Comment 10. Figure 9: The authors claim that EBO-Gel promotes angiogenesis while reducing inflammation and oxidative stress. However, H&E and trichrome staining are insufficient to substantiate claims regarding infection, inflammation (referenced in line 307), and angiogenesis. Data on immune cells and blood vessels are necessary to provide conclusive evidence.

RESPONSE: Thank you for this suggestion. We analyzed inflammation and angiogenesis using tissue fluorescent staining of CD31, DHE, CD86, CD206, and IL-6. We have discussed the results in the revised manuscript as follows (Page 17, Line 407-422).

"To assess the multifaceted impact of EBO-Gel on angiogenesis and anti-inflammatory responses in vivo, we performed fluorescent staining of wound tissues on Day 14 post-treatment. The immunofluorescent staining for CD31, a marker indicating angiogenesis, exhibited heightened expression and fluorescence intensity in the EBO-Gel treated group, showcasing its potential in promoting angiogenesis (Figure 9g and S23). By incorporating ADSC-exosomes, EBO-Gel has the potential to mitigate excessive inflammation, forestalling the development of dysfunctional scars throughout the healing process. Our examination encompassed ROS levels, inflammatory cytokine expression, and macrophage phenotypes. Dihydroethidium (DHE) staining unveiled significantly lower ROS levels in wounds treated with EBO-Gel in comparison to the Tegaderm control and Blank-Gel (Figure 9h and S23). Notably, the heightened DHE intensity in the Tegaderm control and Blank-Gel groups suggests prolonged elevated ROS levels in healed tissue even after 14 days of treatment, possibly contributing to hypertrophic scar formation. In the evaluation of immune cells through immunofluorescent staining for CD86 and CD206 with F4/80—markers for M1 inflammatory and M2 anti-inflammatory macrophages, respectively—EBO-Gel demonstrated lower CD86 and higher CD206 expression compared to control groups (Fig. 9i, S23-25). Furthermore, the immunofluorescent staining of interleukin-6 (IL-6), an inflammatory marker, exhibited the lowest expression in the EBO-Gel group, solidifying its efficacious anti-inflammatory effects (Figure S26 and S27)."

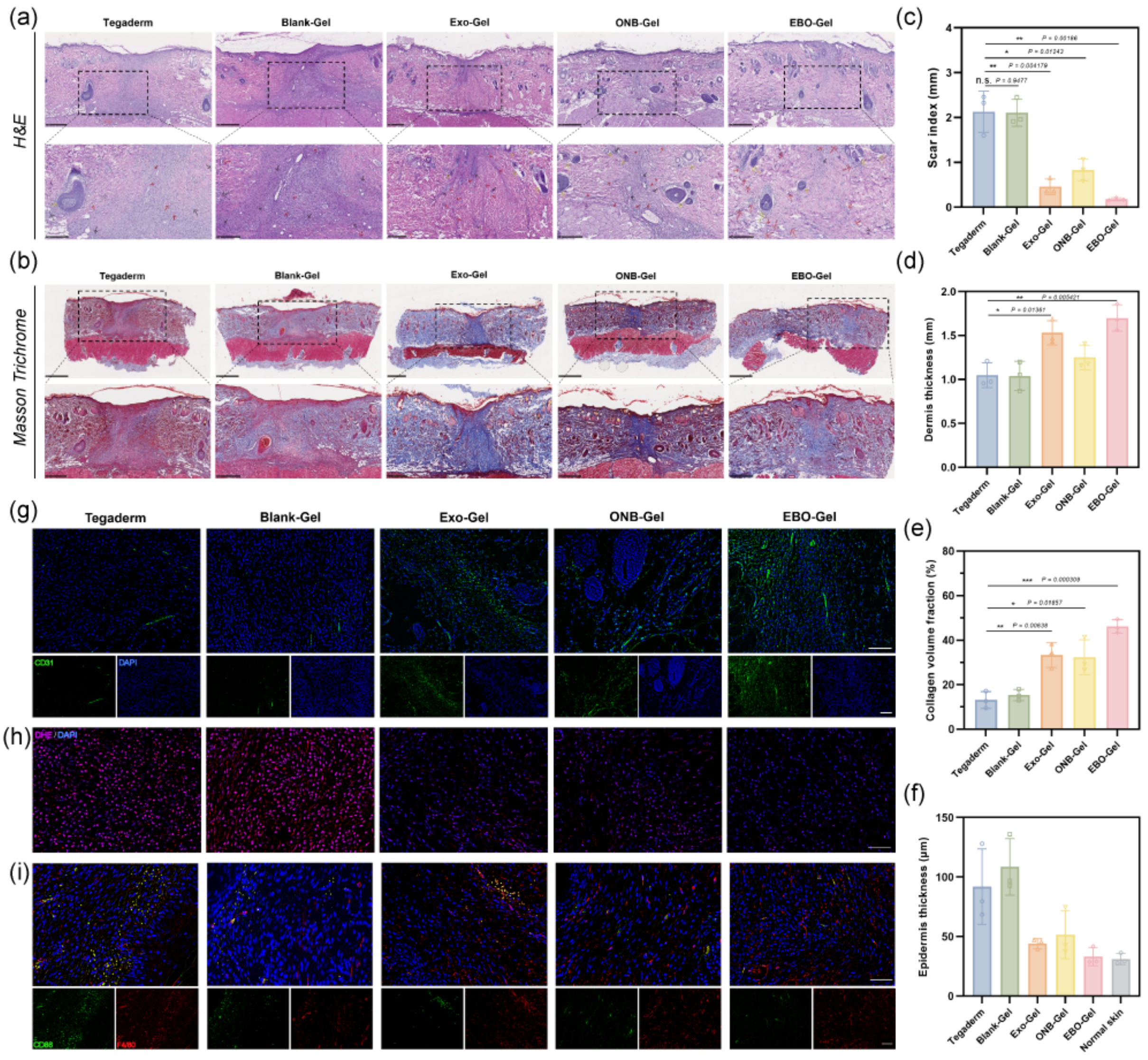

Figure 9. histological analysis of wounds that underwent treatments with different hydrogels.

Representative images of (a) H&E and (b) Masson's trichrome staining of the wound tissue on Day 14. Red arrows: new blood vessel formation; Black arrows: inflammation area; Yellow arrows: newly generated-hair follicles. Scale bar: 500 μm for the normal-sized image, and 250 μm for the magnified image. Various parameters for wound healing evaluation, including: (c) Scar index; (d) Dermis thickness; (e) Epidermis thickness; and (f) Collagen volume fraction (n = 3). Representative fluorescence images of (g) CD31 immunostaining, (h) DHE staining, and (i) CD86 and F4/80 immunostaining of wound tissues on Day 14 post-treatment. Scale bar = 100 μm (CD31) and 50 μm (DHE and CD86).

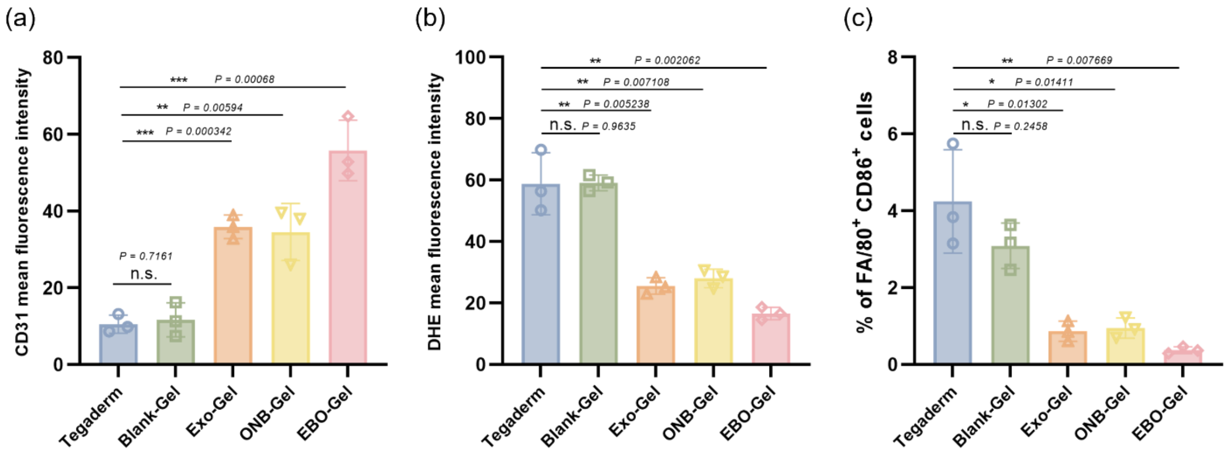

Figure S23. Quantification of fluorescent intensity of (a) CD31 and (b) DHE (n = 3, mean ± SD). (c) Quantification of the percentage of M1 macrophages (n = 3, mean ± SD).

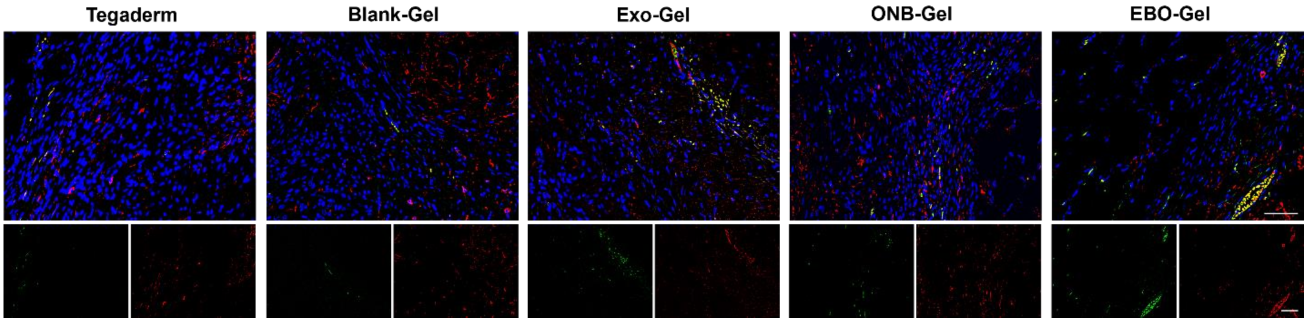

Figure S24. Wound tissue immunofluorescent staining of CD206 (green) and F4/80 (red). Scale bar = 50 µm.

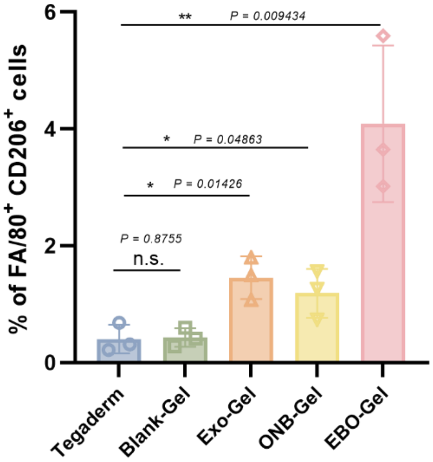

Figure S25. Quantification of the percentage of M2 macrophages (n = 3, mean ± SD).

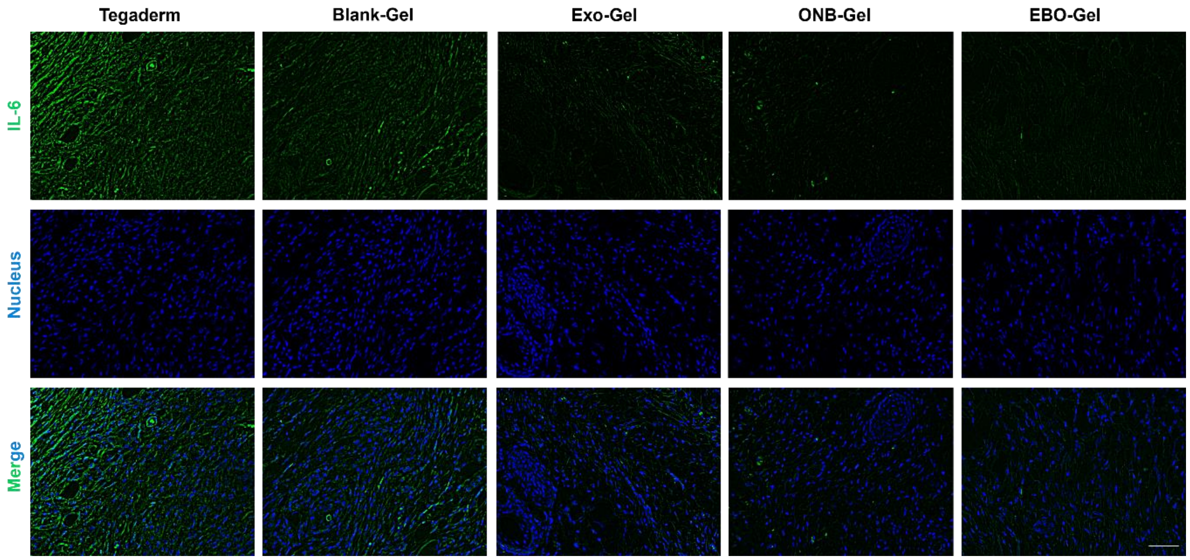

Figure S26. Wound tissue immunofluorescent staining of IL-6. Scale bar = 50 µm.

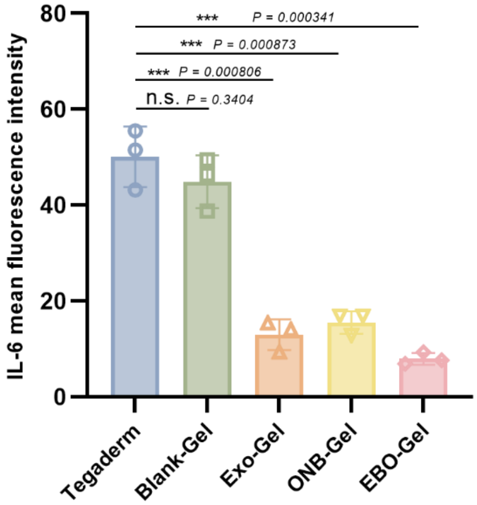

Figure S27. Quantification of fluorescent intensity of IL-6 staining (n = 3, mean ± SD).

Comment 11. Line 457: In all in vitro experiments, the authors use 3% O2 as their hypoxic condition. Is this concentration physiologically related to healthy and wounded dermis? It is suggested that the authors discuss how the extent of hypoxia affects wound healing, since many studies reported that hypoxia is a stimulating factor in tissue regeneration.

RESPONSE: This is an excellent point. Indeed, the selection of 3% O_2 in our in vitro experiments is based on the consideration of the physiological oxygen levels in wound tissues, as reported in the literature (1-

3). Notably, studies have highlighted the hypoxic nature of wound tissue, exhibiting varying oxygen concentrations across different regions (1). The central area of a wound tends to be more hypoxic, with an oxygen gradient that progressively increases towards the uninjured tissue at the periphery (2). Quantitatively, the partial pressure of oxygen (pO_2) in dermal wounds ranges from 0 to 10 mm Hg at the center and increases to 60 mm Hg at the periphery (1,3). This corresponds to oxygen concentrations of 1.4% to 8.4% O_2, compared to the atmospheric oxygen level of approximately 20% (160 mmHg). Consequently, our deliberate choice of 3% O_2 establishes a controlled hypoxic environment, aligned with the reported physiological range thus ensuring consistency across our experimental procedures.

We acknowledge that short-term hypoxia can serve as a potential stimulant in the initial phase of tissue regeneration, as rightly pointed out by the reviewers' comment. However, prolonged hypoxia throughout the entire healing process can impede optimal recovery. Initial acute hypoxia triggers a transient increase in vascular endothelial growth factor (VEGF), stimulating human dermal fibroblast proliferation and angiogenesis (4). However, long-term hypoxia in subsequent healing stages impedes the angiogenic process, leading to impaired healing. While hypoxia initiates neovascularization, it cannot be sustained (5).

Studies showed that VEGF expression increases in both hypoxic and hyperoxic environments, but efficient angiogenesis requires sufficient oxygen. Another study shows chronic hypoxia-induced neovascularization inhibition persists despite added VEGF, indicating the significance of restoring oxygen levels after the initial phase of acute hypoxia in the healing process (6). Moreover, molecular oxygen is essential for collagen synthesis and protocollagen maturation, lacking in oxygen scarcity, compromising extracellular matrix (ECM) formation, and impeding endothelial migration (7). Oxygen therapy was demonstrated to positively influence acute (8) and chronic wound healing (9).

Additionally, the emergence of keloids and hypertrophic scars are adverse events caused by surgery, trauma, or burns, which complicates wound recovery (10). Keloid formation disrupts regenerated tissue function, significantly impacting affected individuals' overall quality of life. Studies demonstrate lower oxygen tension during hypertrophic scar progression (~5-50 mmHg) (10), suggesting the potential of oxygen supply strategies to prevent hypertrophic scar formation.

We have included a comprehensive overview of these insights in the revised manuscript and are thankful for this comment which we believe has enriched the discussion of mechanisms. The following text is now available in the Discussion Section.

The following text is now available in the Discussion Section.

Page 20, Line 459-464:

"Although acute hypoxia can stimulate initial tissue regeneration by increasing vascular endothelial growth factor (VEGF), prolonged hypoxia hinders optimal recovery[53]. While hypoxia initiates neovascularization, it cannot sustain the process[54], emphasizing the importance of restoring oxygen levels after the initial acute hypoxia phase in wound healing. Moreover, lower oxygen tension during hypertrophic scar progression suggests oxygen supply as a potential strategy to expedite wound healing and prevent hypertrophic scar formation[55]."

References

(1) Sen, C. K. (2009). Wound healing essentials: let there be oxygen. Wound repair and regeneration, 17(1), 1-18.

(2) Castilla, D. M., Liu, Z. J., & Velazquez, O. C. (2012). Oxygen: implications for wound healing. Advances in wound care, 1(6), 225-230.

(3) Gordillo, G. M., & Sen, C. K. (2003). Revisiting the essential role of oxygen in wound healing. The American journal of surgery, 186(3), 259-263.

(4) Rodriguez, P. G., Felix, F. N., Woodley, D. T., & Shim, E. K. (2008). The role of oxygen in wound healing: a review of the literature. Dermatologic surgery, 34(9), 1159-1169.

(5) Sheikh, A. Y., Rollins, M. D., Hopf, H. W., & Hunt, T. K. (2005). Hyperoxia improves microvascular perfusion in a murine wound model. Wound repair and regeneration, 13(3), 303-308.

(6) Hopf, H. W., Gibson, J. J., Angeles, A. P., Constant, J. S., Feng, J. J., Rollins, M. D., ... & Hunt, T. K. (2005). Hyperoxia and angiogenesis. Wound repair and regeneration, 13(6), 558-564.

(7) Hong, W. X., Hu, M. S., Esquivel, M., Liang, G. Y., Rennert, R. C., McArdle, A., ... & Longaker, M. T. (2014). The role of hypoxia-inducible factor in wound healing. Advances in wound care, 3(5), 390-399.

(8) Chandra, P. K., Ross, C. L., Smith, L. C., Jeong, S. S., Kim, J., Yoo, J. J., & Harrison, B. S. (2015). Peroxide‐based oxygen generating topical wound dressing for enhancing healing of dermal wounds. Wound Repair and Regeneration, 23(6), 830-841.

(9) Chen, H., Cheng, Y., Tian, J., Yang, P., Zhang, X., Chen, Y., ... & Wu, J. (2020). Dissolved oxygen from microalgae-gel patch promotes chronic wound healing in diabetes. Science advances, 6(20), eaba4311.

(10) Zheng, J., Song, F., Lu, S. L., & Wang, X. Q. (2014). Dynamic hypoxia in scar tissue during human hypertrophic scar progression. Dermatologic Surgery, 40(5), 511-518.

Comment 12. Line 507: Please explain why the hydrogel needs to be applied to the wound every two days. Are the hydrogels fully degraded within 2 days? Were old hydrogels removed before applying new ones? Detailed information on this aspect is needed in the experiment section.

RESPONSE: We appreciate the opportunity to provide further clarity on our choice of a 48-hour interval for dressing refreshing.

For the first and second questions:

Our decision was informed by a comprehensive evaluation of key features of EBO-Gel, including its 48-hour oxygen release behavior, nanoparticle release profile, and in vitro and in vivo degradation characteristics. The analysis of oxygen release demonstrated a sustained dissolved oxygen (DO) concentration above Control-Gel levels for 40 hours, supporting the suitability of a 48-hour dressing interval (SI: Page 17, Figure S15). Additionally, the EBO release profile revealed over 80% of nanoparticle release after 48 hours, reinforcing the rationale for our chosen interval (SI: Page 12, Figure S9).

In addition, EBO-Gel degrades by ~80% in 3 days under the in vitro mimic physiological conditions and fully degrades within the same timeframe when injected into rats' subcutaneous tissue, ensuring both biosafety

and timely EBO release in vivo (SI: Page 16, Figure S14). Considering animal welfare, particularly in the rat models, the 48-hour dressing interval was chosen during the 14-day treatment period to balance the need for optimal oxygen utilization with ethical considerations. Daily changes would necessitate increased distress to the animals due to repeated anesthesia. Therefore, the chosen interval aligns with both the efficacy of EBO-Gel and ethical standards for animal care.

For the third question:

Yes, this is correct. Old hydrogels were completely removed during dressing changes before applying new ones. Recognizing the need for further details on animal study procedures, we have provided a comprehensive description in the "Method-in vivo wound healing study" section. The updated manuscript (Page 22-23, Line 565-569) now includes the following text:

"The prepared hydrogels were applied to the wounds post-surgery. The wound surface was ultimately covered with a Tegaderm (4.4 × 4.4 cm^2, 3M™ Tegaderm™ 1622W) and a self-adhering elastic bandage (Equate™). Dressings were refreshed every two days following the standard of care. The old residue hydrogels were entirely removed before applying freshly prepared hydrogels, followed by covering with new Tegaderm and bandage, as described before."

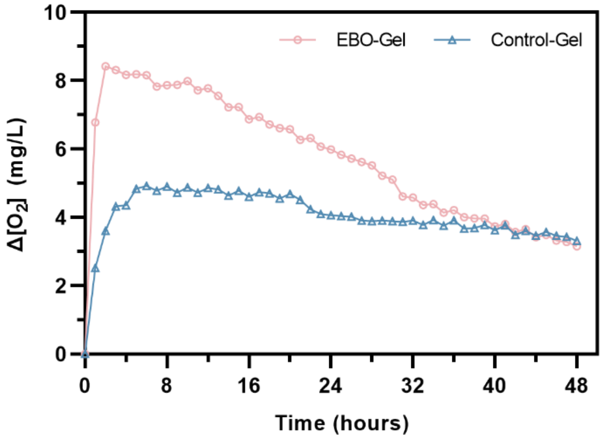

Figure S15. Oxygen concentration curve of EBO-Gel and Control-Gel (Blank-Gel with oxygenated exo/BSA/Dex solution) in 48 hrs.

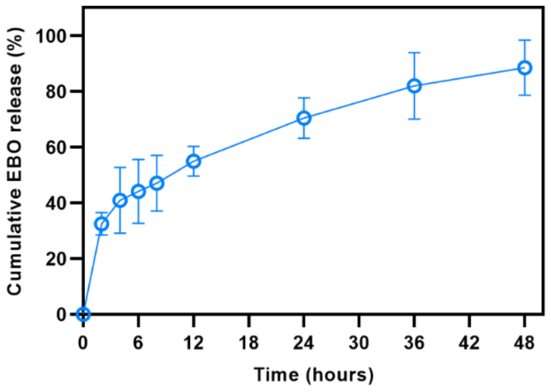

Figure S9. EBO nanoparticle release profile from EBO-Gel within 48 hrs at a pH of 7.4, 37 °C. (n = 3, mean ± SD)

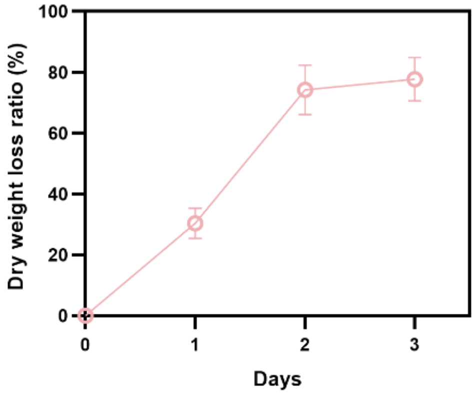

Figure S14. In vitro degradation rate of EBO-Gel within 3 days in PBS at 37 °C. (n = 3, mean ± SD)

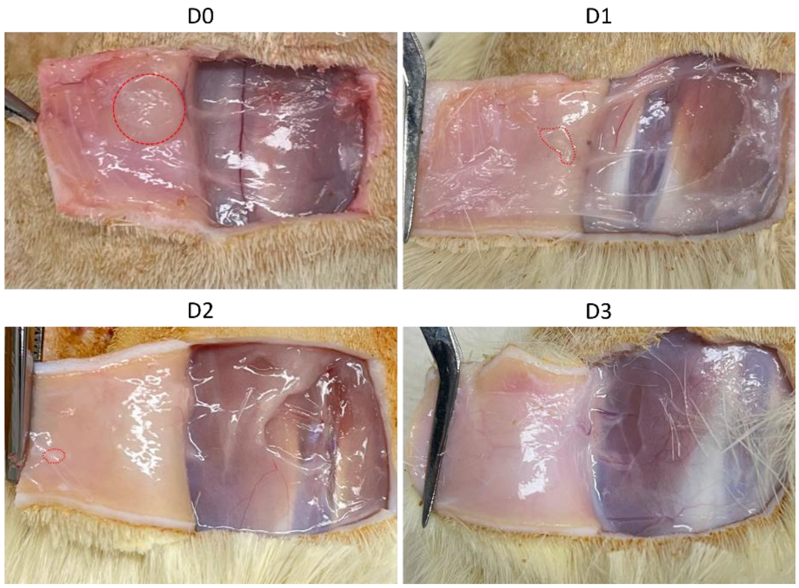

Figure S28. Overall observation of the in vivo degradation of EBO-Gel within 3 days. (Residual gel is indicated by the red area)

Comment 13. The manuscript should elucidate the mechanisms by which EBO-Gel treatment facilitates scarless healing.

RESPONSE: We are thankful for highlighting this important aspect. The potential mechanisms by which EBO-Gel promotes scarless healing are discussed in the revised manuscript (Page 20, Line 470-474), with further elaboration provided below:

Adipose-derived stem cell exosomes (ADSC-Exos) have emerged as a promising therapeutic with the ability of modulating key pathways to prevent hypertrophic scar formation. ADSC-Exos, enriched with regulatory microRNAs such as miR-192-5p and miR-29a, play a pivotal role in mitigating fibrotic responses (1).

Throughout the wound healing process, the influence of ADSC-Exos on dermal fibroblast migration is orchestrated by the regulation of a specific long non-coding RNA (lncRNA) known as MALAT1 (metastasis-associated lung adenocarcinoma transcript 1), a pivotal component within ADSC-Exos (2). Notably, as the healing progresses to the later stages, ADSC-Exos negatively modulates fibroblast migration, inhibiting the progression of fibrosis. This modulation serves as a crucial mechanism preventing the transition from physiological scars to pathological scars, including keloids and hypertrophic scars.

Growing evidence has highlighted the pivotal involvement of exosome-enriched microRNAs in the process of visceral fibrosis and tissue regeneration. MiR-192-5p targets the Smad pathway, effectively inhibiting fibroblast migration and the transdifferentiating of fibroblasts into myofibroblasts (1). Simultaneously, miR-29a intervenes with hypertrophic scar fibroblasts, impeding fibrosis by targeting the TGF-β2/Smad3 signaling pathway (1). Furthermore, ADSC-Exos hinders fibroblast differentiation into myofibroblasts, promotes apoptosis in keloid fibroblasts, and suppresses key genes associated with scar formation (3). This comprehensive regulatory network regulated by ADSC-Exos presents a multifaceted approach to curtail hypertrophic scar formation, offering potential therapeutic strategies in wound healing and scar modulation.

Another possible mechanism involves the oxygen-supplying capability of EBO-Gel. Research has confirmed the presence of a hypoxic environment (oxygen tension ~5-50 mmHg) in hypertrophic scars, likely arising from the abnormal metabolic conditions of pathological scar tissue (4). Additionally, findings show a reduction in the growth of keloid fibroblasts under hyperbaric oxygenation (HBO) therapy (5), indicating that investigating oxygen supply could be a valuable new direction in scar management.

The revised manuscript (Page 20, Line 470-474) now includes the following text in discussion section:

"The scarless healing potential of EBO-Gel is attributed to the multifaceted functions of ADSC-Exos, enriched with microRNAs such as miR-192-5p and miR-29a that regulate fibrotic responses and keloid fibroblast migration56. Moreover, EBO-Gel shows promise in addressing abnormal metabolic conditions and oxygen tension in hypoxic hypertrophic scars[57,58], offering a promising avenue for further investigation in scar management."

References

(1) Li, Y., Zhang, J., Shi, J., Liu, K., Wang, X., Jia, Y., ... & Hu, D. (2021). Exosomes derived from human adipose mesenchymal stem cells attenuate hypertrophic scar fibrosis by miR-192-5p/IL-17RA/Smad axis. Stem Cell Research & Therapy, 12, 1-16.

(2) Cooper, D. R., Wang, C., Patel, R., Trujillo, A., Patel, N. A., Prather, J., ... & Wu, M. H. (2018). Human adipose-derived stem cell conditioned media and exosomes containing MALAT1 promote human dermal fibroblast migration and ischemic wound healing. Advances in wound care, 7(9), 299-308.

(3) Li, C., Wei, S., Xu, Q., Sun, Y., Ning, X., & Wang, Z. (2022). Application of ADSCs and their exosomes in scar prevention. Stem Cell Reviews and Reports, 1-16.

(4) Ichioka, S., Ando, T., Shibata, M., Sekiya, N., & Nakatsuka, T. (2008). Oxygen consumption of keloids and hypertrophic scars. Annals of plastic surgery, 60(2), 194-197.

(5) Romero-Valdovinos, M., Cárdenas-Mejía, A., Gutiérrez-Gómez, C., Flisser, A., Kawa-Karasik, S., & Ortiz-Monasterio, F. (2011). Keloid skin scars: the influence of hyperbaric oxygenation on fibroblast growth and on the expression of messenger RNA for insulin like growth factor and for transforming growth factor. In Vitro Cellular & Developmental Biology-Animal, 47, 421-424.

REVIEWERS' COMMENTS

Reviewer #1 (Remarks to the Author):

I think the revised work can be accepted in its current form.

Reviewer #2 (Remarks to the Author):

No additional comments

Reviewer #3 (Remarks to the Author):

The revision has addressed all of our concerns and we recommend publishing as it is.
